# Supplementary material for: Barriers and facilitators to accessing tuberculosis care in Nepal: a qualitative study to inform the design of a socioeconomic support intervention
Source: BMJ Open. 2021 Oct 1;11(10):e049900. doi: 10.1136/bmjopen-2021-049900 (PMC8488704; doi:10.1136/bmjopen-2021-049900)
Supplement: Supplementary data [file bmjopen-2021-049900supp001.pdf]

Supplementary File I: Focus group discussion coding framework

Category 1: TB, health, and basic education

**TB, health, and basic education barriers**				
**FGD**	**First order category**	**Second order themes**	**Third order themes**	**Example Quote**
People diagnosed with TB	TB, health, and basic education	Inadequate knowledge on TB services	Information barrier to access	**FGD with people diagnosed with TB, 20-25 years age group, female:** *"I didn't know that medicines were free in hospitals. I knew it only when I visited health post."*
People diagnosed with MDR-TB	TB, health, and basic education	Limited knowledge on TB disease	Information barrier to access	**FGD with people diagnosed with MDR-TB, 70-75 years age group, male:** *"No one told me anything [about TB] but I knew that it was a communicable disease."*
Community leaders	TB, health, and basic education	Inadequate advocacy on TB services	Information barrier to access	**FGD with community leaders, 45-50 years age group, male:** *"There is no person to advocate about [available health and TB] services."*
People diagnosed with MDR-TB	TB, health, and basic education	Limited knowledge on TB disease and TB medicines dosages	Information barrier to adhere to TB services	**FGD with people diagnosed with MDR-TB, 45-50 years age group,male:** *"I took medicine for 2 months and then left for 15 days because I thought I was feeling better."*
Civil society organization	TB, health, and basic education	Lack of awareness raising activities particularly in poorer and	Information barrier to access	**FGD with civil society organization, 45-50 years age group, male:** "*In addition, programmatic TB awareness-raising activities were seen as scarce, especially among poorer, "educationally and socially marginalized."*

		socially marginalized		
Civil society organization	TB, health, and basic education	Inadequate awareness raising educational materials	Information barrier to access	**FGD with civil society organization, 45-50 years age group, male: "***There has been few educational materials development for TB awareness at present in comparison to the past. Therefore, people do not go to the health facility."*
**TB, health, and basic education facilitators**				
**FGD**	TB, health, and basic education	**Second order theme**	**Third order theme**	**Quotes**
Civil society organization	TB, health, and basic education	Improvements in basic education on TB	Education on TB disease and advocacy about its services	**FGD with civil society organization, 45-50 years age group, male***: "TB education should be spread intensively to village households."*
People diagnosed with TB	TB, health, and basic education	Awareness about TB disease, treatment and prevention	Education on TB disease and advocacy about its services	**FGD with people diagnosed with TB, 30-35 years age group, male:** *The government should develop TB awareness raising programs….[including] in the media, to tell people about TB symptoms, check-ups….. and that it's a curable disease."*

People diagnosed with TB	TB, health, and basic education	Community engagement<br><br>Mobilization of TB champions and peers	Education on TB disease and advocacy about its services	**FGD with people diagnosed with TB, 30-35 years age group, male:** *"People previously affected by TB could tell their story to community and mother's groups. I am 100% sure this will work."*
People diagnosed with TB	TB, health, and basic education	Awareness about TB<br><br>Information on TB medications	Education on TB disease and advocacy about its services	**FGD with people diagnosed with TB, 45-50 years age group, male:** *"People in the villages will take medicines regularly if they are educated….the government should strengthen education on TB medication."*
Civil society organization	TB, health, and basic education	Community engagement<br><br>Mobilization of community leaders for TB awareness	Education on TB disease and advocacy about its services	**FGD with civil society organization, 50-55 years age group, female:** *"We need to create [educational] groups attached to health centres and schools….Community and locally-elected leaders and teachers could give education to their communities and conduct TB awareness training and workshops."*
TB healthcare professional	TB, health, and basic education	Awareness about TB<br><br>Communication to influence health beliefs and change behaviour	Education on TB disease and advocacy about TB services	**FGD with TB healthcare professional, 55-60 years age group, male:** *"Changing [people's] attitude [about TB] is one of the things we can start doing to….. make a change in [people's] behaviour."*

Community Mobilizers	TB, health, and basic education	Awareness about TB<br>Improved communication to influence health beliefs and change behaviour	Education on TB disease and advocacy about TB services	**FGD with Community Mobilizers, 30-35 years age group, male:** *"[Some] patients don't believe us when we tell them that they have been diagnosed with TB. They say they'll go to India for check-up. In our region, people who believe in a certain religion think that TB will cure itself."*
People diagnosed with TB	TB, health, and basic education	Advocacy on TB services	Expansion of TB services in community	**FGD with people diagnosed with TB, 30-35 years age group, male: "***Health facilities should conduct door to door campaign. [Health facility] should collect sputum in a timely manner. That's it, actually there is a lack of advocacy.***"**
Community leaders	TB, health, and basic education	Prioritize underserved people	Education on TB disease and advocacy about TB services in remote communities	**FGD with community leaders, 65-70 years age group, male: "***One of the reasons is that many people do not have adequate information because in this era radio, television, mic, seminars, workshops are organized where people/ community are literate. But those people who do not have access to any of these media are disadvantaged from these programs. So, we should focus more to those people as well."*

Category 2: Social protection and nutrition

**Social protection, nutrition, and economic barriers**

FGD	First order categories	Second order themes	Third order themes	Quote
People diagnosed with TB	Social protection, nutrition, and economic	Out-of-pocket expenses for transportation to engage with treatment	Economic barrier to treatment adherence	**FGD with people diagnosed with TB, 55-60 years age group, male**: *"When I don't have money, I cannot ride auto [local vehicle] because it costs Rs 300 [$2.5 USD] to reach home or clinic. I can't earn three pennies! How can I pay Rs 300 to go to home?"*
People diagnosed with TB	Social Protection, nutrition and economic	Out-of-pocket expenses for diagnostic tests	Economic barrier to access	**FGD with people diagnosed with TB, 45-50 years age group, male:** *"I visited all the pharmacies in my city, about 15-17 medicals [pharmacies] overall. I used to buy pneumonia medicine and take it every time. TB was not initially identified. After a month without taking any medicines, TB was identified [at the government clinic]….. The barrier is more financial. I spent approximately 1.7 lakhs [~1530 USD] for my treatment."*
Civil society organization	Social Protection, nutrition and economic	Out-of-pocket expenses for transportation and nutrition	Economic barrier to treatment adherence	**FGD with civil society organization, 50-55 years age group, female:** *"TB medicines are free but people also need costs for two-way transportation, food. TB illness [and even] TB treatment can make people weak and nutrition is needed. How can people afford [nutrition and transport costs]?"*
People diagnosed with MDR-TB	Social Protection, nutrition and economic	Lost income<br>Reduced productivity related to TB illness<br>Psychological stress	Economic barrier to treatment adherence	**FGD with people diagnosed with MDR-TB, 20-25 years age group, male:** *I used to be a conductor on a bus but I can't do any work now. I suffer headaches just going to the kitchen to cook rice. How can such a person work?"*

People diagnosed with MDR-TB	Social Protection, nutrition and economic	Lost income<br><br>Reduced productivity related to TB illness	Economic barrier to treatment adherence	**FGD with people diagnosed with MDR-TB, 40-45 years age group, male:** *"I used to work outside but after being affected by MDR-TB, I stopped going to work. I didn't earn money"*
People diagnosed with TB	Social Protection, nutrition and economic	Out-of-pocket expenses for accessing TB services<br><br>Borrowing money as coping strategies	Economic barrier to access and adhere to TB treatment	**FGD with people diagnosed with TB, 25-30 years age group, male:** *"I had difficulties [to pay money to access TB services] and wasn't able to go [to the clinic] for a month….I had to borrow money from my friends."*
People diagnosed with TB	Social Protection, nutrition and economic	Out-of-pocket expenses for accessing TB services<br><br>Selling assets as coping strategies	Economic barrier to treatment adherence	**FGD with people diagnosed with TB, 20-25 years age group, male:** *"I can't pay back the loan [I took out during treatment], which causes problems…..we might have to sell assets to pay back the loan."*
People diagnosed with MDR-TB	Social Protection, nutrition and economic	Inadequate financial support	Economic barrier to treatment adherence	**FGD with people diagnosed with MDR-TB, 20-25 years age group, male:** *"The TB hostel where I am staying assured us that they would give money but they still haven't [up to 20 months into treatment]."*
**Social protection and nutrition**				
**FGD**	**First order categories**	**Second order themes**	**Third order themes**	**Quote**
Civil society organization	Social Protection,	Provision of nutrition or cash incentives	Nutrition and or economic support	**FGD with civil society organization, 50-55 years age group, female:** "*When patients take medicine, they will feel weak but they will not have the facility of proper*

	nutrition and economic		to treatment adherence	*nutritious food. Thus, if certain amount of money is made available to them, then it will help them."*
Community leaders	Social Protection, nutrition and economic	Delivery mechanism and timing of incentives	Economic support to treatment adherence	**FGD with community leaders, 45-50 years age group, male:** *"The question is when to give the allowance. It would be better in the first phase [of treatment] because it is [most] valuable at the time one most needs it."*
People diagnosed with TB	Social Protection, nutrition and economic	Provision of economic and nutrition support<br>Prioritization of nutrition and economic support	Nutrition and or economic support to treatment adherence	**FGD with people diagnosed with TB, 30-35 years age group, male:** *"Government should provide nutritious food based on the economic status of patients……drug-sensitive patients should also be provided with an allowance based on their socio-economic condition."*

Category 3: Psychosocial barriers

FGD	First order categories	Second order themes	Third order themes	Quote
Female: diagnosed with TB	Psychosocial category	Stigma<br>Enacted stigma in the community<br>Perceived stigma<br>Lack of knowledge	Social barrier to adhere during treatment	**FGD with female: diagnosed with TB, 25-30 years age group, female:** *"I have not told anyone in my family about my disease because they will hate me. I fear that if my family or friends know about my disease, they'll start talking behind my back and not come near me."*

Civil society organization	Psychosocial category	Stigma<br><br>Enacted stigma in the community<br><br>Perceived stigma<br><br>Lack of knowledge	Social barrier to access and adhere during treatment	**FGD with civil society organization, 45-50 years age group, male:** *"People don't want to sit close to or make contact with someone with TB. They can act scared when they're near a patient with TB. Ironically, the same society promotes TB treatment and encourages people with TB to get themselves tested and treated."*
People diagnosed with TB	Psychosocial category	Stigma<br><br>Enacted stigma in the community<br><br>Perceived stigma<br><br>Lack of knowledge	Social barrier to access	**FGD with people diagnosed with TB, 30-35 years age group, male:** *"People may know they have symptoms of TB but are too ashamed to go to the healthposts. People can't say out loud that they have TB. TB is regarded as a big disease and people get criticized for having it. The community perceives a TB patient differently than a normal person due to lack of awareness. That's why it's difficult to end TB."*
Healthcare professional	Psychosocial category	Stigma<br><br>Enacted stigma in the community<br><br>Perceived stigma<br><br>Lack of knowledge<br><br>Limited social support	Social barrier to adhere to treatment	**FGD with TB healthcare professional, 30-35 years age group, male:** *"Some people have very low social support, including from their family. Those people are at risk of not taking a full course of TB treatment."*

People diagnosed with MDR-TB	Psychosocial category	Psychological impact<br><br>Stress, anxiety, and depression	Psychological barrier to treatment adherence	**FGD with people diagnosed with MDR-TB, 45-50 years age group, male:** *"After seeing the healthcare workers, I felt angry and depressed. I didn't want to stay with others, not even with my family. I just sat alone."*
People diagnosed with MDR-TB	Psychosocial category	Psychological impact<br><br>Stress, anxiety, and depression	Psychological barrier to treatment adherence	**FGD with people diagnosed with MDR-TB, 20-25 years age group, male:** *I felt depressed at first. No one in my clan had suffered from this disease. I wondered how I'd got affected."*
People diagnosed with MDR-TB	Psychosocial category	Psychological impact<br><br>Stress, anxiety, and depression	Psychological barrier to treatment adherence	**FGD with people diagnosed with MDR-TB, 45-50 years age group, male:** *"I wanted to die. One of my friends [with MDR-TB] committed suicide after 16 months [of treatment]."*
**Category 3b: Psychosocial facilitators**				
**FGD**	**First order categories**	**Second order themes**	**Third order themes**	**Quote**
emale: diagnosed with TB	Psychosocial category	Family care and support	Mutual support as facilitator to treatment	**FGD with female: diagnosed with TB, 40-45 years age group, female:** *"When I restarted taking medicines, it was very difficult for me. My husband helped me at that time and, slowly, I started getting better."*

People diagnosed with MDR-TB	Psychosocial category	Peer support and motivation	Mutual or social support as facilitator to treatment	**FGD with people diagnosed with MDR-TB, 40-45 years age group, male:** *"My friends and the people in my village told me TB is normal disease and encouraged me that if I took my medicine, I'd be alright."*

Category 4: Healthcare system, TB diagnosis and care delivery

Category 4a: Healthcare system, TB diagnosis and care delivery barriers				
**FGD**	**First order categories**	**Second order categories**	**Third order categories**	**Quote**
TB healthcare professional	Healthcare system, TB diagnosis and care delivery	Delay in diagnosis<br><br>Self-perception of disease and choice of treatment<br><br>Repeated visits for diagnosis<br><br>Inadequate referral mechanisms	Convoluted pathway to diagnosis as barrier to access	**FGD with TB healthcare professional, 45-50 years age group, male:** *"When people feel sick [with TB symptoms], they start by using herbal medication at home. When they do not get relief from that medication, they go to Dhami/Jhakri [traditional healers], then the pharmacy, then to a government clinic. So, from the start of their illness to getting the right treatment, is around 1-2 months."*
TB healthcare professional	Healthcare system, TB diagnosis and care delivery	Inadequate treatment regulation<br><br>Under-recognition of TB symptoms<br><br>Inadequate referral mechanisms	Unregulated policy for medicines distribution	**FGD with TB healthcare professional, 55-60 years age group, male:** *"If I have a cough, the local pharmacy will give me amoxicillin. [If I continue to cough] I might get a more expensive antibiotic like cefixime. If I tell the pharmacist that I am coughing up blood - a clear sign in a place like Nepal that this could be TB - I might get some bigger, more expensive antibiotics but the thought would not be about tuberculosis. The pharmacist should tell me to get checked for TB but instead I will get sold antibiotics. There are thousands of pharmacies [like this] in South Asia."*

Community Mobilizers	Healthcare system, TB diagnosis and care delivery	Geographical inaccessibility to reach TB centers<br><br>Natural disasters<br><br>Centralised location of health facilities	Geographical barriers to access TB diagnosis and care	**FGD with Community Mobilizers, 30-35 years age group, female:** *"In rainy season, flooding makes it difficult for patients to go and get medicine and [health posts] are inaccessible"*
Community Mobilizers	Healthcare system, TB diagnosis and care delivery	Geographical inaccessibility to reach TB centers<br><br>Poor quality of road and transportation network	Geographical barriers to access TB diagnosis and care	**FGD with Community Mobilizers, 20-25 years age group, male:** *"One of the main reasons for not taking medicine is because the health posts are far away….and difficult to reach….especially when the rivers get bigger and wider; they can sweep people away. If the health post was nearer, it would be easier."*
Civil society organization	Healthcare system, TB diagnosis and care delivery	Poor healthcare services<br><br>Limited or inconvenient opening times of health facilities	Inadequate services from health centers as barrier to adhere to treatment	**FGD with civil society organization, 50-55 years age group, female:** *"If we don't go on time, we don't meet the doctor. The patients must reach the hospital clinic before 11 and take a ticket.*
Community leaders	Healthcare system, TB diagnosis and care delivery	Geographical inaccessibility to reach TB centers<br><br>Poor quality of road and transportation network	Geographical barriers to access TB diagnosis and care	**FGD with community leaders, 35-40 years age group, male:** *"The health facilities are far and also may be the difficult road."*

Community leaders	Healthcare system, TB diagnosis and care delivery	Geographical inaccessibility to reach TB centers	Geographical barriers to access TB diagnosis and care	**FGD with community leaders, 45-50 years age group, male:** *"They are far from the access of Health facilities. Due to this reason they do not get medicine regularly."*
People diagnosed with TB	Healthcare system, TB diagnosis and care delivery	Poor healthcare services<br><br>Limited human resources	Inadequate services from health centers as barrier to adhere to treatment	**FGD with people diagnosed with TB, 25-30 years age group, male:** "*There are no health workers. It's such a small hospital [where I attend for TB medicine], there are five employees but four of them don't work. When I go to find and take my medicines, they say they don't know and ask me to look for it myself. The main doctor is in a meeting all the time, he doesn't know."*
TB healthcare professional	Healthcare system, TB diagnosis and care delivery	Poor healthcare services	Inadequate patient centric care as barrier to treatment adherence	**FGD with TB healthcare professional, 55-60 years age group, male:** *"If we try to put ourselves in the patient's shoes, be a TB patient, do you think it would be possible for us to visit the health centre for six months? Ruin our careers and everything to attend the health centre? We need to implement innovative ideas to support treatment adherence that are patient-centred rather than program-centred."*
People diagnosed with TB	Healthcare system, TB diagnosis and care delivery	Poor TB service delivery	Care not being patient-centred acting as barrier	**FGD with people diagnosed with TB, 20-25 years age group, male:** *"I stayed for one hour in queue and then waited a long time for*

			to treatment adherence	*treatment, spent money, and got a delayed report."*
Female: diagnosed with TB	Healthcare system, TB diagnosis and care delivery	Poor TB service delivery	Care not being patient-centred acting as barrier to treatment adherence	**FGD with female: diagnosed with TB, under 20 years age group, female:** *"Getting a delayed test report was a barrier for me, we had to come and go [to health facilities] for 4 days."*
People diagnosed with MDR-TB	Healthcare system, TB diagnosis and care delivery	Poor TB service delivery	Care not being patient-centred acting as barrier to treatment adherence	**FGD with people diagnosed with MDR-TB, 45-50 years age group, male:** *"Our community mobiliser told us to go health post and they will administer the injection [injectable medication for TB]. I went but they told me that they couldn't give me an injection at that time."*
Community leaders	Healthcare system, TB diagnosis and care delivery	Poor TB service delivery	Care not being patient-centred acting as barrier to treatment adherence	**FGD with community leaders, 45-50 years age group, male:** *"The behaviour of NTP healthcare workers needs to change. There's a difference between saying 'your father' and 'your respected father' isn't it? Imagine how bad an ill person will feel….when spoken to in an ill-mannered way; the speech will feel like an arrow."*
TB healthcare professional	Healthcare system, TB diagnosis and care delivery	Poor TB service delivery	Care not being patient-centred acting as barrier to treatment adherence	**FGD with TB healthcare professional, 55-60 years age group, male:** *"The perception of lack of patient friendly services was also acknowledged as a problem in the NTP and patient FGDs and that although there are 4200 DOTS centres and many microscopic and GeneXpert centres, many are not patient*

				friendly and this can contribute to delayed initiation of treatment."
People Diagnosed with TB	Healthcare system, TB diagnosis and care delivery	Poor TB service delivery	Care not being patient-centred acting as barrier to treatment adherence	**FGD with people diagnosed with TB, 25-30 years age group, male:** *"They don't ask anything. They just give medicines and give a tick mark in the card. It is just this at our place. They make a register. But if the main doctor [health worker] is not there, they don't even do it. They ask to take medicines by ourselves. In our village, we are asked to take medicines from the box."*
Category 4b: TB diagnosis and care delivery facilitators				
**FGD**	**First order categories**	**Second order themes**	**Third order themes**	**Quotes**
Civil society organization	Healthcare system, TB diagnosis and care delivery	Standardization of TB treatment	Proper regulation and management of TB services delivery	**FGD with civil society organization, 45-50 years age group, male:** *"If TB medicines are only available from registered governmental health facilities, people will take them from there."*

People diagnosed with TB	Healthcare system, TB diagnosis and care delivery	Standardization of TB treatment	Proper regulation and management of TB services delivery	**FGD with people diagnosed with TB, 30-35 years age group, male:** *"The main point is TB medicine is available in pharmacies and those who can pay will get it because pharmacies want money. Pharmacies don't care much about treatment and cure….or if patients take medicines at the right time or at all. That is why….TB medicines should only be provided under the supervision of government health facilities only. This will help to control TB."*
People diagnosed with MDR-TB	Healthcare system, TB diagnosis and care delivery	Improved access to TB diagnostic and treatment services	Provision of community based healthcare services and patient-centred care	**FGD with people diagnosed with MDR-TB, 40-45 years age group, male:** *"What I like the most is they come to our houses to collect sputum."*
TB healthcare professional	Healthcare system, TB diagnosis and care delivery	Availability of diagnostic services	Early diagnosis of TB	**FGD with TB healthcare professional, 30-35 years age group, male:** *"GeneXpert, X-ray and symptoms are three things that the program is focusing on."*

Community leaders	Healthcare system, TB diagnosis and care delivery	Improved access to TB diagnostic and treatment services	Provision of community based healthcare services and patient-centred care	**FGD with community leaders, 45-50 years age group, male:** "We conduct screening camps for other diseases so why can't we do screening camp for tuberculosis? In the past, NGOs collected sputum from households where there was a patient on the basis of exposure history in family members. If we could collect and screen by understanding the patient's location that easier for them or visiting their workplace, it might increase the number of cases identified."
Female: diagnosed with TB	Healthcare system, TB diagnosis and care delivery	Improved access to TB diagnostic and treatment services	Provision of community based healthcare services and patient-centred care	**FGD with female: diagnosed with TB, 45-50 years age group, female:** *"It's good to bring medicine at home. Sometimes, we have work at the time when we have to go to take medicines."*
Female: diagnosed with TB	Healthcare system, TB diagnosis and care delivery	Good behavior of healthcare providers	Patient-centred care	**FGD with female: diagnosed with TB, 60-65 years age group, female:** *"The doctor treated me like a normal patient, cared for me, asked how I was feeling and gave me medicine."*

Community Mobilizers	Healthcare system, TB diagnosis and care delivery	Good behavior and communication between patient and healthcare providers	Patient-centred care	**FGD with Community Mobilizers, 40-45 years age group, male:** *"We provide time to patients….meet them on their way to work or when coming back from work…..they are in constant contact with us…..we consult them properly…they trust us more [than doctors]."*
Female: diagnosed with TB	Healthcare system, TB diagnosis and care delivery	Proper communication between patient and healthcare providers	Patient-centred care	**FGD with female: diagnosed with TB, 45-50 years age group, female: "***Doctor told that medicines should be taken for six months completely."*
TB healthcare professional	Healthcare system, TB diagnosis and care delivery	Standardization of TB treatment and referral services	Proper regulation and management of TB services delivery	**FGD with TB healthcare professional, 30-35 years age group, male:** *"Government hospitals should mandate pharmacies and private providers to refer people with TB to government services but it was noted that legislation was insufficient."*
People diagnosed with TB	Healthcare system, TB diagnosis and care delivery	Standardization of TB treatment and referral services	Proper regulation and management of TB services delivery	**FGD with people diagnosed with TB, 30-35 years age group, male: "***TB medicines should only be provided under the supervision of government health facilities only. This will help to control TB."*

TB healthcare professional	Healthcare system, TB diagnosis and care delivery	Decentralization of advanced diagnostic services in	Expansion of advanced diagnostic services	**FGD with TB healthcare professional, 55-60 years age group, male:** *"Decentralization of GeneXpert had revolutionized the TB diagnosis in the hills of Nepal to do better diagnosis and find MDR TB roll out GeneXpert."*
TB healthcare professional	Healthcare system, TB diagnosis and care delivery	Prioritization of vulnerable groups for increased diagnosis	Strategies and policies for TB diagnosis	**FGD with TB healthcare professional, 55-60 years age group, male: "***I think we have to change our strategy of active case finding, based on the level of vulnerability and the level of risk that a person can be exposed to. If we are following the same strategy for prisoners that we are following in the community, we might not find many cases."*
