# Supplementary material for: Barriers and facilitators to accessing tuberculosis care in Nepal: a qualitative study to inform the design of a socioeconomic support intervention
Source: BMJ Open. 2021 Oct 1;11(10):e049900. doi: 10.1136/bmjopen-2021-049900 (PMC8488704; doi:10.1136/bmjopen-2021-049900)
Supplement: Supplementary data [file bmjopen-2021-049900supp002.pdf]

Supplementary File II: Healthcare system, TB diagnosis and care delivery barriers and facilitators

During thematic analysis, it was noted that the FGD participants identified "higher level" barriers and facilitators to accessing and engaging with TB services including governmental policy, political commitment, public-private mix, and healthcare infrastructure that would not be directly modifiable through a socioeconomic support intervention for TB-affected households. While important and informative, the research team felt that such themes could not practically be addressed by – and hence would not directly inform the design of - a discrete socioeconomic support package for TB-affected households for future trial evaluation. Here, the perceived higher level barriers and facilitators are mapped to Category IV "Healthcare system, TB diagnosis and care delivery" of the adapted WHO treatment adherence framework.[1] These barriers and facilitators are summarized in Figure 2 of the main article and expanded on in more detail below.

Theme: Healthcare system, TB diagnosis and care delivery barriers

Across FGDs, participants reported limited staffing at health centers, poor geographical access, unsatisfactory quality of the TB services and multiple visits in public and private health centers for diagnosis and treatment as the major barriers to accessing and engaging with TB treatment. Participants mentioned that the incorrect diagnosis of TB caused long and convoluted journey during the diagnosis and treatment, including a public-private (PPM) mix of hospitals and clinics and pharmacies before reaching NTP diagnostic and treatment services:

> **FGD with female diagnosed with TB, 25-30 years age group, female:** *"For many times, I went to hospitals and bought medicines. My health was worsening. A doctor prescribed medicines for increased blood pressure, I had those medicines. Also, later another doctor prescribed me medicines for gastritis. I had those medicines as well. None of the medicines made me feel better. It took a long time until I was diagnosed [of TB] in a government hospital."*

The NTP stakeholders mentioned that the pharmacy services were perceived to be compromised because of the practice of prescribing antibiotics without suitable clinical or microbiological confirmation of infection. Moreover, the practice of changing to new and more expensive antibiotics was noted if the disease wasn't cured. However, such services were noted to be thriving and supported a culture of, as one NTP stakeholder noted, *"medicine dependence in South Asia"*.

> **FGD with NTP stakeholders, 55-60 years age group, male:** *"If I have a cough, the local pharmacy will give me amoxicillin. [If I continue to cough] I might get a more expensive antibiotic like cefixime. If I tell the pharmacist that I am coughing up blood - a clear sign in a place like Nepal that this could be TB - I might get some bigger, more expensive antibiotics but the thought would not be about tuberculosis. The pharmacist should tell me to get checked for TB but instead I will get sold antibiotics. There are thousands of pharmacies [like this] in South Asia."*

Participants from the FGD with NTP stakeholders raised concerns about the risk of such antibiotic practices driving increasing rates of drug-resistant TB (DR-TB) and multidrug-resistant TB (MDR-

TB) in Nepal. The practice of seeking private care initially was felt to be commonplace among those of higher socioeconomic position because of sufficient household resources coupled with a perception of private services being quicker and/or higher quality than public services and more likely to help them to become cured. Nevertheless, poorer households were reported to visit cheaper, independent private healthcare providers, some of whom were reported to have no or limited formal training, in their local village. Ultimately, it was reported across FGDs that the decision to seek private care and the level of quality of private care provided was dictated by money.

> **FGD with civil society organization, 45-50 years age group, male:** *"The majority of the patients go to medical [pharmacy]. If they have to go to district [larger referral hospital], then the expenses will be higher. If they attend health centres, the expense depends on the distance."*

Geographical barriers such as long distances to reach health facilities, weather conditions such as rain, natural disasters (landslides and flooding), difficult roads and limited transportation options were discussed in depth across FGDs. These barriers were perceived as hampering access to TB diagnosis, impeding engagement with TB treatment and DOTS, and had a knock-on impact on access to broader health services.

The participants in FGDs with community stakeholders and people with TB perceived restricted opening hours, delayed opening, limited time available for clinical investigation [in hospitals], and prolonged waiting times for consultation and receiving reports as barriers to both TB diagnosis and treatment. Community stakeholders reported that this was especially important for labourers given the competing demands to earn money during peak labour hours or seek healthcare during clinic opening hours. These barriers were compounded by issues relating to staff lateness and staff shortages or absence at healthcare centers that provided TB services:

> **FGD with people diagnosed with TB, 25-30 years age group, male:** "*There are no health workers. It's such a small hospital [where I attend for TB medicine], there are five employees but four of them don't work. When I go to find and take my medicines, they say they don't know and ask me to look for it myself. The main doctor is in a meeting all the time, he doesn't know."*

Moreover, participants perceived that there was negative behaviour of healthcare workers towards people seeking TB diagnosis or who were taking TB treatment as a notable barrier and disincentive to attend. There was a perception that some NTP staff at health centers took little interest in people with TB. A participant with TB disease mentioned that the healthcare workers at the DOTS center only provide medicines and put a tick mark in the treatment card or sometimes even proper recording is missed in the card. Another participant with TB also shared his experience during his treatment where he was often told to take medicines from the shelf at the DOTS center. There were also reports of discourteous consultations

During FGDs with people with MDR-TB, room conditions, atmosphere, and quality of TB services at MDR-TB centers were reported as not being patient-friendly and a significant barrier to engagement. Apart from waiting times, issues raised included crowded waiting rooms and

inadequacies in cleanliness and hygiene of clinics and hospitals. Although people with MDR-TB reported supportive care and staff at the DR-TB hostels in which some were managed during their treatment, others reported that the facilities were not as expected. One participant describing their room to have been *"colder than snow"* and another shared that the substandard conditions of the hostel rooms made him feel even worse and so he did not remain there. The perception of lack of patient friendly services was also acknowledged as a problem in the FGDs with NTP stakeholders and people with TB:

Theme: Healthcare system, TB diagnosis and care delivery facilitators

During the FGD with NTP stakeholders, PPM was discussed in detail and, specifically, integration of the public and private sector TB services as a potential facilitator. Both NTP stakeholders and people with TB cited that government hospitals should mandate pharmacies and private providers to refer people with TB to government services. However, it was noted that current legislative powers were insufficient to realise this:

> **FGD with NTP stakeholders, 30-35 years age group, male:** *"We can regulate the health professionals but we cannot regulate the pharmacies….where the majority of TB cases are going and taking pills."*

It was noted across FGDs that outreach and decentralized community-based services that incorporate use of advanced diagnostic tools would be beneficial to reach healthcare facilities. NTP stakeholders reported that decentralization of GeneXpert had revolutionized TB diagnosis in the hills of Nepal, increasing TB case detection while promptly identifying people with DR-TB and MDR-TB. The NTP stakeholders mandated roll out of GeneXpert across Nepal and suggested integration of GeneXpert with targeted active-case finding strategies such as TB camps and door-to-door campaigns. The participants also suggested community screening, which focused on reaching underserved, vulnerable people at high risk of TB, was appropriate. People with MDR-TB reported that such active case finding strategies had been well received:

> **FGD with people with MDR-TB, 40-45 years age group, male:** *"What I like the most is they come to our houses to collect sputum."*

In addition to reports of negative behaviour from NTP staff during treatment, participants with TB also reported experiences of positive behaviour towards them as a motivating factor, which had encouraged them to take their medicines regularly. Supportive behaviour coupled with education about medication – including side effects, importance of adherence, and duration - during initial consultations was seen as an enabler for treatment engagement and adherence. During the FGD with NTP stakeholders, it was mentioned that providing adequate time to patients, proper counseling, and consultation enable people with TB disease to complete their treatment and get cured. This was perceived to be enhanced by community outreach activities by community mobilisers and female community health volunteers to ensure patients had the best opportunity to achieve treatment success:

> **FGD with female with TB, 60-65 years age group, female:** *"The doctor treated me like a normal patient, cared for me, asked how I was feeling and gave me medicine."*

Furthermore, during the FGD with people with TB, participants mentioned the necessity of patient-centric treatment services such as taking TB-medications at home instead of daily visits to the DOTS centers, which is often time consuming.

FGD with female diagnosed with TB, 45-50 years age group, female: *"It's good to bring medicine at home. Sometimes, we have work at the time when we have to go to take medicines."*

References

1. Weaver MS, Lönnroth K, Howard SC, Roter DL, Lam CG. Interventions to improve adherence to treatment for paediatric tuberculosis in low- and middle-income countries: a systematic review and meta-analysis. Bull World Health Organ. 2015;93(10):700-711B.
