# Supplementary material for: Barriers and facilitators to accessing tuberculosis care in Nepal: a qualitative study to inform the design of a socioeconomic support intervention
Source: BMJ Open. 2021 Oct 1;11(10):e049900. doi: 10.1136/bmjopen-2021-049900 (PMC8488704; doi:10.1136/bmjopen-2021-049900)
Supplement: Author's manuscript [file bmjopen-2021-049900.draft_revisions.pdf]

BMJ Open is committed to open peer review. As part of this commitment we make the peer review history of every article we publish publicly available.

When an article is published we post the peer reviewers' comments and the authors' responses online. We also post the versions of the paper that were used during peer review. These are the versions that the peer review comments apply to.

The versions of the paper that follow are the versions that were submitted during the peer review process. They are not the versions of record or the final published versions. They should not be cited or distributed as the published version of this manuscript.

BMJ Open is an open access journal and the full, final, typeset and author-corrected version of record of the manuscript is available on our site with no access controls, subscription charges or pay-per-view fees (http://bmjopen.bmj.com).

If you have any questions on BMJ Open's open peer review process please email info.bmjopen@bmj.com

BMJ Open

Barriers and facilitators to accessing tuberculosis care in Nepal: a qualitative study to inform the design of a socioeconomic support intervention

Journal:	*BMJ Open*
Manuscript ID	bmjopen-2021-049900
Article Type:	Original research
Date Submitted by the Author:	04-Feb-2021
Complete List of Authors:	Dixit, Kritika; BNMT; Karolinska Institute, Global Public Health<br>Biermann, Olivia; Karolinska Institute, Global Public Health<br>Rai, Bhola; BNMT<br>Aryal, Tara ; BNMT<br>Mishra, Gokul; BNMT; Liverpool School of Tropical Medicine<br>Teixeira de Siqueira-Filha, Noemia; Liverpool School of Tropical Medicine; University of York<br>Poudel, Puskar; BNMT; KNCV Tuberculosis Foundation<br>Pandit, Ram ; BNMT<br>Sah, Manoj; BNMT<br>Majh, Govinda ; BNMT<br>Levy, Jens; KNCV<br>Rest, Job; KNCV<br>Gurung, Suman; BNMT; Liverpool School of Tropical Medicine,<br>Dhital, Raghu; BNMT<br>Lönnroth, Knut; Karolinska Institute<br>Squire, Bertie; Liverpool School of Tropical Medicine; Royal Liverpool Hospital, Respiratory Medicine<br>Caws, Maxine; BNMT; Liverpool School of Tropical Medicine<br>Sidney, Kristi; Karolinska Institute<br>Wingfield, Tom; Liverpool School of Tropical Medicine; Karolinska Institute
Keywords:	Tuberculosis < INFECTIOUS DISEASES, PUBLIC HEALTH, QUALITATIVE RESEARCH

SCHOLARONE™
Manuscripts

I, the Submitting Author has the right to grant and does grant on behalf of all authors of the Work (as defined in the below author licence), an exclusive licence and/or a non-exclusive licence for contributions from authors who are: i) UK Crown employees; ii) where BMJ has agreed a CC-BY licence shall apply, and/or iii) in accordance with the terms applicable for US Federal Government officers or employees acting as part of their official duties; on a worldwide, perpetual, irrevocable, royalty-free basis to BMJ Publishing Group Ltd ("BMJ") its licensees and where the relevant Journal is co-owned by BMJ to the co-owners of the Journal, to publish the Work in this journal and any other BMJ products and to exploit all rights, as set out in our licence.

The Submitting Author accepts and understands that any supply made under these terms is made by BMJ to the Submitting Author unless you are acting as an employee on behalf of your employer or a postgraduate student of an affiliated institution which is paying any applicable article publishing charge ("APC") for Open Access articles. Where the Submitting Author wishes to make the Work available on an Open Access basis (and intends to pay the relevant APC), the terms of reuse of such Open Access shall be governed by a Creative Commons licence – details of these licences and which Creative Commons licence will apply to this Work are set out in our licence referred to above.

Other than as permitted in any relevant BMJ Author's Self Archiving Policies, I confirm this Work has not been accepted for publication elsewhere, is not being considered for publication elsewhere and does not duplicate material already published. I confirm all authors consent to publication of this Work and authorise the granting of this licence.

For peer review only

Barriers and facilitators to accessing tuberculosis care in Nepal: a qualitative study to inform the design of a socioeconomic support intervention

Authors

Kritika Dixit,[1,2] Olivia Biermann,[2] Bhola Rai,[1] Tara Prasad Aryal,[1] Gokul Mishra,[1,3] Noemia Teixeira de Siqueira-Filha,[3,4] Puskar Raj Paudel, [1,5] Ram Narayan Pandit,[1] Manoj Kumar Sah,[1] Govinda Majhi, [1] Jens W. Levy,[5] Job van Rest,[5] Suman Chandra Gurung,[1,3] Raghu Dhital,[1] Knut Lönnroth,[2] S Bertel Squire,[3] Maxine Caws,[1,3] *Kristi Sidney Annerstedt,[2] *Tom Wingfield [2,6]

Affiliations

[1] Birat Nepal Medical Trust, Lazimpat-2, Kathmandu, Nepal. Tel: +9774436434

[2] Department of Global Public Health, Karolinska Institutet, 17177 Solna, Sweden

[3] Department of Clinical Sciences, Liverpool School of Tropical Medicine, Pembroke Place, Liverpool L3 5QA, UK

[4] Department of Health Sciences, University of York, Heslington York, YO10 5DD, UK

[5] KNCV Tuberculosis Foundation, 2514 JD The Hague, The Netherlands

[6] Departments of International Public Health and Clinical Sciences, Liverpool School of Tropical Medicine, Pembroke Place, Liverpool, L3 5QA, UK.

*Joint Final Authors

Corresponding Author

Dr Tom Wingfield, Senior Clinical Lecturer and Honorary Consultant Physician, Room G216, LIV-TB Collaboration, Departments of International Public Health and Clinical Sciences, Liverpool School of Tropical Medicine, Pembroke Place, Liverpool, L3 5QA, UK. Tel: +441517053736. Email: tom.wingfield@lstmed.ac.uk

Words: 4992 **Figures:** 2 **Table:** 1 **Supplementary Files:** I-III

For peer review only

ABSTRACT

Introduction

In 2019, 10 million people developed tuberculosis (TB), of whom approximately three million were never diagnosed, notified or received TB treatment. Psychosocial and economic (socioeconomic) factors, including poverty, stigma, out-of-pocket expenses, and lost income, can act as barriers to accessing TB services but remain understudied, especially in low-income settings. This study aimed to characterise the socioeconomic barriers and facilitators of accessing TB services in Nepal to inform the design of a locally-appropriate socioeconomic support intervention for TB-affected households.

Methods

From August 2018 to July 2019, we conducted a qualitative study consisting of semi-structured focus group discussions (FGDs) with purposively-selected multi-sectoral stakeholders. Seven FGDs were conducted with 54 stakeholders, including 21 people with TB, 13 community stakeholders, and 20 multidisciplinary TB healthcare professionals from the National TB Program. FGDs were transcribed verbatim and analysed using thematic analysis. Codes and themes were developed in NVivo 12 with a specific focus on socioeconomic barriers and facilitators.

Results

The perceived socioeconomic barriers to accessing TB services were: inadequate TB knowledge and advocacy; high food and transportation costs; income loss; and stigma. The perceived facilitators were: enhanced championing and awareness-raising about TB and TB services; social protection including health insurance; cash, vouchers and/or nutritional allowance to cover food and travel costs; and psychosocial support and counselling.

Conclusions

These results suggest that a support intervention that integrated TB education, psychosocial counselling, and expanded on existing cash transfer schemes would be locally-appropriate and

For peer review only - http://bmjopen.bmj.com/site/about/guidelines.xhtml

could address the socioeconomic barriers to accessing and engaging with TB services faced by TB-affected households in Nepal. The findings have been used to inform the design of a socioeconomic support intervention for TB-affected households. The acceptability, feasibility, and impact of this intervention on TB-related costs, stigma and TB treatment outcomes, is now being evaluated in a pilot implementation study in Nepal.

Keywords

Tuberculosis; barriers and facilitators; socioeconomic support; healthcare access; Nepal

For peer review only

For peer review only - http://bmjopen.bmj.com/site/about/guidelines.xhtml

Key questions

What is already known?

- Social determinants and consequences of tuberculosis (TB) are key drivers of TB prevalence, morbidity, and mortality
- Previous studies in Nepal have mostly focused on understanding the health system barriers to access to TB treatment and care

What are the new-findings?

- As part of mixed-methods research to develop a socioeconomic support intervention for TB-affected households, this qualitative study collated the opinions of key stakeholders, including people with TB, on the socioeconomic barriers to accessing and engaging with TB in Nepal
- The findings show that TB-affected households face multiple economic barriers, including nutrition expenses, travel expenses, and income loss, and social barriers, including inadequate tuberculosis knowledge and stigma.

What do the new findings apply?

- The findings contributed to the new knowledge on optimal local strategies to mitigate socioeconomic impact
- To overcome these barriers, the new knowledge generated has informed the design of a novel socioeconomic support intervention for TB-affected households in Nepal.
- The design is being implemented in a pilot evaluation funded by the Joint Global Health Trials (MR/V004832/1) for future scale-up.

For peer review only - http://bmjopen.bmj.com/site/about/guidelines.xhtml

BACKGROUND

Tuberculosis (TB) kills 1.3 million people each year worldwide, more than any other single infectious disease including, up to the time of writing, Covid-19.[1] In 2019, approximately 10 million became ill with TB, of whom 2.9 million were not notified or remained undiagnosed and untreated.[1] In low- and middle-income countries (LMICs), stigma, marginalization, and catastrophic costs of accessing TB diagnosis and care, coupled with limited social protection coverage, can delay diagnosis, decrease TB treatment success rates, and push TB-affected households into further impoverishment.[2,3] To address this and move towards TB elimination, the World Health Organization's (WHO) 2015 End TB Strategy set the bold target that "Zero TB-affected families should face catastrophic costs" and that psychosocial and economic (socioeconomic) support should be provided to TB-affected people.[4]

Nepal is a lower-middle-income country in South Asia with significant TB incidence (annual incidence 245/100,000) and mortality.[5] Despite free basic TB diagnostic tests, medicines and financial support for people with drug-resistant (DR-TB), approximately one in two people with TB face catastrophic costs (total TB-related costs >20% of a household's annual income) while accessing TB care in Nepal.[6,7] Such costs include travel for Directly Observed Treatment Short-course (DOTS), additional food expenditure, and lost income, which can contribute to adverse TB treatment outcomes, especially for the poorest, most vulnerable households.[6,8–10]

The Nepal National TB Program (NTP) provides 3,000 Nepalese Rupees (~27 USD) in cash incentives monthly for transportation and nutritional support to people with multi-drug resistant TB (MDR-TB)[11] who are enrolled in government treatment centers and receiving ambulatory care. There is currently no cash incentive scheme for people with drug-sensitive TB (DS-TB) in Nepal.[12] In other settings, socioeconomic support for TB-affected households, including mutual support groups and cash transfers, has been shown to help overcome barriers to accessing TB services, defraying catastrophic costs, and improving treatment success rates.[3,13–16] However, there is limited context-specific understanding of the barriers and facilitators to TB

For peer review only - http://bmjopen.bmj.com/site/about/guidelines.xhtml

diagnosis and care in LMICs with which to inform the development of tailored socioeconomic support interventions for people with TB and their households.[1] This study aimed to address this knowledge gap in Nepal.

MATERIALS and METHODS

Study design: We conducted an exploratory qualitative study, which used semi-structured focus group discussions (FGDs) to collate the perceptions of key stakeholders in Nepal regarding socioeconomic barriers and facilitators of accessing and engaging with TB diagnosis and care. The study formed part of a larger programme of mixed-methods research[17] to design a locally-appropriate socioeconomic support intervention for TB-affected households. The study adhered to the COnsolidated criteria for REporting Qualitative research (COREQ) Checklist (Research Checklist).[18]

Study setting: The study was implemented in four districts of Nepal where Birat Nepal Medical Trust (BNMT), a Nepalese organization with a focus on TB-related implementation research, implemented IMPACT-TB project. The districts: Makwanpur, Chitwan, Dhanusha, and Mahottari have a high prevalence of poverty and TB (Figure 1).[11] Makwanpur is a hilly district with limited road networks. Other three districts are lowland plains and challenged by high population density, poor health indicators, and high rates of illiteracy.

Figure 1. Map of population and TB case notification rates in the four study districts

(Authors suggest to insert the Figure 1 here in this section)

Sampling: A desk-based scoping exercise was initially performed by team members (KD, RD, TW) to identify participants from relevant stakeholder groups in Nepal. To collate diverse perspectives on barriers and facilitators of TB diagnosis and treatment, the team purposively selected participants who had direct or indirect experiences with TB services. The participants included: people affected by TB who were currently receiving or had recently completed DS-TB or MDR-TB treatment with the NTP (referred to as “people with TB” in the study); community leaders such as female community health volunteers, teachers, and social leaders from civil

For peer review only - http://bmjopen.bmj.com/site/about/guidelines.xhtml

society organisations (CSOs, referred to as "community stakeholders"); and TB healthcare professionals, including those working with the NTP, community volunteers, and TB-focused NGOs (referred to as "NTP stakeholders") [Supplementary File I]. Using telephone, email, or in-person meetings, we invited 55 individuals to participate in the study. Inclusion criteria were being 18 years of age or older and being able to give informed consent. Seven participants were invited to each of the seven FGDs.

Data collection: The study team consisted of diverse members from multiple sectors including a physician, senior TB researchers, social scientists, public health professionals, and project managers. An interview guide was developed by the coauthors with previous qualitative methods experience: TW (Male, Principal Investigator, TB researcher), KD (Female, doctoral student, Project Manager), and BR (Male, Public Health Specialist, Research Associate); BR and KD are employed by BNMT. The interview guide consisted of open-ended questions to explore the perceptions of participants concerning protective factors and risk factors for exposure to TB and development of TB disease; barriers and facilitators to accessing and engaging with TB diagnosis and care, including the recommendations for better access and engagement with TB diagnosis and care; and the socio-economic impact on people with TB of being ill with the disease.

Prior to conducting FGDs, participants were provided with a '*Participant Information Sheet'* that explained the purpose of the study, benefit and harm, and confidentiality.[17] The FGDs were conducted in a local hotel accessible to participants in the study districts. The topic guide was piloted with a group of seven female and male participants with TB resulting in minor refinements to the FGD structure and delivery techniques. TW moderated the FGD with TB healthcare professional and KD and BR moderated the other six FGDs. Apart from these researchers, district field staff who supported patients attended the discussions and facilitated any dialectic interpretation or contextual explanations related to access to and engagement with TB services.

We conducted seven FGDs. To encourage an environment in which participants felt comfortable and able to share their opinions and to balance gender representation, two of these FGDs were

For peer review only - http://bmjopen.bmj.com/site/about/guidelines.xhtml

specifically for females with DS-TB and female TB community mobilizers. In total, 54/55 (98%) invitees participated in the FGDs, which the project team perceived as giving sufficient information power for the study.[19] One invitee declined to participate due to lack of time. We did not conduct any follow-up discussions with participants but some of the participants attended a workshop to discuss the FGD findings, the outputs of which are published elsewhere.[12] We performed member checking in each FGD by noting key points of the discussion, summarizing them on a wall chart, and clarifying their accuracy with the group. No formal field notes were taken. The FGDs, which lasted 90-120 minutes, were all conducted in Nepali language apart from the FGD with TB healthcare professionals, which was conducted in English. FGDs were audio recorded, translated into English from Nepali language, and back-translated by an independent translator who was not part of the project team. Each FGD was concluded when the facilitators collectively felt the topics in the FGD interview guide were sufficiently explored.

Analysis: We applied thematic analysis using NVivo 12 to manage the data.[20] Two researchers (KD and TW) familiarised themselves with the data through successive reading of transcripts. Initial codes were generated, which were updated as further data became available and collated following each successive FGD. Both open and closed first-order category were used to label data within NVivo. Codes were then grouped into second-order and third-order themes (Supplementary file III). Table 1 shows an example of the analysis process of codes and themes. To better inform the design and delivery of the socioeconomic intervention for TB-affected households within the wider context of health services delivery, the first-order themes were then mapped to four levels of an adapted WHO Treatment Adherence Framework: I. TB, health, and basic education; II. Social protection and nutrition; III. Psychosocial; IV. Healthcare system, TB diagnosis and care delivery.[21] We chose to structure our analysis on the themes mapped to levels I-III of the WHO Framework because these levels were the most relevant to the study's aim of informing design and development of a socioeconomic support intervention for TB-affected households. While important, themes identified that mapped to category IV of the Framework, such as governmental policy, political commitment, public-private mix, and healthcare

For peer review only - http://bmjopen.bmj.com/site/about/guidelines.xhtml

infrastructure were perceived by the study team to be largely unmodifiable by a household-level socioeconomic support intervention. These themes are reported under health system categories and are shown in Supplementary File II.

Table 1: An example of coding from the FGDs

FGD	Quote	First-order category*	Second-order Themes	Third-order themes
People diagnosed with TB	FGD with people diagnosed with TB, 32-years-old, male: *"People get criticized for having TB. The community perceives a TB patient isn't the same as a normal person…. due to lack of awareness."*	Psychosocial	- Enacted stigma<br>- Perceived stigma<br>- Lack of knowledge	Stigma as social barrier to access
* Adapted from a World Health Organization Medication Adherence Framework[21]				

To increase trustworthiness, after all the transcripts were coded and analysed, KD and TW independently reviewed coding and themes and refined them through further debate and discussion where necessary.[22]

The study received ethical approvals from the University of Liverpool (No. 2436) and the Nepal Health Research Council (208/2018). Written consent was obtained from all participants. Confidentiality of the participants was maintained by anonymizing FGD responses, keeping any

For peer review only - http://bmjopen.bmj.com/site/about/guidelines.xhtml

paper data in a locked cabinet at BNMT's office, and securing the data in a password-protected database.

Patient and public involvement

Patient and/ or the public were not involved in the design or conduct of this research.

RESULTS

Overall, 36 codes related to eight themes were identified [Supplementary File IV]. Below, we focus only on the perceived barriers and facilitators that mapped to categories I. TB, health, and basic education, II. Social protection, and nutrition III. Psychosocial, of the adapted WHO Framework. These barriers and facilitators are shown in Figure 2.

TB, health, and basic education

Theme: Information barrier to access and adhere to TB diagnosis and treatment

Across FGDs, we identified low literacy and education about TB as a barrier to accessing TB diagnosis and engaging with TB treatment (Figure 2). Knowledge about TB including transmission, prevention, symptoms, how and where to get diagnosed, treatment regimens and duration, and the TB services available at health facilities were perceived to be limited, especially among illiterate and underserved groups or rural populations. A 19-year-old female participant with TB said: *"I didn't know that TB medicines were free in hospitals. I knew it only when I visited the health post."*

The FGD with community stakeholders suggested that this limited knowledge about TB negatively impacted engagement with TB services and treatment adherence. This was noted to potentially increase the likelihood of a delayed diagnosis, more advanced disease at presentation, and acquisition of DR-TB during treatment. The perceived lack of knowledge was predominantly felt to relate to suboptimal TB education, advocacy, and political commitment from health system and governmental leaders. Participants also reported that the current health education programs are

For peer review only - http://bmjopen.bmj.com/site/about/guidelines.xhtml

scarce and unable to reach poorer, educationally and socially marginalized communities with high TB risk.

Theme: Facilitating treatment access through education about TB disease and advocacy about TB services, especially in remote communities

Across all the FGDs, participants described the need to raise community awareness on risk factors for TB, mode of TB transmission, and TB signs and symptoms. Community stakeholders particularly felt that future awareness-raising programs would benefit by informing communities about available free TB diagnostic and treatment services in their local area and adding a component to reduce TB-related stigma. Further, NTP stakeholders stressed the importance of not only providing education but also, in the face of competing health beliefs, influencing attitudes, and promoting behaviour change.

Community stakeholders suggested that the government should take responsibility for the development and implementation of intensive household- and village-level awareness programs using technology such as smartphone applications and social media. They also recommended broadcasting educational campaigns on television adapted from similar campaigns in the field of HIV (*HIV ka sawal)* and maternal care (*Aama ko Maya)* in Nepal. However, there was concern that information transmitted through media could bypass vulnerable, poor, and marginalized populations. To overcome this, the group suggested innovative and interactive community-based approaches such as coordination with local women's group-initiated self-help enterprises, street plays, and engaging TB survivors as peer champions and educators to improve TB education. The involvement and ownership of TB education programs by community leaders, including volunteers, teachers, and community mobilizers, was also deemed important to achieve effective, decentralized delivery. Furthermore, the stakeholders acknowledged the essential role of healthcare provider-led education as part of a client-provider contract because people with TB need - and will follow - advice from healthcare providers only when that advice is relayed sensitively and understood thoroughly. Nevertheless, people with TB indicated that both

For peer review only - http://bmjopen.bmj.com/site/about/guidelines.xhtml

sensitization and education from a trusted source were key to deliver education successfully and to enable and empower communities.

FGD with community leaders, 52-year-old, female: *"We need to create [educational] groups attached to health centres and schools. Community and locally-elected leaders and teachers could give education to their communities and conduct TB awareness training and workshops."*

II. Social protection and nutrition

Theme: Social and economic barriers to accessing and engaging with TB diagnosis and treatment

Across FGDs, the participants reported food insecurity, high travel and food costs, and lost income as key barriers to accessing and engaging with TB diagnosis and treatment (Figure 2). The direct out-of-pocket costs of seeking TB diagnosis and engaging with DOTS at both public and private clinics, including food and transport, were raised repeatedly across FGDs as a significant barrier to timely diagnosis and medication adherence.

FGD with community leaders, 52-year-old, female: *"TB medicines are free but people also need money for two-way transportation and food. TB illness [and even] TB treatment can make people weak and nutrition is needed. How can people afford these [nutrition and transportation] costs?"*

Nonetheless, patient journeys were repeatedly reported as long and convoluted, including a public-private (PPM) mix of traditional medicine, pharmacies, local private healthcare providers, and larger private clinics before reaching NTP diagnostic and treatment services (Figure 2). As a result, TB affected households incurred significant expenses.

FGD with people diagnosed with TB, 46-year-old, male: *"I visited all the pharmacies in my city, about 15-17 medicals [pharmacies] overall. I used to buy pneumonia medicine and take it every time. TB was not initially identified. After a month without taking any medicines, TB was identified*

For peer review only - http://bmjopen.bmj.com/site/about/guidelines.xhtml

[at the government clinic]….. The barrier is more financial. I spent approximately 1.7 lakhs [~1530 USD] for my treatment."

Undernutrition and food insecurity were recognized as risk factors for TB and to contribute to suboptimal adherence. Across FGDs, it was noted that many TB-affected households have insufficient resources to buy nutritious food. In addition, it was perceived that lack of education negatively impacted decision-making related to nutrition to recover during TB treatment. In particular, community leaders and people with DR-TB raised concerns that the current monthly governmental nutrition allowance was insufficient to obtain nutritional food, such as meat and fruits, which is often recommended by healthcare providers. Female participants with TB mentioned a trade-off between out-of-pocket expenses to purchase the recommended nutritional food versus transportation expenses associated with DOTS at TB clinics.

Lost income was identified in the majority of FGDs as a notable barrier to accessing diagnosis and engaging with TB care, which compounded the economic impact of out-of-pocket travel and food costs. People with TB expressed guilt and stress related to having to stop work following their TB diagnosis and the fear of not getting employment again. Female participants described the impact as hardest on poorer populations, women working in the fields, and labourers, who lack the free time required to go to the health facility for daily DOTS, especially when opening hours and prime labouring hours coincide.

Community stakeholders and people with TB frequently cited that TB-affected households use coping strategies to mitigate the economic impact of TB, most commonly to obtain funds to buy food. Selling assets, such as livestock, milk, land, and jewelry, was reported as a predominant coping strategy. However, it was noted that some vulnerable patients were so poor that they have nothing to sell. Other coping strategies mentioned included borrowing money, formally and informally, which led to further economic hardship and difficulties maintaining adherence.

For peer review only

FGD with people diagnosed with TB, 25-year-old, male: *"I had difficulties [to pay money to access TB services] and wasn't able to go [to the clinic] for a month. I had to borrow money from my friends."*

Theme: Facilitating treatment adherence by people with TB through nutritional and/or economic support

Participants discussed the need for social protection including insurance, transportation allowance, and nutritional support for TB-affected people (Figure 2).

The potential for economic support to improve nutrition, defray travel and other out-of-pocket costs, and increase TB treatment success, was raised in all FGDs. It was acknowledged, particularly in FGDs with NTP stakeholders and people with TB, that the government of Nepal provides Rs 3,000/month (~USD 27 for ambulatory MDR-TB cases and Rs 1,000/month (~USD 8) for those staying at DR-TB hostels. While the NTP stipulates that this is intended as nutritional and/or transport allowance, participants noted that how the cash is spent is not monitored. In addition, issues were raised with this existing transfer scheme, including delays in delivery of the allowance.

FGD with civil society organization, 45-year-old, male: *"The most important question is when to give the allowance. It would be better in the first phase [of treatment] because it is [most] valuable at this time when one needs it most."*

There was further debate concerning whether cash or nutritional support was most appropriate. Some community stakeholders raised concerns regarding misuse of cash payments (e.g. to buy alcohol) and suggested that it would be preferable to provide nutritious food such as milk, ghee (local butter), meat, and eggs. However, females with TB perceived that any cash received would nevertheless be spent on food, primarily staple foods such as rice, to feed their household.

Finally, it was perceived across FGDs that any nutritional or economic support should either be provided to all or stratified by need rather than TB drug resistance profile.

For peer review only - http://bmjopen.bmj.com/site/about/guidelines.xhtml

FGD with people diagnosed with TB, 32-year-old, male: *"The government should provide nutritious food based on the economic status of patients. Drug-sensitive patients should also be provided with an allowance based on their level of poverty."*

III. Psychosocial

Theme: Psychosocial barriers to treatment adherence

Across all FGDs, stigma was perceived to be a significant barrier to seeking, accessing, and engaging with TB diagnostic and treatment services. People with TB described feeling discriminated against, mistreated, isolated, or hated. They reported perceptions or instances of people talking behind their back or remaining physically distanced. Participants of the FGD with community stakeholders shared that sometimes people with TB experienced extreme negative behavior such as physical or psychological mistreatment from their own family members. The situation was discussed as being even more pronounced for young married females because of a lack of personal agency within their husband's family. While this stigmatizing behaviour towards people with TB was reported to occur across socioeconomic groups, participants described that a *'blame and shame culture'* was prevalent among family members belonging to groups perceived as "higher" in the caste-related, social hierarchy. In alignment with this assertion, community mobilisers mentioned that lack of social and family support can cause people to conceal their TB status and not adhere to or complete TB treatment.

Reports of stigmatizing behaviour were not limited to the community and family members. Perceived negative behaviour of healthcare providers towards people with TB was noted across FGDs as an issue that compounded self-stigma and led to a breakdown of trust within the client-provider relationship. Although participants felt that, generally, enacted stigma had decreased in Nepalese communities, FGDs with people with TB and community stakeholders shared that people still fear TB disease, especially in rural villages.

FGD with people diagnosed with TB, 32-year-old, male: *"People may know they have symptoms of TB but are too ashamed to go to the health facilities. People can't say out*

For peer review only - http://bmjopen.bmj.com/site/about/guidelines.xhtml

loud that they have TB. TB is regarded as a big disease and people get criticized for having it. The community perceives a TB patient differently than a normal person due to lack of awareness. That's why it's difficult to end TB."

People with MDR-TB reported profound psychosocial impact including anxiety and isolation, especially during the first months of treatment. Depression, suicidal ideation, and shame related to stigma and also well-recognized side effects of MDR-TB medications were mentioned[23].

FGD with people diagnosed with MDR-TB, 47-year-old, male: *"I wanted to die. One of my friends [with MDR-TB] committed suicide after 16 months [of treatment]."*

Theme: Mutual or social support as a facilitator to treatment adherence

Social support from family and friends was perceived as a facilitator to adhering to TB treatment and becoming cured (Figure 2). This included visiting, spending time with, and showing affection towards people with TB to demonstrate solidarity and reduce feelings of isolation.

People with TB also shared the importance of mutual support beyond family and friends, including the wider community, leaders, elders, and other important local figures. The participants believed that this kind of support would help people with TB to cope and reflected the close communities and rich socio-cultural values inherent to Nepalese culture.

FGD with people diagnosed with MDR-TB, 44-year-old, male: *"My friends and the people in my village told me 'TB is a normal disease and encouraged me that, if I took my medicine, I'd be alright."*

Interactions and consultations with healthcare providers were also seen as opportune occasions to provide education and counseling to address the psychological impact of TB. FGD with community stakeholders raised the perceived importance of healthcare providers simply recognizing, acknowledging, and being understanding of patients' fears, concerns, and expectations. While healthcare provider-led counselling on medication adherence was noted to be commonplace at treatment initiation, counselling TB patients about TB-related fear, stigma,

For peer review only - http://bmjopen.bmj.com/site/about/guidelines.xhtml

depression, and anxiety was broadly overlooked. It was noted that integration of medication and psychological counselling by healthcare providers could be a suitable method to deliver clear and open information about stigma and discrimination, which could improve TB treatment adherence and completion rates and potentially support mental wellness and empowerment.

Figure 2: Barriers and facilitators to accessing tuberculosis care in Nepal

(Authors suggest to use the same color for online prints as used in the figure)

For peer review only

For peer review only - http://bmjopen.bmj.com/site/about/guidelines.xhtml

DISCUSSION

This qualitative study generated new evidence regarding barriers and facilitators to accessing and engaging with TB services in Nepal. Multi-sectoral stakeholder participants highlighted that the barriers were predominantly related to the poor socioeconomic conditions of people with TB, including lack of education and endemic poverty. The findings showed that the costs of care-seeking and clinic-based DOTS can further compound poverty and, when combined with psychological impacts including stigma and anxiety, were perceived to negatively influence access to TB services. Participants cited multiple potential socioeconomic interventions, both integrated and discrete, including TB education, economic, nutritional, and social support, to mitigate catastrophic costs of TB-affected households and support people with TB to get cured.

Knowledge and awareness about TB

Low education levels and limited awareness of TB are associated with delays in healthcare seeking.[24–26] A study in Nepal showed inadequate knowledge of TB was associated with increased likelihood of consulting traditional healers, resulting in TB diagnostic delay.[27] Our findings are also similar to other studies that suggested knowledge about TB was limited in poor, marginalized, and/or rural communities in Nepal.[8,28] This implies that any existing TB education and advocacy programs may not be reaching crucial, high-risk target groups and new approaches are required if Nepal is to end TB.

Educational support interventions that enhance knowledge about TB transmission, symptoms, treatment, and prevention, are important contributory factors in both care-seeking behaviour and treatment outcomes in diverse settings.[27,29,30] In India, the Global Fund-supported advocacy, communication, and social mobilization project, "Axshya", has made progress towards reaching underserved groups through intense community outreach and education.[31] FGD participants in our study cited a dearth of awareness-raising interventions and campaigns in recent years in Nepal. Previously, such campaigns, focused on TB and delivered at the household, health facility or community level, were perceived to increase knowledge about TB, advocate for the free TB

For peer review only - http://bmjopen.bmj.com/site/about/guidelines.xhtml

services, and empower communities to make informed choices. As highlighted by participants, in addition to commonly used platforms such as leaflets, radio, and television, future educational campaigns in Nepal would benefit from using technology such as mobile phones - which are used by over 90% households in both urban and rural areas[32] - or, where appropriate, social media.

Psychosocial impact

Of the perceived psychosocial barriers to accessing TB diagnosis and care in Nepal, stigma predominated. Participants mentioned feelings of guilt among people with TB, fear of disclosure, and experience of discrimination. This mirrors findings from diverse settings, which show that experiences of stigma are highly prevalent among people with TB and can impede access to TB services.[33–39] For example, in Zambia, a cohort study showed that anticipated and enacted stigma of people with TB resulted in delayed diagnosis, poor treatment adherence, reduced quality of life, and represented a distinct challenge to successful screening of their household contacts.[40]

It was notable that people with MDR-TB reported severe negative psychosocial impacts of their illness. These included profound feelings of anxiety, isolation related not only to their diagnosis but also to physical distance from their families, and recognized side effects of certain MDR-TB medications (e.g. Cycloserine) such as depression and despair.[41–43] Participants perceived an association between the psychosocial impact of MDR-TB and the potential for non-adherence to long, arduous treatment regimens including injectable agents. Discussion across FGDs suggested that existing medication adherence counselling delivered by NTP staff at treatment initiation would be a suitable platform upon which to integrate complementary psychosocial counselling about overcoming TB-related stigma and addressing ill mental health.

Economic burden of TB

The economic impact of accessing TB diagnosis and care was perceived to be severe. This was mainly due to high costs associated with transportation to clinics, maintaining adequate nutrition, and time and income loss. Participants with MDR-TB indicated that there was delay or

For peer review only - http://bmjopen.bmj.com/site/about/guidelines.xhtml

unavailability of the NTP's financial assistance scheme during their treatment. The financial impact of belonging to a household affected by TB was cited in FGDs as forcing households to resort to coping strategies such as taking out loans, using savings, and selling assets. These findings are in line with the rapidly growing global body of evidence relating to the economic burden of TB. Such findings suggest that coping strategies remain common and only limited progress has been made towards the WHO target of "zero TB-affected families face catastrophic costs by 2020".[1,44] TB Patient Costs Surveys conducted in various LMICs have demonstrated that a substantial proportion of TB-affected households incur catastrophic costs, which can push them into further impoverishment and contribute to adverse TB treatment outcomes.[6,45–50] Studies in Nepal demonstrated that more than 60% of TB-affected households in Nepal incurred catastrophic costs[6,7] and stark economic impact.[27,51–53]

Although this study focused on barriers amenable to interventions at the household level rather than health system level, our findings showed that when TB diagnosis and care were sought from both the public and private sector, patient pathways to TB diagnosis and care were protracted and their costs, especially out-of-pocket costs, escalated (described in detail in Supplementary File II). This finding is consistent with the findings of systematic reviews from Nepal, India and Uganda,[54–60] which also highlighted that interventions to strengthen public-private partnerships can streamline diagnostic and referral pathways and potentially increase TB notifications to the NTP.

Social and economic support

To address the psychosocial and economic impact of TB and improve TB cure and prevention rates, our findings imply a need for both social and economic support. This is supported by the results of studies in Nepal and other LMICs, which showed that providing both counseling and economic assistance to people with MDR-TB improved cure rates.[61–64] However, it must be noted that a significant proportion of people with DS-TB in Nepal experience enduring psychological, social and economic impacts of TB but receive no additional support.[6,7,12] In

For peer review only - http://bmjopen.bmj.com/site/about/guidelines.xhtml

line with a study from Ethiopia,[65] our findings also suggest that the timing of provision of financial support is important. Participants advocated for early cash support delivery in the initiation phase of treatment when they perceived it to be needed most. Involvement of family members and peers in such interventions was also noted by participants as a vital aspect of support to complete TB treatment. This has also been reported in a systematic review of factors affecting medication adherence in LMICs.[66]

A well-designed socioeconomic support intervention would ideally be tailored or stratified to the individual or household needs of a person with TB and include overlapping elements such as increasing knowledge, awareness-raising, cost mitigation (e.g. through cash transfers or transport vouchers) and stigma-reduction activities (e.g. mutual support, peer groups, enhanced medication counselling sessions), integrated into existing TB services.[67,68] However, it must be acknowledged that such an intervention would need to balance stratification with feasibility and pragmatism.

Strengths and limitations

Our study fills an important gap in knowledge about household-level socio-economic barriers to accessing TB services in Nepal and expanded on perceived facilitators and enablers to overcome these barriers. A major strength of the study methods was the trustworthiness and validity harnessed by garnering perspectives of the diverse study participants.[22]

The study has several limitations. As the participants were predominantly from Terai plains districts the findings, therefore, should be cautiously applied in other settings or countries. Nevertheless, we described the study setting to improve transferability. Second, participants from NGOs and healthcare professionals working with the NTP were over-represented within the participant cohort. However, we tried to strike a suitable balance by including the views of people with TB and community groups, which historically have been overlooked in similar research. FGDs consisted of limited women participants. We minimized the issue by specifically describing the female participants' viewpoint in the analysis. Similarly, there was no participation of people

For peer review only - http://bmjopen.bmj.com/site/about/guidelines.xhtml

diagnosed with TB in private sectors or those who had been loss to follow-up who are the vulnerable to restricted access to TB services and poor outcomes. Their engagement would enable us to fully understand the most important barriers to care.[45,68]

CONCLUSION

There are multiple socioeconomic barriers to accessing and engaging with TB services in Nepal. TB education and advocacy, economic support, and psychosocial counselling integrated with medication adherence counselling could address these barriers and potentially reduce stigma, mitigate TB-related costs, and improve TB treatment outcomes. These elements are now being incorporated into the design of a locally-appropriate socioeconomic support intervention for TB-affected households for pilot implementation in Nepal.

review only

For peer review only - http://bmjopen.bmj.com/site/about/guidelines.xhtml

Abbreviations

BNMT Birat Nepal Medical Trust

DOTS Directly Observed Treatment Shortcourse

DS-TB Drug sensitive TB

MDR-TB Multi Drug Resistant-TB

NTP National Tuberculosis Program

SDG Sustainable Development Goal

TB Tuberculosis

Acknowledgment

We would like to acknowledge National Tuberculosis Control Center, Ministry of Health and Population, Nepal Health Research Council, Health Directorate of Province 2 and Province 3, and all related health facilities and authorities for their support and collaboration with this project. Most importantly we are grateful to people with TB for their generosity of time and sharing their feedback and experiences during this study.

Competing interest: Authors declare no competing interest

Contributors:

Kritika Dixit (KD): Data Curation, Investigation, Project Administration, Methodology, Analysis, First draft preparation, Writing- Review & Editing

Olivia Biermann (OB), Kristi Sidney Annerstedt (KSA): Methodology, Writing – Review & Editing

For peer review only - http://bmjopen.bmj.com/site/about/guidelines.xhtml

Bhola Rai (BR), Puskar Raj Poudel (PRP): Data Curation, Project Administration, Writing – Review & Editing

Tara Prasad Aryal (TPA), Ram Narayan Pandit (RNP), Manoj Kumar Sah (MKS), Govinda Majhi (GoM): Investigation, Project Administration, Writing – Review & Editing

Gokul Mishra (GM): Investigation, Methodology, Supervision, Writing – Review & Editing

Noemia Teixeira de Siqueira-Filha (NTSF): Data Curation, Formal Analysis, Investigation, Methodology, Project Administration, Writing – Original Draft Preparation, Writing – Review & Editing

Jens W. Levy (JWL), Job van Rest (JvR): Data Curation, Resources, Validation, Writing – Review & Editing

Suman Chandra Gurung (SCG), Raghu Dhital (RG): Investigation, Project Administration, Resources, Supervision, Writing – Original Draft Preparation, Writing – Review & Editing

Knut Lönnroth (KL), **S Bertel Squire (SBS):** Conceptualization, Methodology, Supervision, Writing – Original Draft Preparation, Writing – Review & Editing

Maxine Caws (MC): Conceptualization, Formal Analysis, Funding Acquisition, Investigation, Methodology, Project Administration, Supervision, Writing – Original Draft Preparation, Writing – Review & Editing

Tom Wingfield (TW): Conceptualization, Data Curation, Formal Analysis, Funding Acquisition, Investigation, Methodology, Project Administration, Supervision, Validation, Visualization, Writing – Original Draft Preparation, Writing – Review & Editing

Funding

KD receives support from the Farrar Foundation and Royal Society of Tropical Medicine and Hygiene (RSTMH) and National Institute of Health Research (NIHR). TW is supported by grants from: the Wellcome Trust, UK (209075/Z/17/Z); the Medical Research Council, Department for International Development, and Wellcome Trust (Joint Global Health Trials, MR/V004832/1), the

Academy of Medical Sciences, UK; and the Swedish Health Research Council, Sweden. All other authors (KD, OB, BR, TPA, GM, GoM, NTSF, PRP, RNP, MKS, JL, JvR, SCG, RD, KL, SBS, MC, KSA) are supported by EU Horizon2020 grant 733174 IMPACT TB.

For peer review only

For peer review only - http://bmjopen.bmj.com/site/about/guidelines.xhtml

REFERENCES

1. World Health Organization (WHO). Global Tuberculosis Report [Internet]. Geneva; 2020 [cited 2020 Oct 20]. Available from: https://apps.who.int/iris/bitstream/handle/10665/336069/9789240013131-eng.pdf?ua=1

2. Wingfield T, Tovar MA, Datta S, et al. Addressing social determinants to end tuberculosis. Lancet. 2018;391(10126):1129–32.

3. Wingfield T, Tovar MA, Huff D, et al. Beyond pills and tests: addressing the social determinants of tuberculosis. Clin Med (Northfield Il). 2016;16:s79–91.

4. World Health Organization. Implementing the End TB Strategy: The essentials [Internet]. Geneva, Switzerland: World Health Organization; 2015 [cited 2020 Sep 8]. Available from: https://www.who.int/tb/publications/2015/end_tb_essential.pdf?ua=1

5. National Tuberculosis Control Center. Nepal National TB Prevalence Survey Brief [Internet]. 2020 [cited 2020 Apr 19]. Available from: https://nepalntp.gov.np/wp-content/uploads/2020/03/NEPAL-NATIONAL-TB-PREVALENCE-SURVEY-BRIEF-March-24-2020.pdf

6. Gurung S, Dixit K, Rai B, et al. The role of active case finding in reducing patient incurred catastrophic costs for tuberculosis in Nepal. Infect Dis Poverty. 2019;8(99).

7. Gurung SC, Rai B, Dixit K, et al. Active case finding reduces household costs for people affected by tuberculosis : The IMPACT TB longitudinal costing survey in Nepal. Health Policy Plan. 2020;

8. Bam TS, Gunneberg C, Chamroonsawasdi K, et al. Factors affecting patient adherence to DOTS in urban Kathmandu , Nepal. Int J Tuberc Lung Dis [Internet]. 2006 [cited 2020 Jan 28];10(9517):270–6. Available from: http://docserver.ingentaconnect.com/deliver/connect/iuatld/10273719/v10n3/s9.pdf?expires=1605638319&id=0000&titleid=3764&checksum=74FA4BE1638619D0003281BF3196

For peer review only - http://bmjopen.bmj.com/site/about/guidelines.xhtml

64C4

9. Baral SC, Aryal Y, Bhattrai R, et al. The importance of providing counselling and financial support to patients receiving treatment for multi-drug resistant TB: Mixed method qualitative and pilot intervention studies. BMC Public Health [Internet]. 2014 [cited 2020 Jan 28];14(46). Available from: https://bmcpublichealth.biomedcentral.com/articles/10.1186/1471-2458-14-46

10. de Cuevas RMA, Lawson L, Al-Sonboli N, et al. Patients direct costs to undergo TB diagnosis. Infect Dis Poverty [Internet]. 2016 [cited 2020 Jan 28];5. Available from: https://idpjournal.biomedcentral.com/articles/10.1186/s40249-016-0117-x

11. NTC. National Tuberculosis Program Nepal. Annual report 2074/75 (2018). Vol. 75. 2018.

12. Rai B, Dixit K, Dixit K, et al. Developing feasible, locally appropriate socioeconomic support for TB-affected households in Nepal. Trop Med Infect Dis. 2020;5(2).

13. Vinicius K, Andrade F De, Nery JS, et al. Effects of social protection on tuberculosis treatment outcomes in low or middle-income and in high-burden countries : systematic review and meta-analysis Efeitos da proteção social sobre os desfechos do tratamento da tuberculose em países de renda baixa . 2018;34(1):1–18.

14. Gomes J, Oliosi N, Reis-santos B, et al. Effect of the Bolsa Familia Programme on the outcome of tuberculosis treatment : a prospective cohort study. Lancet Glob Heal. 2018;219–26.

15. Richterman A, Steer-massaro J, Jarolimova J, et al. Cash interventions to improve clinical outcomes for pulmonary tuberculosis : systematic review and meta-analysis. PLoS One. 2018;(January):471–83.

16. Hoorn R Van, Jaramillo E, Collins D, et al. The Effects of Psycho-Emotional and Socio-

For peer review only - http://bmjopen.bmj.com/site/about/guidelines.xhtml

Economic Support for Tuberculosis Patients on Treatment Adherence and Treatment Outcomes – A Systematic Review and Meta- Analysis. PLoS One. 2016;1–27.

17. Dixit K, Rai B, Prasad Aryal T, et al. Research protocol for a mixed-methods study to characterise and address the socioeconomic impact of accessing TB diagnosis and care in Nepal. Wellcome Open Res. 2020;5(19).

18. Tong A, Sainsbury P, Craig J. Consolidated criteria for reporting qualitative research (COREQ): A 32-item checklist for interviews and focus groups. Int J Qual Heal Care [Internet]. 2007 [cited 2020 Jul 22];19(6):349–57. Available from: https://doi.org/10.1093/intqhc/mzm042

19. Malterud K, Siersma VD, Guassora AD. Sample Size in Qualitative Interview Studies : Guided by Information Power. Qual Heath Res [Internet]. 2016 [cited 2020 May 11];26(13):1753–60. Available from: https://pubmed.ncbi.nlm.nih.gov/26613970/

20. Braun V, Clarke V. Using thematic analysis in psychology. Qual Res Psychol [Internet]. 2006 [cited 2020 Sep 11];3(2):77–101. Available from: https://www.tandfonline.com/doi/abs/10.1191/1478088706qp063oa

21. Weaver MS, Lönnroth K, Howard SC, et al. Interventions to improve adherence to treatment for paediatric tuberculosis in low- and middle-income countries: a systematic review and meta-analysis. Bull World Health Organ [Internet]. 2015 [cited 2020 Jul 29];93(10):700-711B. Available from: https://www.ncbi.nlm.nih.gov/pmc/articles/PMC4645428/

22. Holloway Immy, Galvin Kathleen. Qualitative research in nursing and healthcare. John Wiley & Sons; 2016.

23. Vega P, Sweetland A, Acha J, et al. Psychiatric issues in the management of patients with multidrug-resistant tuberculosis. Int J Tuberc Lung Dis [Internet]. 2004 [cited 2020 Jul 22];8(November 2003):749–59. Available from:

For peer review only - http://bmjopen.bmj.com/site/about/guidelines.xhtml

For peer review only

http://docserver.ingentaconnect.com/deliver/connect/iuatld/10273719/v8n6/s11.pdf?expires=1605965794&id=0000&titleid=3764&checksum=BB7C2606D65A6484F704EDA415435129

24. Huq KATME, Moriyama M, Zaman K, et al. Health seeking behaviour and delayed management of tuberculosis patients in rural Bangladesh. 2018;

25. Tola HH, Tol A, Shojaeizadeh D. Tuberculosis Treatment Non-Adherence and Lost to Follow Up among TB Patients with or without HIV in Developing Countries : A Systematic Review. Iran J Public Heal. 2015;44(1).

26. Gelaw SM. Socioeconomic Factors Associated with Knowledge on Tuberculosis among Adults in Ethiopia. Tuberc Res Treat [Internet]. 2016 [cited 2020 Sep 12];2016. Available from: http://downloads.hindawi.com/journals/trt/2016/6207457.pdf

27. Marahatta SB, Yadav RK, Giri D, et al. Barriers in the access, diagnosis and treatment completion for tuberculosis patients in central and western Nepal: A qualitative study among patients, community members and health care workers. PLoS One [Internet]. 2020 [cited 2020 Sep 13];15(1):e0227293. Available from: https://journals.plos.org/plosone/article?id=10.1371/journal.pone.0227293

28. Khanal S, Elsey H, King R, et al. Development of a patient-centred, psychosocial support intervention for multi-drug-resistant tuberculosis (MDR-TB) care in Nepal. PLoS One [Internet]. 2017 [cited 2020 Apr 19];12(1):1–16. Available from: https://journals.plos.org/plosone/article?id=10.1371/journal.pone.0167559

29. Westerlund EE, Tovar MA, Lönnermark E, et al. Tuberculosis-related knowledge is associated with patient outcomes in shantytown residents; Results from a cohort study, Peru. J Infect [Internet]. 2015 [cited 2020 Jul 22];71(3):347–57. Available from: https://www.journalofinfection.com/article/S0163-4453(15)00188-7/fulltext

30. Mahato RK, Laohasiriwong W, Vaeteewootacharn K. Major Delays in the Diagnosis and

For peer review only - http://bmjopen.bmj.com/site/about/guidelines.xhtml

Management of Tuberculosis Patients in Nepal. J Clin Diagnostic Res [Internet]. 2015 [cited 2020 Feb 1];9(10):5–9. Available from: https://www.ncbi.nlm.nih.gov/pmc/articles/PMC4625264/#:~:text=reduce such delays.-,Conclusion,facilities%2C poor knowledge on TB.

31. Thapa B, Chadha SS, Das A, et al. High and equitable tuberculosis awareness coverage in the community-driven Axshya TB control project in India. Public Heal Action [Internet]. 2015 [cited 2020 Jul 22];5(1):70–3. Available from: https://europepmc.org/article/pmc/4525368

32. Ministry of Health, New ERA, ICF. Nepal Demographic and Health Survey 2016 [Internet]. Kathmandu; 2017 [cited 2020 Nov 17]. Available from: https://www.dhsprogram.com/pubs/pdf/fr336/fr336.pdf

33. Aryal S, Pandey S, Bhandari A, et al. Stigma Related to Tuberculosis Among Patients Attending DOTS Clinics of Dharan Municipality. Kathmandu Univ Med J [Internet]. 2012 [cited 2020 Jun 10];27(1):48–52. Available from: http://kumj.com.np/issue/37/48-52.pdf

34. Cremers AL, Laat MM De, Kapata N, et al. Assessing the Consequences of Stigma for Tuberculosis Patients in Urban Zambia. PLoS One [Internet]. 2015 [cited 2020 Apr 19];10(3). Available from: https://pubmed.ncbi.nlm.nih.gov/25806955/

35. Ngamvithayapong-Yanai J, Luangjina S, Thawthong S, et al. Stigma against tuberculosis may hinder non-household contact investigation: a qualitative study in Thailand. Public Heal Action [Internet]. 2019 [cited 2020 Nov 1];9(1):15–23. Available from: https://www.ncbi.nlm.nih.gov/pmc/articles/PMC6436487/pdf/i2220-8372-9-1-15.pdf

36. Munro SA, Lewin SA, Smith HJ, et al. Patient Adherence to Tuberculosis Treatment : A Systematic Review of Qualitative Research. Plos Med [Internet]. 2007 [cited 2020 Aug 6];4(7). Available from: https://journals.plos.org/plosmedicine/article?id=10.1371/journal.pmed.0040238

For peer review only - http://bmjopen.bmj.com/site/about/guidelines.xhtml

37. Stosic M, Vukovic D, Babic D, et al. Risk factors for multidrug-resistant tuberculosis among tuberculosis patients in Serbia : a case-control study. BMC Public Health [Internet]. 2018 [cited 2020 Nov 18];18(1114). Available from: https://bmcpublichealth.biomedcentral.com/articles/10.1186/s12889-018-6021-5

38. Marahatta S, Kaewkungwal J, Ramasoota P, et al. Risk factors of Multidrug Resistant Tuberculosis in central Nepal : A pilot study. Kathmandu Univ Med J [Internet]. 2010 [cited 2020 Oct 20];9(32):392–7. Available from: https://www.nepjol.info/index.php/KUMJ/article/view/6238

39. Craig GM, Daftary A, Engel N, et al. Tuberculosis stigma as a social determinant of health : a systematic mapping review of research in low incidence countries. Int J Infect Dis [Internet]. 2017;56:90–100. Available from: http://dx.doi.org/10.1016/j.ijid.2016.10.011

40. Cremers AL, Laat MM De, Kapata N, et al. Assessing the Consequences of Stigma for Tuberculosis Patients in Urban Zambia. 2015;113:1–16.

41. Munro SA, Lewin SA, Smith HJ, et al. Patient Adherence to Tuberculosis Treatment : A Systematic Review of Qualitative Research. Plos Med. 2007;4(7).

42. Stosic M, Vukovic D, Babic D, et al. Risk factors for multidrug-resistant tuberculosis among tuberculosis patients in Serbia : a case-control study. 2018;1–8.

43. Sb M, Kaewkungwal J, Ramasoota P, et al. Risk factors of Multidrug Resistant Tuberculosis in central Nepal : A pilot study. 2010;9(32):2–7.

44. Uplekar M, Weil D, Lonnroth K, et al. WHO's new end TB strategy. Lancet [Internet]. 2015 [cited 2020 Jul 2];385(9979):1799–801. Available from: https://linkinghub.elsevier.com/retrieve/pii/S0140-6736(15)60570-0

45. Wingfield T, Boccia D, Tovar M, et al. Defining Catastrophic Costs and Comparing Their

For peer review only

Importance for Adverse Tuberculosis Outcome with Multi-Drug Resistance: A Prospective Cohort Study, Peru. PLoS Med. 2014;11(7).

46. Tanimura T, Jaramillo E, Weil D, et al. Financial burden for tuberculosis patients in low-And middle-income countries: A systematic review. Eur Respir J [Internet]. 2014 [cited 2020 Jul 2];43(6):1763–75. Available from: https://erj.ersjournals.com/content/erj/43/6/1763.full.pdf

47. Hoa N, Nhung N. National tuberculosis patients cost survey: research findings lead to change in policy and practice. Viet Nam. Public Heal Action [Internet]. 2019 [cited 2020 Jul 22];9(2):50–2. Available from: https://www.ncbi.nlm.nih.gov/pmc/articles/PMC6645445/

48. Viney K, Islam T, Hoa NB, et al. The Financial Burden of Tuberculosis for Patients in the Western-Pacific Region. Trop Med Infect Dis [Internet]. 2019 [cited 2020 Jul 22];4(2):94. Available from: https://www.ncbi.nlm.nih.gov/pmc/articles/PMC6631110/

49. Pedrazzoli D, Borghi J, Viney K, et al. Measuring the economic burden for TB patients in the end TB strategy and universal health coverage frameworks. Int J Tuberc Lung Dis. 2019;23(1):5–11.

50. Mcintyre D, Thiede M, Whitehead M. What are the economic consequences for households of illness and of paying for health care in low- and middle-income country contexts ? Soc Sci Med [Internet]. 2006 [cited 2020 Apr 19];62:858–65. Available from: https://www.sciencedirect.com/science/article/abs/pii/S0277953605003631?via%3Dihub

51. Mishra P, Hansen EH, Sabroe S, et al. Socio-economic status and adherence to tuberculosis treatment: a case-control study in a district of Nepal. Int J Tuberc Lung Dis [Internet]. 2005 [cited 2020 Jan 28];9(10):1134–9. Available from: http://docserver.ingentaconnect.com/deliver/connect/iuatld/10273719/v9n10/s11.pdf?expires=1605975237&id=0000&titleid=3764&checksum=7F8B03920562B289941E5A89AAD

For peer review only - http://bmjopen.bmj.com/site/about/guidelines.xhtml

1F81A

52. Laohasiriwong W, Mahato RK, Koju R, et al. Delay for First Consultation and Its Associated Factors among New Pulmonary Tuberculosis Patients of Central Nepal. Tuberc Res Treat [Internet]. 2016;2016:4583871. Available from: http://downloads.hindawi.com/journals/trt/2016/4583871.pdf

53. Zegeye A, Dessie G, Wagnew F, et al. Prevalence and determinants of anti-tuberculosis treatment non-adherence in Ethiopia: A systematic review and meta-analysis. PLoS One [Internet]. 2019 [cited 2020 Sep 13];14(1):e0210422. Available from: https://journals.plos.org/plosone/article/file?id=10.1371/journal.pone.0210422&type=printable

54. Ha A, Bijlsma MW, Malla P, et al. The road to tuberculosis treatment in rural Nepal : A qualitative assessment of 26 journeys. BMC Health Serv Res. 2008;10:1–10.

55. Arinaminpathy N, Batra D, Khaparde S, et al. The number of privately treated tuberculosis cases in India: an estimation from drug sales data. Lancet Infect Dis [Internet]. 2016 [cited 2020 Jul 22];16(11):1255–60. Available from: https://www.thelancet.com/action/showPdf?pii=S1473-3099%2816%2930259-6

56. Joloba M, Mwangi C, Alexander H, et al. Strengthening the Tuberculosis Specimen Referral Network in Uganda: The Role of Public-Private Partnerships. J Infect Dis [Internet]. 2016 [cited 2020 Nov 1];213(S2):S41–6. Available from: https://www.ncbi.nlm.nih.gov/pmc/articles/PMC4914746/

57. Subbaraman R, Nathavitharana RR, Satyanarayana S, et al. The Tuberculosis Cascade of Care in India's Public Sector: A Systematic Review and Meta-analysis. PLoS Med [Internet]. 2016 [cited 2020 Jul 22];13(10):1–38. Available from: https://journals.plos.org/plosmedicine/article/file?id=10.1371/journal.pmed.1002149&type=printable

For peer review only - http://bmjopen.bmj.com/site/about/guidelines.xhtml

58. Storla DG, Yimer S, Bjune GA. A systematic review of delay in the diagnosis and treatment of tuberculosis. BMC Public Health [Internet]. 2008 [cited 2020 Jul 22];8:1–9. Available from: https://bmcpublichealth.biomedcentral.com/articles/10.1186/1471-2458-8-15

59. Buregyeya E, Criel B, Nuwaha F, et al. Delays in diagnosis and treatment of pulmonary tuberculosis in Wakiso and Mukono districts, Uganda. BMC Public Health [Internet]. 2014 [cited 2020 Jul 22];14:586. Available from: https://bmcpublichealth.biomedcentral.com/articles/10.1186/1471-2458-14-586

60. Kamineni VV, Wilson N, Das A, et al. Addressing poverty through disease control programmes: Examples from Tuberculosis control in India. Int J Equity Health [Internet]. 2012 [cited 2020 Jul 22];11(1). Available from: https://equityhealthj.biomedcentral.com/articles/10.1186/1475-9276-11-17

61. Baral SC, Aryal Y, Bhattrai R, et al. The importance of providing counselling and financial support to patients receiving treatment for multi-drug resistant TB: mixed method qualitative and pilot intervention studies [Internet]. 2014. Available from: http://www.biomedcentral.com/1471-2458/14/46

62. Carter DJ, Glaziou P, Lönnroth K, et al. The impact of social protection and poverty elimination on global tuberculosis incidence: a statistical modelling analysis of Sustainable Development Goal 1. Lancet Glob Heal. 2018;6(5):e514–22.

63. Oliosi JGN, Reis-Santos B, Locatelli RL, et al. Effect of the Bolsa Familia Programme on the outcome of tuberculosis treatment: a prospective cohort study. Lancet Glob Heal. 2018;219–26.

64. Klein K, Bernachea MP, Id SI, et al. Evaluation of a social protection policy on tuberculosis treatment outcomes : A prospective cohort study. PLoS Med. 2019;9:1–16.

65. Gebremariam MK, Bjune GA, Frich JC. Barriers and facilitators of adherence to TB

For peer review only - http://bmjopen.bmj.com/site/about/guidelines.xhtml

treatment in patients on concomitant TB and HIV treatment: A qualitative study. BMC Public Health. 2010;10.

66. Vries SG de, Cremers AL, Heuvelings CC, et al. Barriers and facilitators to the uptake of tuberculosis diagnostic and treatment services by hard-to-reach populations in countries of low and medium tuberculosis incidence: a systematic review of qualitative literature. Lancet Infect Dis. 2017;17(5):e128–43.

67. Wingfield T, Boccia D, Tovar MA, et al. Designing and implementing a socioeconomic intervention to enhance TB control : operational evidence from the CRESIPT project in Peru. 2015;15.

68. Wingfield T, Tovar MA, Huff D, et al. The economic effects of supporting tuberculosis-affected households in Peru. Eur Respir J. 2016;48:1396–410.

For peer review only

Reference (From table 1)

Table 1

[21] Weaver MS, Lönnroth K, Howard SC, et al. Interventions to improve adherence to treatment for paediatric tuberculosis in low- and middle-income countries: a systematic review and meta-analysis. Bull World Health Organ [Internet]. 2015 [cited 2020 Jul 29];93(10):700-711B. Available from: https://www.ncbi.nlm.nih.gov/pmc/articles/PMC4645428/

LEGEND

Figure 1: Data for population numbers and TB case notification rate is taken from the National TB Control Center Annual Report, 2018.

Figure 2: The inner white circle contains the key categories that influence TB service access and engagement, which are adapted from a World Health Organization Medication Adherence Framework (see Methods).[21] The middle red circle indicates the main barriers identified for each category, which may threaten access to TB services. The outer green circle indicates the main facilitators (current or potential) for each category, which may enhance access to TB services. Barriers relating to "TB, health, and basic education", "Social protection and nutrition", and "Psychosocial" were perceived by the project team to be modifiable by a household level socioeconomic intervention. Barriers relating to the "Health System" were perceived by the project team to be non-modifiable by a household level socioeconomic intervention and are, therefore, separated from the other categories and represented by dotted lines. "PPM" as a health system barrier refers to the protracted and convoluted patient journey through public and private healthcare providers, which was reported as being associated with increased economic impact, especially related to out-of-pocket costs. The surrounding bidirectional arrows indicate the cross-FGD finding that adequate funding and advocacy, and political will and commitment were perceived as vital structural factors to enable the facilitators identified to overcome the barriers identified. **Abbreviations** TB: Tuberculosis; NTP: National Tuberculosis Program; DS-TB: Drug-sensitive Tuberculosis; DOTS: Directly Observed Treatment Shortcourse; PPM: Public-Private Mix. **Reference:** [21] Weaver MS, Lönnroth K, Howard SC, et al. Interventions to improve adherence to treatment for paediatric tuberculosis in low- and middle-income countries: a systematic review and meta-analysis. Bull World Health Organ [Internet]. 2015 [cited 2020 Jul 29];93(10):700-711B. Available from: https://www.ncbi.nlm.nih.gov/pmc/articles/PMC4645428/

For peer review only - http://bmjopen.bmj.com/site/about/guidelines.xhtml

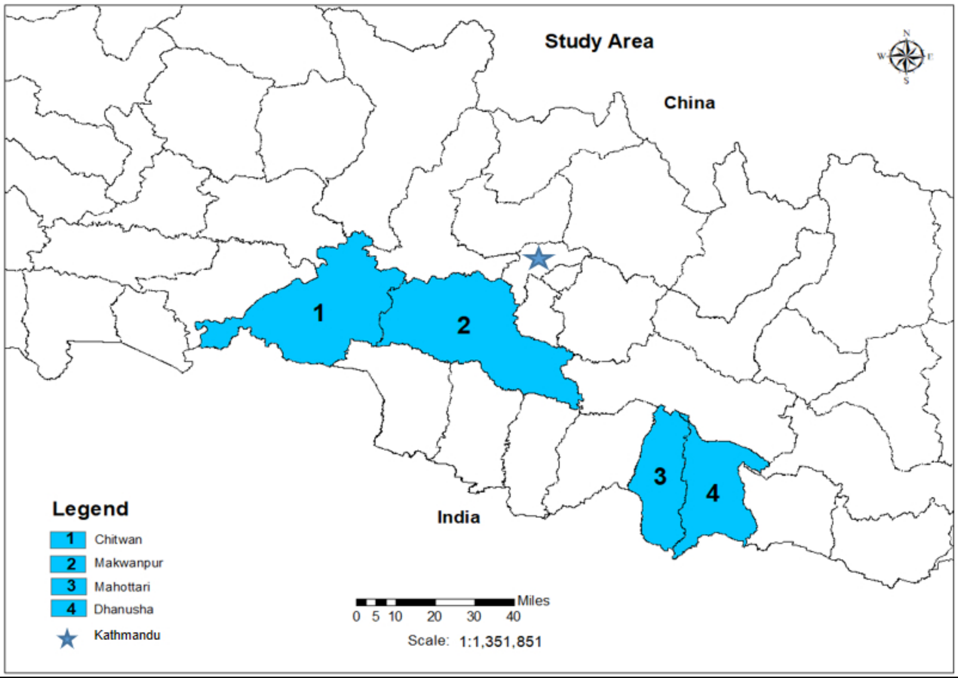

District	Population	Case Notification Rate (per 100,000 population)
Makwanpur	480,000	147
Chitwan	585,000	158
Dhanusha	823,000	100
Mahottari	681,000	131

Data for population numbers and TB case notification rate is taken from the National TB Control Center Annual Report, 2018.

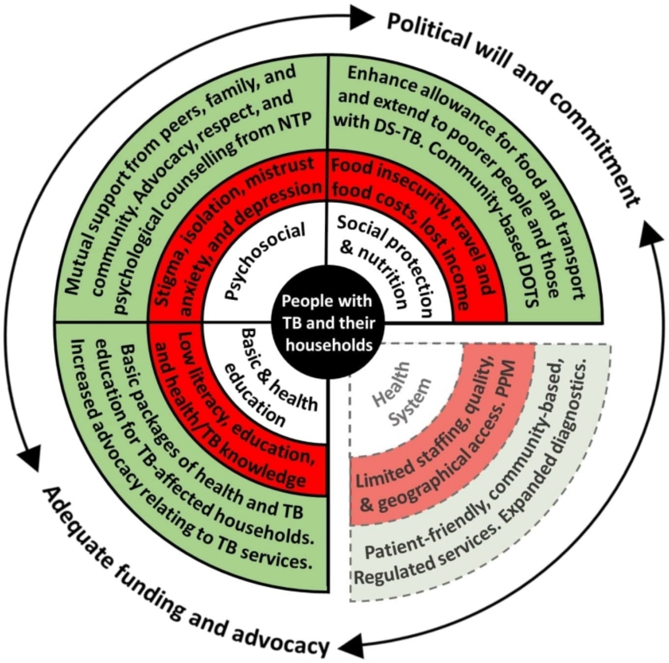

The inner white circle contains the key categories that influence TB service access and engagement, which are adapted from a World Health Organization Medication Adherence Framework (see Methods).[21] The middle red circle indicates the main barriers identified for each category, which may threaten access to TB services. The outer green circle indicates the main facilitators (current or potential) for each category, which may enhance access to TB services. Barriers relating to "TB, health, and basic education", "Social protection and nutrition", and "Psychosocial" were perceived by the project team to be modifiable by a household level socioeconomic intervention. Barriers relating to the "Health System" were perceived by the project team to be non-modifiable by a household level socioeconomic intervention and are, therefore, separated from the other categories and represented by dotted lines. "PPM" as a health system barrier refers to the protracted and convoluted patient journey through public and private healthcare providers, which was reported as being associated with increased economic impact, especially related to out-of-pocket costs. The surrounding bidirectional arrows indicate the cross-FGD finding that adequate funding and advocacy, and political will and commitment were perceived as vital structural factors to enable the facilitators identified to overcome the barriers identified. Abbreviations TB: Tuberculosis; NTP: National Tuberculosis Program; DS-TB: Drug-sensitive Tuberculosis; DOTS: Directly Observed Treatment Shortcourse; PPM: Public-Private Mix. Reference: 21 Weaver MS, Lönnroth K, Howard SC, et al. Interventions to improve adherence to treatment for paediatric tuberculosis in low- and middle-income countries: a systematic review and meta-analysis. Bull World Health Organ [Internet]. 2015 [cited 2020 Jul 29];93(10):700-711B. Available from: https://www.ncbi.nlm.nih.gov/pmc/articles/PMC4645428

537x506mm (96 x 96 DPI)

Supplementary File II: Detailed list of FGD stakeholder groups and participants

Date	Stakeholder group	Gender	Age	District	Total number of participants
17-Aug-18	People diagnosed with TB, mixed sex group	Female	18	Makwanpur	7
		Male	25	Mahottari	
		Male	46	Makwanpur	
		Male	20	Dhanusha	
		Female	19	Makwanpur	
		Male	32	Chitwan	
		Male	56	Chitwan	
1-Feb-19	People diagnosed with MDR-TB	Male	44	Chitwan	7
		Male	70	Chitwan	
		Male	24	Chitwan	
		Male	24	Chitwan	
		Male	48	Chitwan	
		Male	47	Chitwan	
		Female	24	Chitwan	
1-Apr-19	Females diagnosed with TB	Female	60	Mahottari	7
		Female	25	Makwanpur	
		Female	40	Mahottari	
		Female	45	Chitwan	
		Female	45	Dhanusha	
		Female	25	Dhanusha	
		Female	26	Makwanpur	
20-Sep-18	Community Leaders	Female	51	Chitwan	6
		Male	48	Mahottari	
		Male	39	Makwanpur	
		Male	49	Chitwan	
		Female	52	Chitwan	
		Male	43	Dhanusha	

For peer review only

22-Sep-18	Civil Society Organization	Male	39	Chitwan	7
		Male	44	Chitwan	
		Male	66	Chitwan	
		Male	26	Mahottari	
		Male	47	Chitwan	
		Male	45	Makwanpur	
		Male	28	Dhanusha	
28-Sep-18	TB Healthcare Professionals	Male	55	Kathmandu	12
		Male	30	Kathmandu	
		Male	34	Kathmandu	
		Male	33	Kathmandu	
		Female	28	Kathmandu	
		Male	55	Kathmandu	
		Male	45	Kathmandu	
		Male	47	Kathmandu	
		Male	57	Kathmandu	
		Male	57	Kathmandu	
		Male	55	Kathmandu	
		Male	49	Kathmandu	
5-Jun-19	Community Mobilizers	Male	47	Dhanusha	8
		Female	30	Chitwan	
		Female	32	Makwanpur	
		Male	25	Dhanusha	
		Male	30	Chitwan	
		Male	40	Mahottari	
		Male	26	Mahottari	
		Female	20	Makwanpur	
**Total participants: 54 (38 male, 16 female)**					

For peer review only

Supplementary File III: Healthcare system, TB diagnosis and care delivery barriers and facilitators

During thematic analysis, it was noted that the FGD participants identified "higher level" barriers and facilitators to accessing and engaging with TB services including governmental policy, political commitment, public-private mix, and healthcare infrastructure. that would not be directly modifiable through a socioeconomic support intervention for TB-affected households. While important and informative, the research team felt that such themes could not practically be addressed by – and hence would not directly inform the design of - a discrete socioeconomic support package for TB-affected households for future trial evaluation. Here, the perceived higher level barriers and facilitators are mapped to Category IV "Healthcare system, TB diagnosis and care delivery" of the adapted WHO treatment adherence framework.[1] These barriers and facilitators are summarized in Figure 2 of the main article and expanded on in more detail below.

Theme: Healthcare system, TB diagnosis and care delivery barriers

Across FGDs, participants reported limited staffing at health centers, poor geographical access, unsatisfactory quality of the TB services and multiple visits in public and private health centers for diagnosis and treatment as the major barriers to accessing and engaging with TB treatment. Participants mentioned that the incorrect diagnosis of TB caused long and convoluted journey during the diagnosis and treatment, including a public-private (PPM) mix of hospitals and clinics and pharmacies before reaching NTP diagnostic and treatment services:

> **FGD with female diagnosed with TB, 25-year-old female:** *"For many times, I went to hospitals and bought medicines. My health was worsening. A doctor prescribed medicines for increased blood pressure, I had those medicines. Also, later another doctor prescribed me medicines for gastritis. I had those medicines as well. None of the medicines made me feel better. It took a long time until I was diagnosed [of TB] in a government hospital."*

The NTP stakeholders mentioned that the pharmacy services were perceived to be compromised because of the practice of prescribing antibiotics without suitable clinical or microbiological confirmation of infection. Moreover, the practice of changing to new and more expensive antibiotics was noted if the disease wasn't cured. However, such services were noted to be thriving and supported a culture of, as one NTP stakeholder noted, *"medicine dependence in South Asia"*.

> **FGD with NTP stakeholders, 55-year-old male:** *"If I have a cough, the local pharmacy will give me amoxicillin. [If I continue to cough] I might get a more expensive antibiotic like cefixime. If I tell the pharmacist that I am coughing up blood - a clear sign in a place like Nepal that this could be TB - I might get some bigger, more expensive antibiotics but the thought would not be about tuberculosis. The pharmacist should tell me to get checked for TB but instead I will get sold antibiotics. There are thousands of pharmacies [like this] in South Asia."*

Participants from the FGD with NTP stakeholders raised concerns about the risk of such antibiotic practices driving increasing rates of drug-resistant TB (DR-TB) and multidrug-resistant TB (MDR-

For peer review only

TB) in Nepal. The practice of seeking private care initially was felt to be commonplace among those of higher socioeconomic position because of sufficient household resources coupled with a perception of private services being quicker and/or higher quality than public services and more likely to help them to become cured. Nevertheless, poorer households were reported to visit cheaper, independent private healthcare providers, some of whom were reported to have no or limited formal training, in their local village. Ultimately, it was reported across FGDs that the decision to seek private care and the level of quality of private care provided was dictated by money.

> **FGD with civil society organization, 48 years old male:** *"The majority of the patients go to medical [pharmacy]. If they have to go to district [larger referral hospital], then the expenses will be higher. If they attend health centres, the expense depends on the distance."*

Geographical barriers such as long distances to reach health facilities, weather conditions such as rain, natural disasters (landslides and flooding), difficult roads and limited transportation options were discussed in depth across FGDs. These barriers were perceived as hampering access to TB diagnosis, impeding engagement with TB treatment and DOTS, and had a knock-on impact on access to broader health services.

The participants in FGDs with community stakeholders and people with TB perceived restricted opening hours, delayed opening, limited time available for clinical investigation [in hospitals], and prolonged waiting times for consultation and receiving reports as barriers to both TB diagnosis and treatment. Community stakeholders reported that this was especially important for labourers given the competing demands to earn money during peak labour hours or seek healthcare during clinic opening hours. These barriers were compounded by issues relating to staff lateness and staff shortages or absence at healthcare centers that provided TB services:

> **FGD with people diagnosed with TB, 25-year-old male:** "*There are no health workers. It's such a small hospital [where I attend for TB medicine], there are five employees but four of them don't work. When I go to find and take my medicines, they say they don't know and ask me to look for it myself. The main doctor is in a meeting all the time, he doesn't know."*

Moreover, participants perceived that there was negative behaviour of healthcare workers towards people seeking TB diagnosis or who were taking TB treatment as a notable barrier and disincentive to attend. There was a perception that some NTP staff at health centers took little interest in people with TB. A participant with TB disease mentioned that the healthcare workers at the DOTS center only provide medicines and put a tick mark in the treatment card or sometimes even proper recording is missed in the card. Another participant with TB also shared his experience during his treatment where he was often told to take medicines from the shelf at the DOTS center. There were also reports of discourteous consultations

During FGDs with people with MDR-TB, room conditions, atmosphere, and quality of TB services at MDR-TB centers were reported as not being patient-friendly and a significant barrier to engagement. Apart from waiting times, issues raised included crowded waiting rooms and inadequacies in cleanliness and hygiene of clinics and hospitals. Although people with MDR-TB

For peer review only

reported supportive care and staff at the DR-TB hostels in which some were managed during their treatment, others reported that the facilities were not as expected. One participant describing their room to have been *"colder than snow"* and another shared that the substandard conditions of the hostel rooms made him feel even worse and so he did not remain there. The perception of lack of patient friendly services was also acknowledged as a problem in the FGDs with NTP stakeholders and people with TB:

Theme: Healthcare system, TB diagnosis and care delivery facilitators

During the FGD with NTP stakeholders, PPM was discussed in detail and, specifically, integration of the public and private sector TB services as a potential facilitator. Both NTP stakeholders and people with TB cited that government hospitals should mandate pharmacies and private providers to refer people with TB to government services. However, it was noted that current legislative powers were insufficient to realise this:

> **FGD with NTP stakeholders, 33-year-old male:** *"We can regulate the health professionals but we cannot regulate the pharmacies….where the majority of TB cases are going and taking pills."*

It was noted across FGDs that outreach and decentralized community-based services that incorporate use of advanced diagnostic tools would be beneficial to reach healthcare facilities. NTP stakeholders reported that decentralization of GeneXpert had revolutionized TB diagnosis in the hills of Nepal, increasing TB case detection while promptly identifying people with DR-TB and MDR-TB. The NTP stakeholders mandated roll out of GeneXpert across Nepal and suggested integration of GeneXpert with targeted active-case finding strategies such as TB camps and door-to-door campaigns. The participants also suggested community screening, which focused on reaching underserved, vulnerable people at high risk of TB, was appropriate. People with MDR-TB reported that such active case finding strategies had been well received:

> **FGD with people with MDR-TB, 44-year-old male:** *"What I like the most is they come to our houses to collect sputum."*

In addition to reports of negative behaviour from NTP staff during treatment, participants with TB also reported experiences of positive behaviour towards them as a motivating factor, which had encouraged them to take their medicines regularly. Supportive behaviour coupled with education about medication – including side effects, importance of adherence, and duration - during initial consultations was seen as an enabler for treatment engagement and adherence. During the FGD with NTP stakeholders, it was mentioned that providing adequate time to patients, proper counseling, and consultation enable people with TB disease to complete their treatment and get cured. This was perceived to be enhanced by community outreach activities by community mobilisers and female community health volunteers to ensure patients had the best opportunity to achieve treatment success:

> **FGD with female with TB, 60 years old female:** *"The doctor treated me like a normal patient, cared for me, asked how I was feeling and gave me medicine."*

For peer review only

Furthermore, during the FGD with people with TB, participants mentioned the necessity of patient-centric treatment services such as taking TB-medications at home instead of daily visits to the DOTS centers, which is often time consuming.

FGD with female diagnosed with TB, 45-year-old female: *"It's good to bring medicine at home. Sometimes, we have work at the time when we have to go to take medicines."*

References

1. Weaver MS, Lönnroth K, Howard SC, Roter DL, Lam CG. Interventions to improve adherence to treatment for paediatric tuberculosis in low- and middle-income countries: a systematic review and meta-analysis. Bull World Health Organ. 2015;93(10):700-711B.

For peer review only

Supplementary File IV: Focus group discussion Coding Framework

Category 1: TB, health, and basic education

**TB, health, and basic education barriers**				
**FGD**	**First order category**	**Second order themes**	**Third order themes**	**Example Quote**
People diagnosed with TB	TB, health, and basic education	Inadequate knowledge on TB services	Information barrier to access	**FGD with people diagnosed with TB, 18-year-old female:** *"I didn't know that medicines were free in hospitals. I knew it only when I visited health post."*
People diagnosed with MDR-TB	TB, health, and basic education	Limited knowledge on TB disease	Information barrier to access	**FGD with people diagnosed with MDR-TB, 70-year-old male:** *"No one told me anything [about TB] but I knew that it was a communicable disease."*
Community leaders	TB, health, and basic education	Inadequate advocacy on TB services	Information barrier to access	**FGD with community leaders, 45-year-old male:** *"There is no person to advocate about [available health and TB] services."*
People diagnosed with MDR-TB	TB, health, and basic education	Limited knowledge on TB disease and TB medicines dosages	Information barrier to adhere to TB services	**FGD with people diagnosed with MDR-TB, 47-year-old male:** *"I took medicine for 2 months and then left for 15 days because I thought I was feeling better."*
Civil society organization	TB, health, and basic education	Lack of awareness raising activities particularly in poorer and socially marginalized	Information barrier to access	**FGD with civil society organization, 48-year-old male:** "*In addition, programmatic TB awareness-raising activities were seen as scarce, especially among poorer, "educationally and socially marginalized."*

For peer review only

Civil society organization	TB, health, and basic education	Inadequate awareness raising educational materials	Information barrier to access	**FGD with civil society organization, 49-year-old male: "***There has been few educational materials development for TB awareness at present in comparison to the past. Therefore, people do not go to the health facility."*
**TB, health, and basic education facilitators**				
**FGD**	TB, health, and basic education	**Second order theme**	**Third order theme**	**Quotes**
Civil society organization	TB, health, and basic education	Improvements in basic education on TB	Education on TB disease and advocacy about its services	**FGD with civil society organization, 48-year-old male***: "TB education should be spread intensively to village households."*
People diagnosed with TB	TB, health, and basic education	Awareness about TB disease, treatment and prevention	Education on TB disease and advocacy about its services	**FGD with people diagnosed with TB, 32-year-old male:** *The government should develop TB awareness raising programs….[including] in the media, to tell people about TB symptoms, check-ups….. and that it's a curable disease."*
People diagnosed with TB	TB, health, and basic education	Community engagement<br><br>Mobilization of TB champions and peers	Education on TB disease and advocacy about its services	**FGD with people diagnosed with TB, 32-year-old male:** *"People previously affected by TB could tell their story to community and mother's groups. I am 100% sure this will work."*

People diagnosed with TB	TB, health, and basic education	Awareness about TB<br><br>Information on TB medications	Education on TB disease and advocacy about its services	**FGD with people diagnosed with TB, 46-year-old male:** *"People in the villages will take medicines regularly if they are educated….the government should strengthen education on TB medication."*
Civil society organization	TB, health, and basic education	Community engagement<br><br>Mobilization of community leaders for TB awareness	Education on TB disease and advocacy about its services	**FGD with civil society organization, 52-year-old female:** *"We need to create [educational] groups attached to health centres and schools….Community and locally-elected leaders and teachers could give education to their communities and conduct TB awareness training and workshops."*
TB healthcare professional	TB, health, and basic education	Awareness about TB<br><br>Communication to influence health beliefs and change behaviour	Education on TB disease and advocacy about TB services	**FGD with TB healthcare professional, 55-year-old male:** *"Changing [people's] attitude [about TB] is one of the things we can start doing to….. make a change in [people's] behaviour."*
Community Mobilizers	TB, health, and basic education	Awareness about TB<br><br>Improved communication to influence health beliefs and change behaviour	Education on TB disease and advocacy about TB services	**FGD with Community Mobilizers, 30-year-old male:** *"[Some] patients don't believe us when we tell them that they have been diagnosed with TB. They say they'll go to India for check-up. In our region, people who believe in a certain religion think that TB will cure itself."*

For peer review only

People diagnosed with TB	TB, health, and basic education	Advocacy on TB services	Expansion of TB services in community	**FGD with people diagnosed with TB, 32-year-old male:** *"Health facilities should conduct door to door campaign. [Health facility] should collect sputum in a timely manner. That's it, actually there is a lack of advocacy."*
Community leaders	TB, health, and basic education	Prioritize underserved people	Education on TB disease and advocacy about TB services in remote communities	**FGD with community leaders, 66-year-old male:** *"One of the reasons is that many people do not have adequate information because in this era radio, television, mic, seminars, workshops are organized where people/ community are literate. But those people who do not have access to any of these media are disadvantaged from these programs. So, we should focus more to those people as well."*

Category 2: Social protection and nutrition

**Social protection, nutrition, and economic barriers**				
**FGD**	**First order categories**	**Second order themes**	**Third order themes**	**Quote**
People diagnosed with TB	Social protection, nutrition, and economic	Out-of-pocket expenses for transportation to engage with treatment	Economic barrier to treatment adherence	**FGD with people diagnosed with TB, 56-year-old male**: *"When I don't have money, I cannot ride auto [local vehicle] because it costs Rs 300 [$2.5 USD] to reach home or clinic. I can't earn three pennies! How can I pay Rs 300 to go to home?"*

For peer review only

People diagnosed with TB	Social Protection, nutrition and economic	Out-of-pocket expenses for diagnostic tests	Economic barrier to access	**FGD with people diagnosed with TB, 46-year-old male:** *"I visited all the pharmacies in my city, about 15-17 medicals [pharmacies] overall. I used to buy pneumonia medicine and take it every time. TB was not initially identified. After a month without taking any medicines, TB was identified [at the government clinic]….. The barrier is more financial. I spent approximately 1.7 lakhs [~1530 USD] for my treatment."*
Civil society organization	Social Protection, nutrition and economic	Out-of-pocket expenses for transportation and nutrition	Economic barrier to treatment adherence	**FGD with civil society organization, 52-year-old female:** *"TB medicines are free but people also need costs for two-way transportation, food. TB illness [and even] TB treatment can make people weak and nutrition is needed. How can people afford [nutrition and transport costs]?"*
People diagnosed with MDR-TB	Social Protection, nutrition and economic	Lost income<br><br>Reduced productivity related to TB illness<br><br>Psychological stress	Economic barrier to treatment adherence	**FGD with people diagnosed with MDR-TB, 24-year-old male:** *I used to be a conductor on a bus but I can't do any work now. I suffer headaches just going to the kitchen to cook rice. How can such a person work?"*
People diagnosed with MDR-TB	Social Protection, nutrition and economic	Lost income<br><br>Reduced productivity related to TB illness	Economic barrier to treatment adherence	**FGD with people diagnosed with MDR-TB, 44-year-old male:** *"I used to work outside but after being affected by MDR-TB, I stopped going to work. I didn't earn money"*

For peer review only

People diagnosed with TB	Social Protection, nutrition and economic	Out-of-pocket expenses for accessing TB services<br><br>Borrowing money as coping strategies	Economic barrier to access and adhere to TB treatment	**FGD with people diagnosed with TB, 25-year-old male:** *"I had difficulties [to pay money to access TB services] and wasn't able to go [to the clinic] for a month….I had to borrow money from my friends."*
People diagnosed with TB	Social Protection, nutrition and economic	Out-of-pocket expenses for accessing TB services<br><br>Selling assets as coping strategies	Economic barrier to treatment adherence	**FGD with people diagnosed with TB, 20-year-old male:** *"I can't pay back the loan [I took out during treatment], which causes problems…..we might have to sell assets to pay back the loan."*
People diagnosed with MDR-TB	Social Protection, nutrition and economic	Inadequate financial support	Economic barrier to treatment adherence	**FGD with people diagnosed with MDR-TB, 24-year-old male:** *"The TB hostel where I am staying assured us that they would give money but they still haven't [up to 20 months into treatment]."*
**Social protection and nutrition**				
**FGD**	**First order categories**	**Second order themes**	**Third order themes**	**Quote**
Civil society organization	Social Protection, nutrition and economic	Provision of nutrition or cash incentives	Nutrition and or economic support to treatment adherence	**FGD with civil society organization, 52-year-old female:** "*When patients take medicine, they will feel weak but they will not have the facility of proper nutritious food. Thus, if certain amount of money is made available to them, then it will help them."*
Community leaders	Social Protection,	Delivery mechanism and timing of incentives	Economic support to treatment adherence	**FGD with community leaders, 45-year-old male:** *"The question is when to give the allowance. It would*

For peer review only

	nutrition and economic			*be better in the first phase [of treatment] because it is [most] valuable at the time one most needs it."*
People diagnosed with TB	Social Protection, nutrition and economic	Provision of economic and nutrition support<br><br>Prioritization of nutrition and economic support	Nutrition and or economic support to treatment adherence	**FGD with people diagnosed with TB, 32-year-old male:** *"Government should provide nutritious food based on the economic status of patients……drug-sensitive patients should also be provided with an allowance based on their socio-economic condition."*

Category 3: Psychosocial barriers

FGD	First order categories	Second order themes	Third order themes	Quote
Female: diagnosed with TB	Psychosocial category	Stigma<br><br>Enacted stigma in the community<br><br>Perceived stigma<br><br>Lack of knowledge	Social barrier to adhere during treatment	**FGD with female: diagnosed with TB, 26-year-old female:** *"I have not told anyone in my family about my disease because they will hate me. I fear that if my family or friends know about my disease, they'll start talking behind my back and not come near me."*
Civil society organization	Psychosocial category	Stigma<br><br>Enacted stigma in the community<br><br>Perceived stigma<br><br>Lack of knowledge	Social barrier to access and adhere during treatment	**FGD with civil society organization, 49-year-old male:** *"People don't want to sit close to or make contact with someone with TB. They can act scared when they're near a patient with TB. Ironically, the same society promotes TB treatment and encourages people with TB to get themselves tested and treated."*

For peer review only

People diagnosed with TB	Psychosocial category	Stigma<br><br>Enacted stigma in the community<br><br>Perceived stigma<br><br>Lack of knowledge	Social barrier to access	**FGD with people diagnosed with TB, 32-year-old male:** *"People may know they have symptoms of TB but are too ashamed to go to the healthposts. People can't say out loud that they have TB. TB is regarded as a big disease and people get criticized for having it. The community perceives a TB patient differently than a normal person due to lack of awareness. That's why it's difficult to end TB."*
Healthcare professional	Psychosocial category	Stigma<br><br>Enacted stigma in the community<br><br>Perceived stigma<br><br>Lack of knowledge<br><br>Limited social support	Social barrier to adhere to treatment	**FGD with TB healthcare professional, 30-year-old male:** *"Some people have very low social support, including from their family. Those people are at risk of not taking a full course of TB treatment."*
People diagnosed with MDR-TB	Psychosocial category	Psychological impact<br><br>Stress, anxiety, and depression	Psychological barrier to treatment adherence	**FGD with people diagnosed with MDR-TB, 48-year-old male:** *"After seeing the healthcare workers, I felt angry and depressed. I didn't want to stay with others, not even with my family. I just sat alone."*
People diagnosed with MDR-TB	Psychosocial category	Psychological impact<br><br>Stress, anxiety, and depression	Psychological barrier to treatment adherence	**FGD with people diagnosed with MDR-TB, 24-year-old male:** *I felt depressed at first. No one in my clan had suffered from this disease. I wondered how I'd got affected."*

For peer review only

People diagnosed with MDR-TB	Psychosocial category	Psychological impact<br><br>Stress, anxiety, and depression	Psychological barrier to treatment adherence	**FGD with people diagnosed with MDR-TB, 47-year-old male:** *"I wanted to die. One of my friends [with MDR-TB] committed suicide after 16 months [of treatment]."*
**Category 3b: Psychosocial facilitators**				
**FGD**	**First order categories**	**Second order themes**	**Third order themes**	**Quote**
emale: diagnosed with TB	Psychosocial category	Family care and support	Mutual support as facilitator to treatment	**FGD with female: diagnosed with TB, 40-year-old female:** *"When I restarted taking medicines, it was very difficult for me. My husband helped me at that time and, slowly, I started getting better."*
People diagnosed with MDR-TB	Psychosocial category	Peer support and motivation	Mutual or social support as facilitator to treatment	**FGD with people diagnosed with MDR-TB, 44-year-old male:** *"My friends and the people in my village told me TB is normal disease and encouraged me that if I took my medicine, I'd be alright."*

For peer review only

Category 4: Healthcare system, TB diagnosis and care delivery

**Category 4a: Healthcare system, TB diagnosis and care delivery barriers**				
**FGD**	**First order categories**	**Second order categories**	**Third order categories**	**Quote**
TB healthcare professional	Healthcare system, TB diagnosis and care delivery	Delay in diagnosis<br><br>Self-perception of disease and choice of treatment<br><br>Repeated visits for diagnosis<br><br>Inadequate referral mechanisms	Convoluted pathway to diagnosis as barrier to access	**FGD with TB healthcare professional, 47-year-old male:** *"When people feel sick [with TB symptoms], they start by using herbal medication at home. When they do not get relief from that medication, they go to Dhami/Jhakri [traditional healers], then the pharmacy, then to a government clinic. So, from the start of their illness to getting the right treatment, is around 1-2 months."*
TB healthcare professional	Healthcare system, TB diagnosis and care delivery	Inadequate treatment regulation<br><br>Under-recognition of TB symptoms<br><br>Inadequate referral mechanisms	Unregulated policy for medicines distribution	**FGD with TB healthcare professional, 55-year-old male:** *"If I have a cough, the local pharmacy will give me amoxicillin. [If I continue to cough] I might get a more expensive antibiotic like cefixime. If I tell the pharmacist that I am coughing up blood - a clear sign in a place like Nepal that this could be TB - I might get some bigger, more expensive antibiotics but the thought would not be about tuberculosis. The pharmacist should tell me to get checked for TB but instead I will get sold antibiotics. There are thousands of pharmacies [like this] in South Asia."*

For peer review only

Community Mobilizers	Healthcare system, TB diagnosis and care delivery	Geographical inaccessibility to reach TB centers<br><br>Natural disasters<br><br>Centralised location of health facilities	Geographical barriers to access TB diagnosis and care	**FGD with Community Mobilizers, 32-year-old female:** *"In rainy season, flooding makes it difficult for patients to go and get medicine and [health posts] are inaccessible"*
Community Mobilizers	Healthcare system, TB diagnosis and care delivery	Geographical inaccessibility to reach TB centers<br><br>Poor quality of road and transportation network	Geographical barriers to access TB diagnosis and care	**FGD with Community Mobilizers, 20-year-old male:** *"One of the main reasons for not taking medicine is because the health posts are far away….and difficult to reach….especially when the rivers get bigger and wider; they can sweep people away. If the health post was nearer, it would be easier."*
Civil society organization	Healthcare system, TB diagnosis and care delivery	Poor healthcare services<br><br>Limited or inconvenient opening times of health facilities	Inadequate services from health centers as barrier to adhere to treatment	**FGD with civil society organization, 52-year-old female:** *"If we don't go on time, we don't meet the doctor. The patients must reach the hospital clinic before 11 and take a ticket.*
Community leaders	Healthcare system, TB diagnosis and care delivery	Geographical inaccessibility to reach TB centers<br><br>Poor quality of road and transportation network	Geographical barriers to access TB diagnosis and care	**FGD with community leaders, 39-year-old male:** *"The health facilities are far and also may be the difficult road."*

For peer review only

Community leaders	Healthcare system, TB diagnosis and care delivery	Geographical inaccessibility to reach TB centers	Geographical barriers to access TB diagnosis and care	**FGD with community leaders, 47-year-old male:** *"They are far from the access of Health facilities. Due to this reason they do not get medicine regularly."*
People diagnosed with TB	Healthcare system, TB diagnosis and care delivery	Poor healthcare services<br>Limited human resources	Inadequate services from health centers as barrier to adhere to treatment	**FGD with people diagnosed with TB, 25-year-old male:** "*There are no health workers. It's such a small hospital [where I attend for TB medicine], there are five employees but four of them don't work. When I go to find and take my medicines, they say they don't know and ask me to look for it myself. The main doctor is in a meeting all the time, he doesn't know."*
TB healthcare professional	Healthcare system, TB diagnosis and care delivery	Poor healthcare services	Inadequate patient centric care as barrier to treatment adherence	**FGD with TB healthcare professional, 55-year-old male:** *"If we try to put ourselves in the patient's shoes, be a TB patient, do you think it would be possible for us to visit the health centre for six months? Ruin our careers and everything to attend the health centre? We need to implement innovative ideas to support treatment adherence that are patient-centred rather than program-centred."*
People diagnosed with TB	Healthcare system, TB diagnosis and care delivery	Poor TB service delivery	Care not being patient-centred acting as barrier to treatment adherence	**FGD with people diagnosed with TB, 20-year-old male:** *"I stayed for one hour in queue and then waited a long time for treatment, spent money, and got a delayed report."*

Female: diagnosed with TB	Healthcare system, TB diagnosis and care delivery	Poor TB service delivery	Care not being patient-centred acting as barrier to treatment adherence	**FGD with female: diagnosed with TB, 18-year-old female:** *"Getting a delayed test report was a barrier for me, we had to come and go [to health facilities] for 4 days."*
People diagnosed with MDR-TB	Healthcare system, TB diagnosis and care delivery	Poor TB service delivery	Care not being patient-centred acting as barrier to treatment adherence	**FGD with people diagnosed with MDR-TB, 48-year-old male:** *"Our community mobiliser told us to go health post and they will administer the injection [injectable medication for TB]. I went but they told me that they couldn't give me an injection at that time."*
Community leaders	Healthcare system, TB diagnosis and care delivery	Poor TB service delivery	Care not being patient-centred acting as barrier to treatment adherence	**FGD with community leaders, 45-year-old male:** *"The behaviour of NTP healthcare workers needs to change. There's a difference between saying 'your father' and 'your respected father' isn't it? Imagine how bad an ill person will feel….when spoken to in an ill-mannered way; the speech will feel like an arrow."*
TB healthcare professional	Healthcare system, TB diagnosis and care delivery	Poor TB service delivery	Care not being patient-centred acting as barrier to treatment adherence	**FGD with TB healthcare professional, 55-year-old male:** *"The perception of lack of patient friendly services was also acknowledged as a problem in the NTP and patient FGDs and that although there are 4200 DOTS centres and many microscopic and GeneXpert centres, many are not patient friendly and this can contribute to delayed initiation of treatment."*

For peer review only

People Diagnosed with TB	Healthcare system, TB diagnosis and care delivery	Poor TB service delivery	Care not being patient-centred acting as barrier to treatment adherence	**FGD with people diagnosed with TB, 25-year-old male:** *"They don't ask anything. They just give medicines and give a tick mark in the card. It is just this at our place. They make a register. But if the main doctor [health worker] is not there, they don't even do it. They ask to take medicines by ourselves. In our village, we are asked to take medicines from the box."*
Category 4b: TB diagnosis and care delivery facilitators				
**FGD**	**First order categories**	**Second order themes**	**Third order themes**	**Quotes**
Civil society organization	Healthcare system, TB diagnosis and care delivery	Standardization of TB treatment	Proper regulation and management of TB services delivery	**FGD with civil society organization, 49-year-old male:** *"If TB medicines are only available from registered governmental health facilities, people will take them from there."*
People diagnosed with TB	Healthcare system, TB diagnosis and care delivery	Standardization of TB treatment	Proper regulation and management of TB services delivery	**FGD with people diagnosed with TB, 32-year-old male:** *"The main point is TB medicine is available in pharmacies and those who can pay will get it because pharmacies want money. Pharmacies don't care much about treatment and cure….or if patients take medicines at the right time or at all. That is why….TB medicines should only be provided under the supervision of government health facilities only. This will help to control TB."*

For peer review only

People diagnosed with MDR-TB	Healthcare system, TB diagnosis and care delivery	Improved access to TB diagnostic and treatment services	Provision of community based healthcare services and patient-centred care	**FGD with people diagnosed with MDR-TB, 44-year-old male:** *"What I like the most is they come to our houses to collect sputum."*
TB healthcare professional	Healthcare system, TB diagnosis and care delivery	Availability of diagnostic services	Early diagnosis of TB	**FGD with TB healthcare professional, 33-year-old male:** *"GeneXpert, X-ray and symptoms are three things that the program is focusing on."*
Community leaders	Healthcare system, TB diagnosis and care delivery	Improved access to TB diagnostic and treatment services	Provision of community based healthcare services and patient-centred care	**FGD with community leaders, 45-year-old male:** "We conduct screening camps for other diseases so why can't we do screening camp for tuberculosis? In the past, NGOs collected sputum from households where there was a patient on the basis of exposure history in family members. If we could collect and screen by understanding the patient's location that easier for them or visiting their workplace, it might increase the number of cases identified."

For peer review only

Female: diagnosed with TB	Healthcare system, TB diagnosis and care delivery	Improved access to TB diagnostic and treatment services	Provision of community based healthcare services and patient-centred care	**FGD with female: diagnosed with TB, 45-year-old female:** *"It's good to bring medicine at home. Sometimes, we have work at the time when we have to go to take medicines."*
Female: diagnosed with TB	Healthcare system, TB diagnosis and care delivery	Good behavior of healthcare providers	Patient-centred care	**FGD with female: diagnosed with TB, 60-year-old female:** *"The doctor treated me like a normal patient, cared for me, asked how I was feeling and gave me medicine."*
Community Mobilizers	Healthcare system, TB diagnosis and care delivery	Good behavior and communication between patient and healthcare providers	Patient-centred care	**FGD with Community Mobilizers, 40-year-old male:** *"We provide time to patients….meet them on their way to work or when coming back from work…..they are in constant contact with us…..we consult them properly…they trust us more [than doctors]."*
Female: diagnosed with TB	Healthcare system, TB diagnosis and care delivery	Proper communication between patient and healthcare providers	Patient-centred care	**FGD with female: diagnosed with TB, 45-year-old female: "***Doctor told that medicines should be taken for six months completely."*

For peer review only

TB healthcare professional	Healthcare system, TB diagnosis and care delivery	Standardization of TB treatment and referral services	Proper regulation and management of TB services delivery	**FGD with TB healthcare professional, 33-year-old male:** *"Government hospitals should mandate pharmacies and private providers to refer people with TB to government services but it was noted that legislation was insufficient."*
People diagnosed with TB	Healthcare system, TB diagnosis and care delivery	Standardization of TB treatment and referral services	Proper regulation and management of TB services delivery	**FGD with people diagnosed with TB, 32-year-old male: "***TB medicines should only be provided under the supervision of government health facilities only. This will help to control TB."*
TB healthcare professional	Healthcare system, TB diagnosis and care delivery	Decentralization of advanced diagnostic services in	Expansion of advanced diagnostic services	**FGD with TB healthcare professional, 55-year-old male:** *"Decentralization of GeneXpert had revolutionized the TB diagnosis in the hills of Nepal to do better diagnosis and find MDR TB roll out GeneXpert."*
TB healthcare professional	Healthcare system, TB diagnosis and care delivery	Prioritization of vulnerable groups for increased diagnosis	Strategies and policies for TB diagnosis	**FGD with TB healthcare professional, 55-year-old male: "***I think we have to change our strategy of active case finding, based on the level of vulnerability and the level of risk that a person can be exposed to. If we are following the same strategy for prisoners that we are following in the community, we might not find many cases."*

For peer review only

For peer review only

Wellcome Open Research
Wellcome Open Research 2020, 5:19 Last updated: 11 SEP 2020

Check for updates

STUDY PROTOCOL

REVISED Research protocol for a mixed-methods study to characterise and address the socioeconomic impact of accessing TB diagnosis and care in Nepal [version 2; peer review: 2 approved]

Kritika Dixit[1,2], Bhola Rai[1], Tara Prasad Aryal[1], Gokul Mishra[1], Noemia Teixeira de Siqueira-Filha[3], Puskar Raj Paudel[4], Jens W. Levy[4], Job van Rest[4], Suman Chandra Gurung[1,3], Raghu Dhital[1], Olivia Biermann[2], Kerri Viney[2], Knut Lonnroth[2], S Bertel Squire[3,5], Maxine Caws[1,3], Tom Wingfield[2,3,5]

[1]Birat Nepal Medical Trust, Lazimpat Road, Lazimpat, Ward No 2, Box 20564, Kathmandu, Nepal
[2]Social medicine, Infectious diseases, and Migration (SIM) Group, Department of Public Health Sciences, Karolinska Institute, Solnavägen 1, 171 77 Solna, Stockholm, Sweden
[3]Departments of International Public Health and Clinical Sciences, Liverpool School of Tropical Medicine, Pembroke Place, Liverpool, L3 5QA, UK
[4]KNCV Tuberculosis Foundation, Postbus 146, 2501 CC Den Haag, The Netherlands
[5]Tropical and Infectious Disease Unit, Liverpool University Hospitals NHS Foundation Trust, Prescot Street, Liverpool, L7 8XP, UK

For peer review

v2 **First published:** 05 Feb 2020, **5**:19
https://doi.org/10.12688/wellcomeopenres.15677.1
Latest published: 16 Jun 2020, **5**:19
https://doi.org/10.12688/wellcomeopenres.15677.2

Abstract
Background: WHO's 2015 End TB Strategy advocates social and economic (socioeconomic) support for TB-affected households to improve TB control. However, evidence concerning socioeconomic support for TB-affected households remains limited, especially in low-income countries.

Protocol: This mixed-methods study in Nepal will: evaluate the socioeconomic impact of accessing TB diagnosis and care (Project 1); and create a shortlist of feasible, locally-appropriate interventions to mitigate this impact (Project 2). The study will be conducted in the Chitwan, Mahottari, Makawanpur, and Dhanusha districts of Nepal, which have frequent TB and poverty.

The study population will include: approximately 200 people with TB (Cases) starting TB treatment with Nepal's National TB Program and 100 randomly-selected people without TB (Controls) in the same sites (Project 1); and approximately 40 key in-country stakeholders from Nepal including people with TB, community leaders, and TB healthcare professionals (Project 2).

Open Peer Review

Reviewer Status ✔ ✔

	Invited Reviewers	
	**1**	**2**
**version 2** (revision) 16 Jun 2020	✔ report	✔ report
**version 1** 05 Feb 2020	? report	? report

1. **Delia Boccia**, London School of Hygiene and Tropical Medicine, London, UK

2. **Priya B. Shete**, University of California San Francisco, San Francisco, USA

During Project 1, visits will be made to people with TB's households during months 3 and 6 of TB treatment, and a single visit made to Control households. During visits, participants will be asked about: TB-related costs (if receiving treatment), food insecurity, stigma; TB-related knowledge; household poverty level; social capital; and quality of life.

During Project 2, stakeholders will be invited to participate in: a survey and focus group discussion (FGD) to characterise socioeconomic impact, barriers and facilitators to accessing and engaging with TB care in Nepal; and a one-day workshop to review FGD findings and suggest interventions to mitigate the barriers identified.

Ethics and dissemination: The study has received ethical approval. Results will be disseminated through scientific meetings, open access publications, and a national workshop in Nepal.

Conclusions: This research will strengthen understanding of the socioeconomic impact of TB in Nepal and generate a shortlist of feasible and locally-appropriate socioeconomic interventions for TB-affected households for trial evaluation.

Keywords
Tuberculosis, poverty, catastrophic costs, socioeconomic support, social protection, healthcare access, Nepal

Any reports and responses or comments on the article can be found at the end of the article.

Corresponding author: Tom Wingfield (tom.wingfield@lstmed.ac.uk)

Author roles: Dixit K: Data Curation, Formal Analysis, Investigation, Project Administration, Visualization, Writing – Original Draft Preparation, Writing – Review & Editing; **Rai B**: Data Curation, Investigation, Project Administration, Writing – Review & Editing; **Prasad Aryal T**: Investigation, Project Administration, Writing – Review & Editing; **Mishra G**: Investigation, Methodology, Supervision, Writing – Review & Editing; **Teixeira de Siqueira-Filha N**: Data Curation, Formal Analysis, Investigation, Methodology, Project Administration, Writing – Original Draft Preparation, Writing – Review & Editing; **Raj Paudel P**: Data Curation, Project Administration, Writing – Review & Editing; **Levy JW**: Data Curation, Resources, Validation; **van Rest J**: Data Curation, Resources, Writing – Review & Editing; **Chandra Gurung S**: Investigation, Resources, Supervision, Writing – Original Draft Preparation, Writing – Review & Editing; **Dhital R**: Investigation, Project Administration, Resources, Writing – Original Draft Preparation, Writing – Review & Editing; **Biermann O**: Methodology, Writing – Review & Editing; **Viney K**: Methodology, Supervision, Writing – Original Draft Preparation, Writing – Review & Editing; **Lonnroth K**: Conceptualization, Methodology, Supervision, Writing – Original Draft Preparation, Writing – Review & Editing; **Squire SB**: Conceptualization, Methodology, Supervision, Writing – Original Draft Preparation, Writing – Review & Editing; **Caws M**: Conceptualization, Formal Analysis, Funding Acquisition, Investigation, Methodology, Project Administration, Supervision, Writing – Original Draft Preparation, Writing – Review & Editing; **Wingfield T**: Conceptualization, Data Curation, Formal Analysis, Funding Acquisition, Investigation, Methodology, Project Administration, Supervision, Validation, Visualization, Writing – Original Draft Preparation, Writing – Review & Editing

Competing interests: No competing interests were disclosed.

Grant information: This work was supported by grants to TW from the Wellcome Trust [209075] and the Academy of Medical Sciences, UK.
The funders had no role in study design, data collection and analysis, decision to publish, or preparation of the manuscript.

Copyright: © 2020 Dixit K *et al*. This is an open access article distributed under the terms of the Creative Commons Attribution License, which permits unrestricted use, distribution, and reproduction in any medium, provided the original work is properly cited.

How to cite this article: Dixit K, Rai B, Prasad Aryal T *et al.* **Research protocol for a mixed-methods study to characterise and address the socioeconomic impact of accessing TB diagnosis and care in Nepal [version 2; peer review: 2 approved]** Wellcome Open Research 2020, **5**:19 https://doi.org/10.12688/wellcomeopenres.15677.2

First published: 05 Feb 2020, **5**:19 https://doi.org/10.12688/wellcomeopenres.15677.1

For peer review only - http://bmjopen.bmj.com/site/about/guidelines.xhtml

REVISED **Amendments from Version 1**

In response to the reviewers' constructive comments, we have updated the following:

1. Provided more detail concerning coding methods for Project 2
2. Provided clarification concerning the selection and recruitment of controls, including limitations and weaknesses in this method
3. Included a conceptual framework that informed the study design and analysis (new Figure 3)
4. Included a logic model on the development of the socioeconomic intervention, in line with MRC methods (new Figure 4)
5. Clarified on data collection relating to social protection

These comments have been helpful in improving the protocol and we are grateful to the reviewers for their time and expertise.

Any further responses from the reviewers can be found at the end of the article

Introduction

Tuberculosis (TB) disease, which kills 1.5 million people annually, is driven by poverty[1]. Having TB disease can also worsen impoverishment through loss of income and costs of accessing care[2–5]. Such costs can become "catastrophic", leading patients to abandon treatment, develop drug-resistance, and die[6]. WHO's 2015 End TB Strategy advocates elimination of catastrophic costs and provision of socioeconomic support for TB-affected households[5]. Nevertheless, there is minimal evidence concerning the ideal interventions to realise this policy change[6–10]. This research will generate preliminary evidence to fill this knowledge gap in a low-income country: Nepal. The findings will inform a randomised controlled trial of socioeconomic support for TB-affected households in Nepal.

Research from Peru has demonstrated that the severe socioeconomic impact of TB can lead to catastrophic costs (defined by WHO as >20% of a household's annual income)[6]. The same team also demonstrated that socioeconomic interventions for TB-affected households can mitigate catastrophic costs (Figure 1a), improve TB preventive therapy uptake (Figure 1b), and increase TB treatment success (Figure 1c)[11–15].

However, despite these encouraging findings, Peru is a middle-income country with a strong TB program and existing national cash-transfer schemes. To make this research replicable and applicable in diverse settings, interventions similar to those in Peru need to be adapted to other country contexts, especially low-income countries (LICs) with less developed social protection schemes and high TB burden. One such country is Nepal.

The estimated incidence of TB in Nepal is 154/100,000 people with 45,000 cases of TB being notified. Amongst these cases, there were 6,800 TB-related deaths. Multi-drug resistance (MDR) rates were 2.2% in new cases and 15% among retreatment cases. Importantly, despite good treatment success rates overall of more than 90% reported in Nepal, there remain shortcomings in TB care: treatment success rates were 70% for patients with MDR-TB and only 9% for those with HIV-TB co-infection; and accessibility of TB care remains low with treatment coverage of 70%[16].

The poor treatment coverage in Nepal may, in part, be due to the financial impact on TB-affected households, which is estimated to be high[17]. Responding to this estimated burden, the Nepal National TB Programme (NTP) national strategic plan for 2016–2021 identifies provision of a support package to TB-affected households (specifically targeting MDR-TB-affected households) as a priority aim for the country with the goal of reducing catastrophic costs. There is currently limited evidence concerning the potential acceptability, impact, or cost-effectiveness of such a package, with which to inform and guide this policy decision. A cohort study has suggested that, during their illness, TB patients in Nepal experience decreased income and the total costs of accessing free TB treatment equate to nearly one quarter of annual household income[17]. Further studies in Nepal have shown that clinic fees make up the largest proportion of direct costs and that TB patients who are poorer, are migrants, or are from rural areas, experience a disproportionate burden of total costs[18–20]. It is vital that vulnerable TB patient households most at risk of incurring TB-related costs are identified in order to prevent and cure TB and mitigate further impoverishment and its consequences. In non-randomized studies, incurring higher TB-related costs and not receiving education about TB have both been found to be associated with worse TB treatment adherence and adverse TB treatment outcomes[18,21]. Therefore, the financial impact of having TB disease in Nepal constitutes a challenge to achieving TB control and elimination.

To date, no study of TB-affected households' costs in Nepal has been performed using the standardised global methodology for measuring costs related to accessing and engaging with TB care: the WHO TB Patient Costs Survey[22,23]. The studies above collected data at only one time point rather than repeated time points (e.g. cross-sectional rather than longitudinal) and did not robustly analyse the socioeconomic position, nutritional status, coping strategies, or linkage to social protection of TB-affected households. There have been no trials of socioeconomic support for TB-affected households in Nepal but formative qualitative analysis and a non-randomized pilot interventional study (offering education and financial support to patients with MDR-TB) suggested improved treatment outcomes[24].

However, studies reporting quantitative data alone will be insufficient to influence and change policy. Interaction with and among stakeholders has been described as the key facilitator for knowledge translation and evidence-informed health policymaking[25]. These interactions can include policy dialogues between stakeholders to deliberate on a priority topic. Therefore, complementary to quantitative data, the focus group

For peer review only - http://bmjopen.bmj.com/site/about/guidelines.xhtml

Wellcome Open Research 2020, 5:19 Last updated: 11 SEP 2020

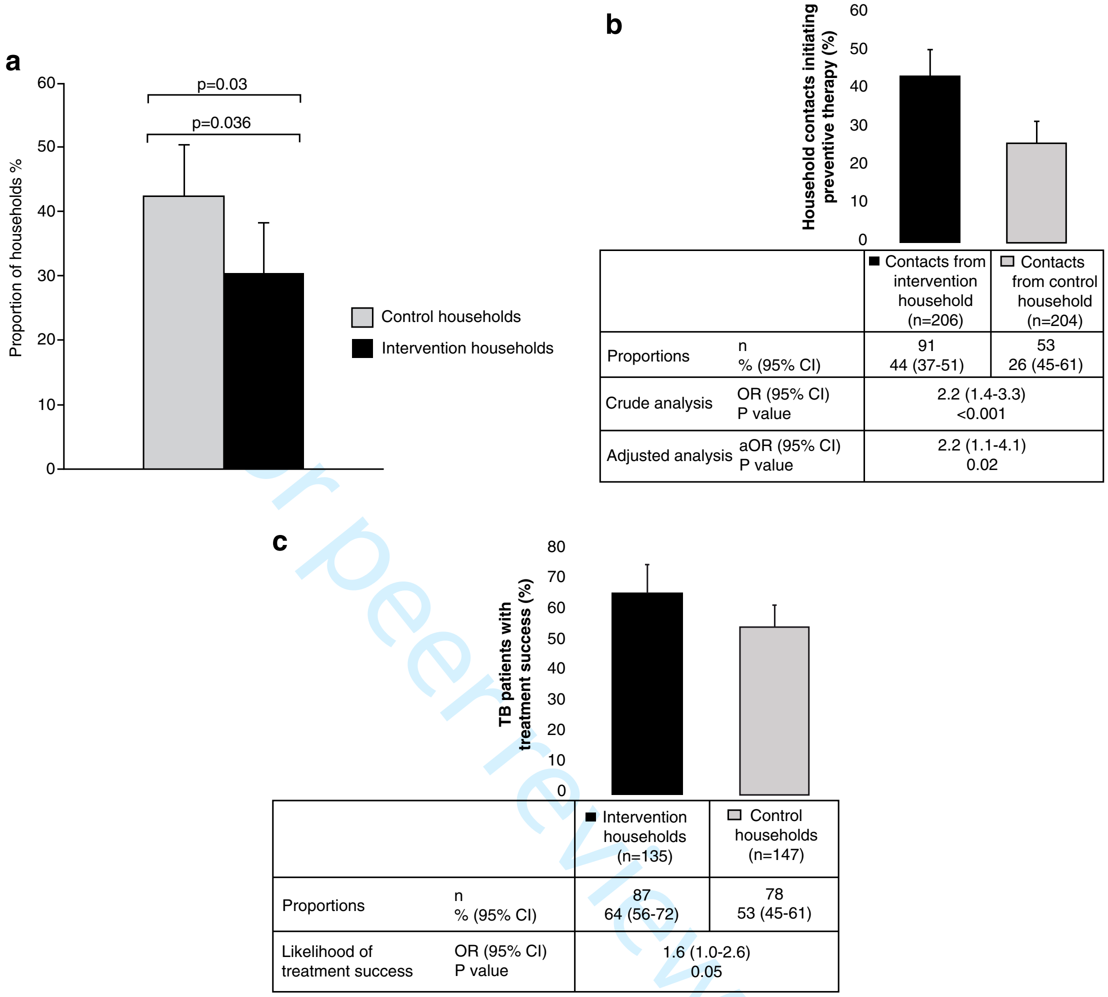

		■ Contacts from intervention household (n=206)	□ Contacts from control household (n=204)
Proportions	n<br>% (95% CI)	91<br>44 (37-51)	53<br>26 (45-61)
Crude analysis	OR (95% CI)<br>P value	2.2 (1.4-3.3)<br><0.001	
Adjusted analysis	aOR (95% CI)<br>P value	2.2 (1.1-4.1)<br>0.02	

		■ Intervention households (n=135)	□ Control households (n=147)
Proportions	n<br>% (95% CI)	87<br>64 (56-72)	78<br>53 (45-61)
Likelihood of treatment success	OR (95% CI)<br>P value	1.6 (1.0-2.6)<br>0.05	

Figure 1. **a**) Catastrophic costs incurred by intervention (n=135) and control (n=147) TB-affected households of Callao, Peru. **b**) Preventive therapy initiation in household contacts of intervention (n=206) and control (n=204) TB-affected households of Callao, Peru. **c**) TB treatment success in patients from intervention (n=135) and control (n=147) TB-affected households of Callao, Peru. Part **a**) has been reproduced with permission from Wingfield *et al.*[13] Parts **b**) and **c**) have been reproduced with permission from Wingfield *et al.*[15].

For peer review only

discussions (FGDs) and workshop detailed within this protocol will aim to expand a policy dialogue on socioeconomic support for TB-affected households among key stakeholders in Nepal, from people with TB to community leaders to TB healthcare professionals. Policy dialogues represent knowledge translation to support the integration of research evidence with tacit knowledge of local health policy-makers to: inform future policy decisions in often complex and dynamic contexts; and foster proactive collaboration[26,27]. These elements will be critical for successful implementation of any future interventions[28].

In summary, this mixed-methods research will complement and extend the existing knowledge base on social determinants and consequences of TB. More specifically, the culmination of the research will be the creation of a shortlist of feasible and locally-appropriate socioeconomic interventions for TB-affected households for future randomized controlled trial evaluation in Nepal.

Protocol

Ethical statement

Ethical approval was granted from the University of Liverpool, UK, research ethics committee in April 2018 (approval number 2436) and then the National Health Research Council of Nepal (NHRC) research ethics committee in May 2018 (approval number 320/2018).

Participant information leaflets will be provided and written informed consents will be obtained from all study participants for Project 1 Interviews (separate consent forms and information leaflets for patients and healthy controls), Project 2 Surveys, FGDs, and the workshop. These documents are available in the Extended Data section attachments[29].

All medical records obtained from the Nepal NTP will be kept confidential. Practically, through liaison with NTP Project Staff (as already organised for IMPACT-TB), the PM, PI, and

For peer review only - http://bmjopen.bmj.com/site/about/guidelines.xhtml

RA will photocopy patient records from the Nepal NTP TB register obscuring the patient's identifiable details. Photocopies will be marked with that patient's unique study number identifier. No individual patients will be identifiable from publications resulting from this study.

Study design

This mixed-methods study was funded by a Wellcome Trust Seed Award in Science (awards provided to early-career researchers to develop a novel idea that will go on to form part of a larger grant application) and will be divided into two complementary projects. Project 1 consists of a cohort study characterising the socioeconomic impact of TB on TB-affected households and a nested case-control study examining the social determinants of TB. Project 2 consists of a mixed quantitative-qualitative cross-sectional study using surveys, FGDs and a workshop to identify the barriers and facilitators to accessing and engaging with TB diagnosis and care in Nepal and suggesting potential interventions to mitigate the socioeconomic impact and improve access and engagement. The study will take place within the infrastructure of the larger EU-Horizon 2020 funded "IMPACT-TB" project, which is a study evaluating proven TB active case-finding (ACF) interventions in Nepal and Vietnam (grant 733174, http://www.impacttbproject.org/).

Primary aims

The primary aim of Project 1 is to evaluate the socioeconomic impact on TB-affected households of accessing and engaging with TB diagnosis and care in Nepal and compare that impact in people with TB identified through standard passive case finding (PCF) versus ACF. The primary aim of Project 2 is to collaborate with key stakeholders in Nepal to create a shortlist of potentially feasible and locally-appropriate socioeconomic interventions to mitigate this impact.

Secondary aims

The secondary aim of Project 1 is to compare the social determinants of TB (including socioeconomic position, housing situation, knowledge about TB, comorbidities, quality of life, food security, and social capital) in people with TB versus people without TB from the same districts. The secondary aim of Project 2 is to collate the opinions of key stakeholders from diverse sectors about barriers and facilitators to accessing and engaging with TB care in Nepal.

An additional aim across the study is to generate a policy dialogue and form a collaborative research network to support development and implementation of a future randomised control trial of socioeconomic support for TB-affected households in Nepal.

Study setting

The study will take place within the infrastructure of the larger IMPACT-TB study, which works with a well-established international non-governmental organisation, Birat Nepal Medical Trust (BNMT), to implement ACF activities including sputum-microscopy camps and roll-out of GeneXpert OMNI in four intervention and two control districts (with PCF only). The four districts are located in the central development region of Nepal and were selected for the IMPACT-TB project based on comparable populations and TB case detection rates (Figure 2).

The primary outcome of IMPACT-TB is the effect of ACF on early TB case detection and the study does not involve support packages for TB-affected households during treatment. This presents a unique opportunity for the complementary research described in this protocol to add value to IMPACT-TB, explore the socioeconomic impact of accessing TB care, and shortlist locally-driven strategies to reduce this impact.

Study population

Participant identification, recruitment and follow-up. During Project 1, approximately 200 people with TB (cases) consecutively recruited to Work Package 3 (WP3) of IMPACT-TB (Health Economic Analysis) in the four study sites will be further recruited to this study. 100 of these cases will have been diagnosed through ACF and 100 diagnosed through PCF. Whilst attending the NTP TB clinic, these people with TB will be invited to participate in this mixed-methods research with a separate written, informed consent (see Project 1 Interview Patient Consent Form, *Extended data*)[29]. During months three and six of their TB treatment, household visits will be conducted by BNMT project staff including community mobilisers.

Concurrently, 100 people without TB from the study sites (controls) will be invited to participate with written, informed consent (see Project 1 Interview Healthy Control Consent Form, *Extended data*)[29]. Due to constraints in study budget, duration, and field logistics, it will not be possible to age and sex match cases and controls nor to randomly select controls using geospatial or other household randomisation techniques. Therefore, a convenience sampling strategy was used. In order to be as widely representative of and generalisable to the background population as possible, following project team and community mobiliser discussions in the study sites, we opted to recruit control participants from diverse locations within the districts, which were attended by a broad demographic cross-section of the local population in terms of age, gender, and socioeconomic position. These diverse study site locations include: tea houses, primary healthcare centres, antenatal and immunization clinics, door-to-door visits following sputum camps (e.g. people who tested negative for TB), and public gathering places. Interviewers will visit the control recruitment locations at similar times of the morning and aim to consecutively recruit all individuals in attendance at that location. Similar numbers of participants from each recruitment location will be recruited until the sample size of 100 people without TB is reached. We acknowledge that convenience sampling may be associated with a higher likelihood of a non-generalisable control cohort than other techniques. To try to address this, the survey used to collect data from the controls has incorporated multiple questions concerning sociodemographic variables, including education level, occupation, amenities, and assets, from the most recent version of the Nepal Household Survey, which is publicly available. Descriptive

For peer review only - http://bmjopen.bmj.com/site/about/guidelines.xhtml

Wellcome Open Research 2020, 5:19 Last updated: 11 SEP 2020

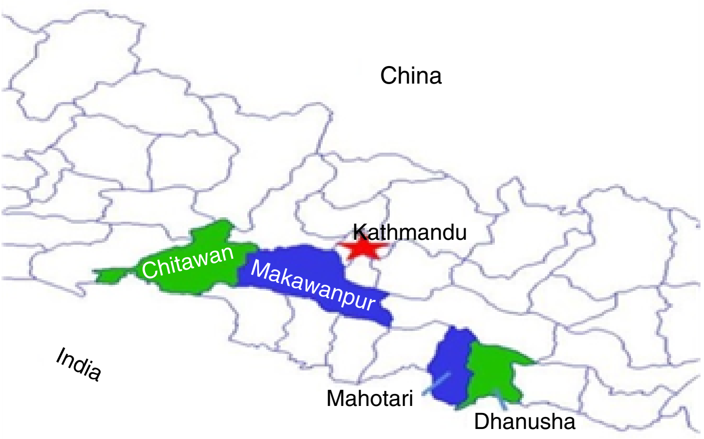

District	Population	Case Notification Rate (per 100,000 population)
Makwanpur	480,000	147
Chitwan	585,000	158
Dhanusha	823,000	100
Mahottari	681,000	131

Figure 2. Map, population, and TB case notification rate of the four study site districts. Data for population and TB cases notification rate shown is taken from the Nepal TB Centre 2018 report. The four study site districts are labelled and highlighted in blue and green.

analysis will evaluate whether the controls recruited are representative of the wider population in those districts through comparison of their sociodemographic data with respondents to the national survey in the same districts. Any differences between the control population and background population will then be highlighted transparently in corresponding research outputs and publications.

Project 1 inclusion criteria for Cases included: being a person with TB notified to the NTP and recruited to WP3 of the IMPACT-TB study; being aged 18 years or above; and giving verbal and written informed consent to participate. Project 1 exclusion criteria for Cases included: being under 18 years of age; being a person with TB not notified to the NTP and/or not recruited to WP3 of the IMPACT-TB study; being a person with TB notified to the NTP but with a recorded domiciliary address outside of the study site districts; and being unable or unwilling to give written and/or verbal informed consent to participate.

Project 1 inclusion criteria for Controls included: being 18 years or above with primary residence in the study site communities; not currently known to be a person with TB or have a member of the household currently known to be a person with TB (e.g. not diagnosed or notified or receiving TB treatment); and giving verbal and written informed consent to participate. Project 1 exclusion criteria for Controls included: being under 18 years of age; not having primary residence in the study site communities; known to be a person with TB or have a household member with TB currently (e.g. diagnosed and/or notified and/or receiving TB treatment); and being unable or unwilling to give verbal and written informed consent to participate.

To generate the population for Project 2, a literature review and desk-based scoping review will identify a list of key in-country stakeholders from Nepal from diverse groups including: civil-society representatives; community leaders; and TB healthcare professionals including NTP managers and multi-disciplinary staff. Approximately 50 stakeholders will be selected through purposive sampling and invited to participate in: a pre-FGD survey, FGD, and a one-day workshop. A subset of purposively sampled TB patients recruited to Project 1 (including those with multi-drug resistant TB) will be among the stakeholders invited to participate.

Project 2 inclusion criteria include: being aged 18 years or above; belonging to a stakeholder group as defined above and/or identified during scoping exercise; and being able and willing to provide verbal and written, informed consent. Exclusion criteria for Project 2 are not meeting the inclusion criteria and/or being a person with TB who has not yet taken two weeks of TB treatment or is otherwise considered to still be infectious (e.g. MDR-TB with positive sputum smear or culture).

For peer review only - http://bmjopen.bmj.com/site/about/guidelines.xhtml

Sample size and statistical power. The sample size for Project 1 is approximately 200 Cases recruited to WP3 of the IMPACT-TB project and 100 Controls from the four study site districts. This sample size is opportunistic and pragmatic: related research suggests that data from 100 people with TB gives a representative spread of costs for a given context[30,31].

For Project 2, each FGD will consist of approximately eight stakeholders. The estimated number of FGDs at which information power (or saturation level) will be reached is six[12]. Thus, the sample size for Project 2 is approximately 40 stakeholder participants. We will invite 55 stakeholders as a contingency because we anticipate an attrition rate of 20–30% during the course of Project 2.

Given that this Seed Award research will be exploratory, preparatory (e.g. for the future trial), and does not include an intervention, no calculations of statistical power are required.

Study interventions

The study will not include any interventions or require any patient samples (e.g. blood / sputum / tissue).

Study activities

Broadly, the activities involved in the research will include interviews with Cases and Controls during household visits, and a pre-FGD survey, FGD, and workshop with key stakeholders.

During Project 1, the project team will support BNMT district coordinators, community mobilisers, and community volunteers to do household visits to approximately 200 Cases with TB (100 diagnosed through ACF and 100 through PCF) recruited to WP3 of the IMPACT-TB project. People with TB will receive two household visits, the first during month three of TB treatment and the second visit during month six of TB treatment (to correspond with treatment completion). For controls, a single household visit and interview will be done. The interviews will be structured (see Project 1 Interview, *Extended data*)[29] and gather data on: i) socioeconomic position, evaluated by a multi-dimensional poverty score[6,9,13,15] assessing dwelling characteristics, assets, and access to amenities; ii) household structure, including distribution of age, sex, and employment of household members; iii) food expenditure and security; iv) costs of engaging with TB care including direct costs (e.g. medicines, clinic visits, food, and travel) and indirect costs (e.g. lost income), which will be evaluated using an adapted version of WHO's TB Patient Costs Survey integrated into the interview[22,23]; v) coping strategies including dissaving (e.g. selling assets), schooldays lost, and temporary income-generating activities; vi) TB-related knowledge including understanding of transmission, prevention, and treatment of TB; vii) psychosocial situation evaluated through questions relating to social capital, quality of life, and stigma (Controls without TB will not be asked any questions about the impact that having TB disease has on their psychosocial situation); and viii) access and uptake of existing social protection schemes (whether TB-specific or TB-inclusive) and support for TB-affected households, evaluated through use of both closed ranking and open free-text questions to establish what socioeconomic and other support people with TB and their households receive or would like to receive.

Prior to implementation of household visits, the questionnaire will be translated from English into Nepali and then back-translated into English. It will be assessed by members of the study team and BNMT implementation staff before being piloted in approximately 10 patients. The questionnaire may subsequently be refined and questions deleted or added, depending on the pilot outcomes.

In addition to the above, Cases' TB treatment outcomes will be collated from NTP and IMPACT-TB data and their association with interview responses analysed. This will provide an exploratory analysis of the association of socioeconomic position, socioeconomic impact of having TB, and TB treatment outcomes. A comparison of the socioeconomic position of households of Cases and Controls will also be made.

Project 2 will use and develop mixed methods research techniques[10–15] to conduct a pre-FGD survey, FGDs, and workshop with approximately 40 key stakeholders in Nepal to identify the socioeconomic impact, barriers and facilitators to accessing and engaging with TB diagnosis and care. To inform the design of the FGDs and workshop, we created a conceptual framework for the barriers and facilitators to TB diagnosis and care, which was adapted from a World Health Organisation framework for medication adherence (Figure 3)[32].

A short individual pre-FGD survey (see Project 2 Survey, *Extended data*)[29] will be provided to participants prior to the initiation of an FGD. The survey will detail: participants' demographics; the stakeholder group to which the participant belongs; and their opinions on community, patient, health system, existing social protection schemes, and wider obstacles to achieving successful TB treatment outcomes.

The FGDs will be semi-structured and incorporate open-ended questions concerning barriers and facilitators to accessing and engaging with TB care in Nepal, and existing platforms and potential opportunities to mitigate these barriers including social protection schemes (see Project 2 Focus Groups, *Extended data*)[29]. The FGDs will be conducted with separate groups of approximately eight key stakeholders. Stakeholders will be invited to participate according to their background (e.g. people with TB will be asked to participate in one FGD, and TB healthcare professionals will be asked to participate in another separate FGD). Towards the end of each FGD, participants will be asked to privately rank the top three most important barriers or facilitators to accessing TB diagnosis and care identified by the group during the FGD, and these responses will be collated for each FGD and across FGDs. The FGDs will be moderated by members of the project team trained in qualitative methods including conducting FGDs. The discussions will be audio recorded in Nepali language, translated into English, and back-translated by a translator who is not part of the project team. Each FGD group will be asked to elect a representative to feed their group's outputs back at the subsequent workshop.

For peer review only - http://bmjopen.bmj.com/site/about/guidelines.xhtml

Wellcome Open Research 2020, 5:19 Last updated: 11 SEP 2020

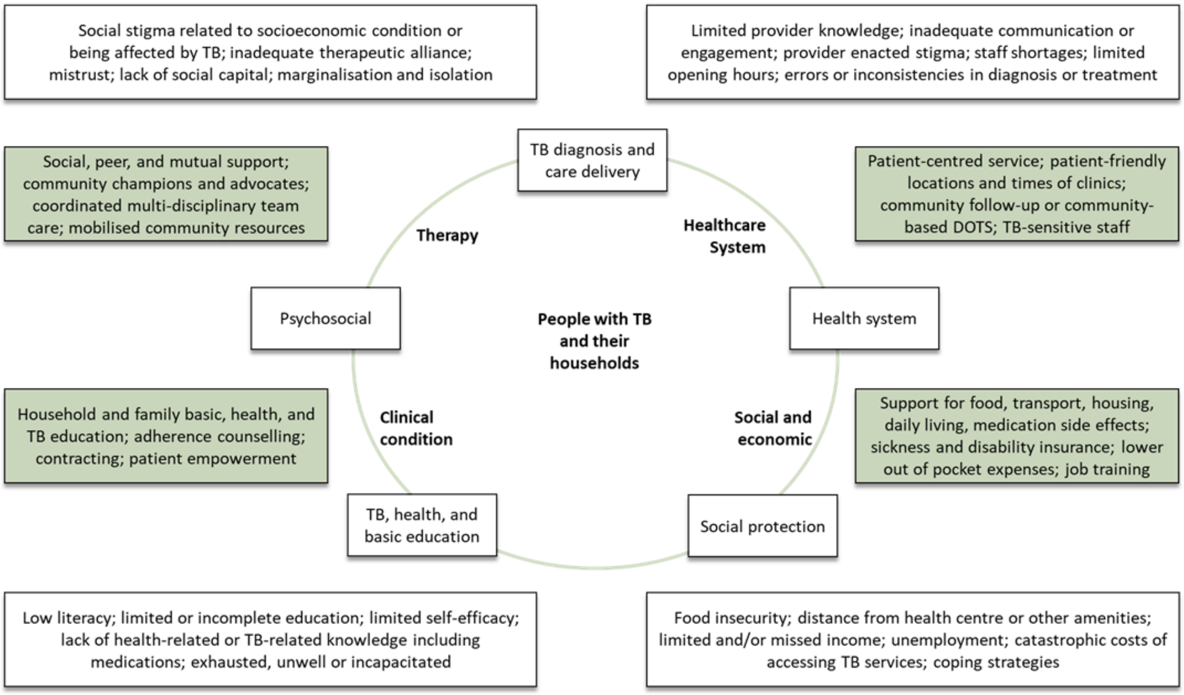

Figure 3. Conceptual framework: the barriers and facilitators to TB diagnosis and care. The central circle, which contains dimensions of medication adherence adapted from the World Health Organization[33], is surrounded by the five main categories of relevant potential interventions. The factors that may promote access to and engagement with TB services (facilitators) are shown in green boxes and factors that may threaten access and engagement (barriers) are shown in white boxes. Therapeutic alliance refers to strong provider-patient relationships. Although Project 2 would gather data on all five dimensions, the psychosocial and social protection dimensions were perceived by the project team to be most pertinent to development of the socioeconomic intervention and were selected for further focus and discussion during Project 2's workshop.

The final activity in Project 2 will be a one-day workshop bringing together the 40 key stakeholders (see Project 2 Workshop, *Extended data*)[29]. The morning section of the workshop will consist of interactive presentations from the project team and stakeholder group representatives (including leaders of national social protection schemes in Nepal), and discussions exploring and validating the barriers and opportunities identified during the pre-FGD survey and interviews during FGDs. The afternoon section of the workshop will consist of multi-sectoral working groups (≤10 diverse stakeholders) developing a shortlist of potential socioeconomic interventions for TB-affected households in Nepal. The shortlist is not intended to consist of defined, unalterable packages, which are immediately ready for trial implementation. Rather, the potential interventions selected are intended to consist of what stakeholders perceive to be key elements or ingredients of psychosocial and economic support for TB-affected households, which are feasible and locally-appropriate to the Nepalese context. The interventions will be presented to the group including strengths, weaknesses, and potential sources of funding for implementation.

The activities involved in this research form part of a process to develop and evaluate a complex, socioeconomic support intervention. In line with the Medical Research Council's guidance on process evaluation of complex interventions, we developed a logic model to illustrate the developmental stages of the intervention (Figure 4)[32].

This Wellcome research will aim to achieve the short-term outcomes described in the logic model. If follow-on funding is successfully obtained, the process to develop and implement the intervention and achieve the long-term outcomes will continue beyond this Wellcome research. This will include adaptation, piloting, and then large-scale randomised trial evaluation of the refined intervention.

Outcomes to be measured

This exploratory Wellcome Trust funded Seed Award in Science research in four districts of Nepal will:

i. characterise social determinants of TB by comparing poverty level, education level, food security, and other socioeconomic factors of people with TB (Cases) versus people without TB (Controls);

ii. provide new insight into the barriers, facilitators, and socioeconomic impact of being ill with TB and accessing TB diagnosis and care, and compare this in Cases diagnosed by ACF versus PCF; and

For peer review only - http://bmjopen.bmj.com/site/about/guidelines.xhtml

Wellcome Open Research 2020, 5:19 Last updated: 11 SEP 2020

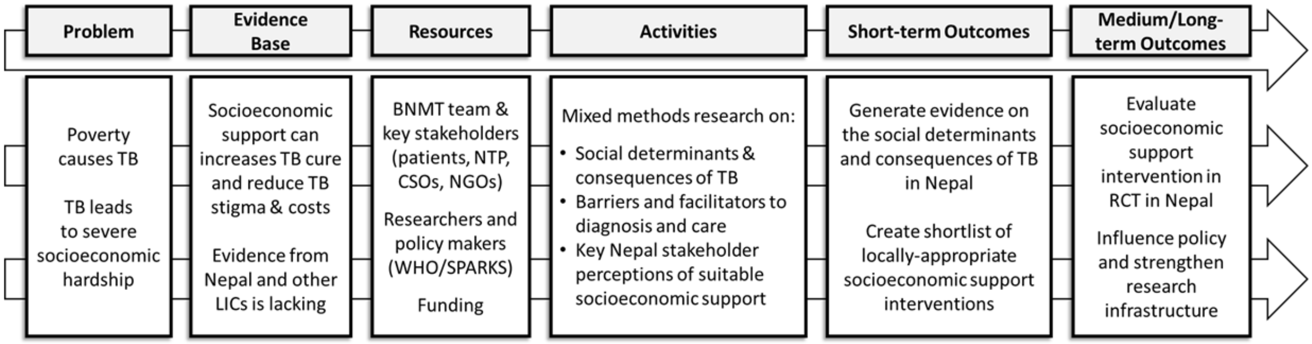

Figure 4. Logic model for research in Nepal to develop a locally-appropriate socioeconomic support intervention for TB-affected households. Abbreviations: CSOs = Civil Society Organisations; NGOs = Non-governmental organisations; NTP = National Tuberculosis Programme; WHO = World Health Organisation; SPARKS = Social Protection Action Research and Knowledge Sharing network (www.sparks.ki.se)

iii. generate a community-led shortlist of the most feasible, equitable, and locally-appropriate socioeconomic interventions for TB-affected households to mitigate the socioeconomic impact of TB.

Data collection and management

During the implementation of Project 1, information will be collected by BNMT district coordinators, community volunteers, and community mobilisers with support from the project team during visits to recruited patient households. This information includes but is not limited to socioeconomic, health, psychosocial, and behavioural data. This data will be collected on paper due to digital collection (e.g. on tablets or mobile phones) having issues with security and feasibility. The data of consenting TB patients will subsequently be linked with data from NTP's TB patient register as part of its routine surveillance data collection at the intervention and control areas, with pre-existing permission from the NTP.

During Project 2, key stakeholders identified by the scoping exercise will complete a short pre-FGD survey in person, participate in an FGD, and participate in a one-day workshop.

All paper-based copies, including medical records, informed consent forms and participant information leaflets, will contain only a unique study identifier for each participant. These documents will be stored in a locked room in the BNMT office. Data will be checked for consistency and completeness by the project manager and double-checked by the PI prior to entering into an encrypted access database. The database will be managed by the data management team at KNCV TB Foundation in The Netherlands in line with data collected during the IMPACT-TB project. The data will be protected by KNCV on a password-secured server with availability limited to only key members of the study team when required for analysis.

Data analysis and statistical plan

The quantitative data collected during the household visits of Project 1 will be analysed using simple descriptive statistics. Continuous costs data will be summarised by their arithmetic means and their 95% confidence intervals whether the data is Gaussian or non-Gaussian, because this approach is considered to be robust for health economics data analysis[6,34–36]. Furthermore, because of the skewed nature of some expenditure data, median values may be zero or close to zero limiting the descriptive usefulness of presenting median values. As described in the PI's previous research[6], any direct expenses, lost income, or annual income recorded as "zero" or missing will be replaced with the mean cost of each costs category, i.e. mean direct costs or lost income. The local currency, Nepalese rupee, will be converted into United States Dollars (conversion rate and date estimated through Oanda at time of data collection). Categorical data will be summarised as proportions with 95% confidence intervals.

With regards to analysis of household income and expenditure, the WHO TB Patients Costs Survey methods will be followed. In order to evaluate the optimal analytical strategy for costs data in Nepal, the analysis will compare and contrast the different approaches used in the WHO Survey to estimate household income (self-reported household consumption; self-reported household expenditure; self-reported household income; and estimated income based on household asset ownership and dwelling characteristics) and lost income (output approach of reported income pre- and during TB versus human capital approach of multiplying hours of work lost by hourly rate or daily in rate in cases of hospitalised patients).

Social determinants of TB including sociodemographic characteristics, socioeconomic position, stigma and social capital levels, and TB-related knowledge will be compared between ACF patients, PCF patients, and controls using Chi-squared test, Pearson's test, one-way ANOVA, and multiple logistic regression models where appropriate. No comparison will be made between patients and controls concerning healthcare expenditure as this data was not collected from controls. Statistical analysis will be performed using the statistical software package STATA v13.1 (Statacorp, TX, USA).

For peer review only - http://bmjopen.bmj.com/site/about/guidelines.xhtml

Wellcome Open Research 2020, 5:19 Last updated: 11 SEP 2020

The Framework method of thematic analysis will be used to manage and analyse data from Project 2 via the NVivo qualitative software package (Version 12) as per published social policy and tuberculosis research[21–24]. Specifically, two researchers (KD and TW) will familiarize themselves with the data through successive reading of transcripts; use both open and closed first order data coding to label data within NVivo; group codes together into a second order codebook of themes and sub-themes. Themes and subthemes will then be further stratified by third order coding to the level at which they predominantly occurred (e.g. individual or household level, health systems level, community level, or governmental level). The primary approach to codes, themes, and sub-themes will be deductive (top down), being informed by our conceptual framework and preliminary analysis of data from Project 1 relating to the social determinants and consequences identified. As coding continues, a secondary approach will integrate inductive (bottom up) coding in order to be iterative, responsive, and flexible as further data becomes available and is collated following each successive FGD.

Plans for dissemination of study findings

The intended research outputs of this work are to: i) present the interim and final findings at the International Union Against TB and Lung Disease in October 2019 and October 2020, respectively; ii) publish, by June 2020, at least two papers in high-impact, peer-reviewed journals concerning the socioeconomic impact of accessing TB care in Nepal and the collaborative development of a shortlist of locally-appropriate socioeconomic interventions in Nepal; iii) feedback findings to the IMPACT-TB team, key stakeholders (including NTP and TB civil-society), the SPARKS (Social Protection Action Research and Knowledge Sharing) network, and WHO; iv) consolidate a close collaboration, good working relationship, and strong research infrastructure between BNMT and the NTP; and iv) to develop the protocol for a robust, large-scale randomised controlled trial to evaluate socioeconomic support for TB-affected households using the evidence generated by this mixed-methods study.

Study status

Data collection for Project 1 during household visits began in May 2018 and is nearly complete at the time of writing with only TB treatment outcome data still being collected. Data collection for Project 2 during pre-FGD surveys and FGDs began in August 2018 and was completed in July 2019. The national workshop with key stakeholders was conducted on 11 and 12 September 2019 in Kathmandu, Nepal.

Data analysis, write-up, and dissemination of findings will begin in February 2020.

Discussion

The overarching pledge of the Sustainable Development Goals (SDGs) is to "leave no one behind". In 2018, over 3 million people with tuberculosis (TB) were not diagnosed, not notified, or their quality of care was unknown. In the same year, 1.5 million people with TB died and nearly a fifth of people diagnosed with TB did not have a successful treatment outcome[16]. While millions of people with TB continue to be left behind, the SDG pledge is far from being realised.

Despite renewed interest in addressing social determinants of tuberculosis, there remain stark global inequalities in disease burden and access to TB care[8]. At a population level, LICs bear the highest TB prevalence[16]. At an individual level, people with TB are often vulnerable, impoverished, and their households suffer disproportionate financial shock due to their illness[6,13]. To eliminate such gross disparity, SDG slogans must be turned into actions.

WHO's 2015 End TB Strategy acknowledges the need to reduce inequalities in TB prevention and care. A key component of Pillar 2 (Bold policies and supportive systems) of the strategy is social protection and poverty alleviation to reduce catastrophic costs of TB-affected households and improve TB outcomes[2]. However, there is minimal evidence to guide this policy change.

This mixed-methods will generate evidence concerning the socioeconomic position of TB-affected households, the impact that having TB disease has on that position and explore the coping strategies that households use to mitigate the impact of the disease. Moreover, this research will: use methods to measure costs of TB-affected households to which members of the study team contributed as part of the WHO Task Force on Catastrophic Costs of Tuberculosis; and provide the first known comparison of the socioeconomic impact of TB on people with TB diagnosed through PCF versus ACF. The case-control element of Project 1 will allow comparison of the sociodemographic characteristics, socioeconomic position, stigma and social capital levels, and TB-related knowledge between people with TB (Cases) and people without TB (Controls).

The further significance of the study lies in its development of a shortlist of a locally-generated intervention to provide socioeconomic support to TB-affected households. Through collaboration with diverse stakeholders in Nepal from patients to NTP managers to civil-society representatives, it is hoped that the shortlist created will feature interventions that are both locally-appropriate and feasible. Furthermore, this tight collaboration should aid design and implementation of a larger, randomised-controlled trial, and also future translation of research findings into national policy in Nepal. Beyond the national impact, these findings and those of the future trial will also offer evidence for scale-up of socioeconomic support in other resource-limited countries with a high TB burden. Complementary to pills and tests, this socioeconomic support will be an essential part of eliminating TB by 2050.

Conclusions

This mixed-methods study will fill this existing evidence gap by examining the costs of accessing TB care at a household level through longitudinal application of an adapted WHO TB Patient Costs Survey throughout TB treatment. This data will be enriched by collecting complementary data on household socioeconomic position, coping strategies, food insecurity,

For peer review only - http://bmjopen.bmj.com/site/about/guidelines.xhtml

Wellcome Open Research 2020, 5:19 Last updated: 11 SEP 2020

TB knowledge, social capital, and quality of life. The findings will then feed into a short survey and semi-structured FGDs with key stakeholders in Nepal to consider, at a local, regional, and national level, what are the leading barriers and facilitators to accessing and engaging in TB care, and what might be the most locally-appropriate interventions to address the socioeconomic impact of having TB disease.

Data availability

Underlying data

No data are associated with this article

Extended data

Open Science Framework: Research protocol for a mixed-methods study to characterise and address the socioeconomic impact of accessing TB diagnosis and care in Nepal. https://doi.org/10.17605/OSF.IO/6TC4F[29]

This project contains the following extended data:

- Final_Wellcome Seed Award Project 1 Patient Interview v8.pdf
- Final Patient _Wellcome Seed Award Project 1 Consent Form NEPALI.pdf
- Final Patient _Wellcome Seed Award Project 1 Interview v8 NEPALI.pdf
- Final Patient _Wellcome Seed Award Project 1 Patient Info Leaflet NEPALI.pdf
- Wellcome Seed Award Project 1 Patient Consent Form.pdf
- Wellcome Seed Award Project 1 Patient Information Leaflet ENGLISH.pdf
- Wellcome Seed Award Project 2 Survey and Focus Group Consent Form.pdf
- Wellcome Seed Award Project 2 Survey and Focus Group Participant Information Leaflet.pdf
- Wellcome Seed Award Project 2 Survey v4 20180525.pdf
- Wellcome Seed Award Project 2 Workshop Consent Form.pdf
- Wellcome Seed Award Project 2 Workshop Participant Information Leaflet.pdf

Data are available under the terms of the Creative Commons Zero "No rights reserved" data waiver (CC0 1.0 Public domain dedication).

References

1. Raviglione M, Krech R: **Tuberculosis: still a social disease.** *Int J Tuberc Lung Dis.* 2011; **15**(Suppl 2): 6–8.
PubMed Abstract | Publisher Full Text
2. Bhargava A, Pai M, Bhargava M, *et al.*: **Can social interventions prevent tuberculosis?: the Papworth experiment (1918–1943) revisited.** *Am J Respir Crit Care Med.* 2012; **186**(5): 442–449.
PubMed Abstract | Publisher Full Text
3. Janssens JP, Rieder HL: **An ecological analysis of incidence of tuberculosis and *per capita* gross domestic product.** *Eur Respir J.* 2006; **32**(5): 1415–1416.
PubMed Abstract | Publisher Full Text
4. Dye C, Lönnroth K, Jaramillo E, *et al.*: **Trends in tuberculosis incidence and their determinants in 134 countries.** *Bull World Health Organ.* 2009; **87**(9): 683–691.
PubMed Abstract | Publisher Full Text | Free Full Text
5. World Health Organisation's: **End TB Strategy.** 2020.
Reference Source
6. Wingfield T, Boccia D, Tovar M, *et al.*: **Defining catastrophic costs and comparing their importance for adverse tuberculosis outcome with multi-drug resistance: a prospective cohort study, Peru.** *PLoS Med.* 2014; **11**(7): e1001675.
PubMed Abstract | Publisher Full Text | Free Full Text
7. Wingfield T, Tovar MA, Huff D, *et al.*: **Socioeconomic support to improve initiation of tuberculosis preventive therapy and increase tuberculosis treatment success in Peru: a household-randomised, controlled evaluation.** *Lancet.* Academy of Medical Sciences Young Investigator Award. 2017; **389**(6).
Publisher Full Text
8. Wingfield T, Tovar MA, Datta S, *et al.*: **Addressing social determinants to end tuberculosis.** *Lancet.* 2018; **391**(10126): 1129–1132.
PubMed Abstract | Publisher Full Text
9. Wingfield T, Schumacher SG, Sandhu G, *et al.*: **The seasonality of tuberculosis, sunlight, vitamin D, and household crowding.** *J Infect Dis.* 2014; **210**(5): 774–83.
PubMed Abstract | Publisher Full Text | Free Full Text
10. Williamson J, Ramirez R, Wingfield T: **Health, healthcare access, and use of traditional versus modern medicine in remote Peruvian Amazon communities: a descriptive study of knowledge, attitudes, and practices.** *Am J Trop Med Hyg.* 2015; **92**(4): 857–864.
PubMed Abstract | Publisher Full Text | Free Full Text
11. Boccia D, Pedrazzoli D, Wingfield T, *et al.*: **Towards cash transfer interventions for tuberculosis prevention, care and control: key operational challenges and research priorities.** *BMC Infect Dis.* 2016; **16**: 307.
PubMed Abstract | Publisher Full Text | Free Full Text
12. Wingfield T, Boccia D, Tovar MA, *et al.*: **Designing and implementing a socioeconomic intervention to enhance TB control: operational evidence from the CRESIPT project in Peru.** *BMC Public Health.* 2015; **15**(1): 810.
PubMed Abstract | Publisher Full Text | Free Full Text
13. Wingfield T, Tovar MA, Huff D, *et al.*: **The economic effects of supporting tuberculosis-affected households in Peru.** *Eur Respir J.* 2016; **48**(5): 1396–1410.
PubMed Abstract | Publisher Full Text | Free Full Text
14. Wingfield T, Tovar MA, Huff D, *et al.*: **Beyond pills and tests: addressing the social determinants of tuberculosis.** *Clin Med (Lond).* 2016; **16**(Suppl 6): s79–s91.
PubMed Abstract | Publisher Full Text | Free Full Text
15. Wingfield T, Tovar MA, Huff D, *et al.*: **A randomized controlled study of socioeconomic support to enhance tuberculosis prevention and treatment, Peru.** *Bull World Health Organ.* 2017; **95**(4): 270–280.
PubMed Abstract | Publisher Full Text | Free Full Text
16. World Health Organisation: **Global Tuberculosis Report 2019.** WHO. 2019.
Reference Source
17. Gurung GN, Chhetri PS, Jha N: **Economic impact of pulmonary tuberculosis on patients and their families of Dharan municipality, Nepal.** *Nepal Med Coll J.* 2012; **14**(3): 196–198.
PubMed Abstract
18. Bam TS, Gunneberg C, Chamroonsawasdi K, *et al.*: **Factors affecting patient adherence to DOTS in urban Kathmandu, Nepal.** *Int J Tuberc Lung Dis.* 2006; **10**(3): 270–276.
PubMed Abstract
19. Kirwan DE, Nicholson BD, Baral SC, *et al.*: **The social reality of migrant men with tuberculosis in Kathmandu: implications for DOT in practice.** *Trop Med Int Health.* 2009; **14**(12): 1442–1447.
PubMed Abstract | Publisher Full Text
20. Ramsay A, Al-Aghbari N, Scherchand J, *et al.*: **Direct patient costs associated with tuberculosis diagnosis in Yemen and Nepal.** *Int J Tuberc Lung Dis.* 2010; **14**(2): 165–170.
PubMed Abstract
21. Mishra P, Hansen EH, Sabroe S, *et al.*: **Socio-economic status and adherence to tuberculosis treatment: a case-control study in a district of Nepal.** *Int J Tuberc Lung Dis.* 2005; **9**(10): 1134–1139.
PubMed Abstract

For peer review only

22. **Measuring patient costs to monitor progress towards the target to eliminate catastrophic costs and help design social protection and UHC.** Meeting of the WHO Global Task Force on TB Impact Measurement, 19–21 April 2016. GTB/WHO.
Reference Source

23. WHO: **Protocol for questionnaire to determine direct and indirect costs due to TB and to estimate proportion of TB-affected households experiencing catastrophic costs: field testing version**. Geneva: World Health Organization, 2015.
Reference Source

24. Baral SC, Aryal Y, Bhattrai R, *et al.*: **The importance of providing counselling and financial support to patients receiving treatment for multi-drug resistant TB: mixed method qualitative and pilot intervention studies.** *BMC Public Health.* 2014; **14**: 46.
PubMed Abstract | Publisher Full Text | Free Full Text

25. Oliver K, Innvar S, Lorenc T, *et al.*: **A systematic review of barriers to and facilitators of the use of evidence by policymakers.** *BMC Health Serv Res.* 2014; **14**: 2.
PubMed Abstract | Publisher Full Text | Free Full Text

26. Lavis JN, Boyko JA, Oxman AD, *et al.*: **SUPPORT Tools for evidence-informed health Policymaking (STP) 14: Organising and using policy dialogues to support evidence-informed policymaking.** *Health Res Policy Syst.* 2009; **7**(Suppl 1): S14.
PubMed Abstract | Publisher Full Text | Free Full Text

27. Lomas J: **Using 'linkage and exchange' to move research into policy at a Canadian foundation.** *Health Aff (Millwood).* 2000; **19**(3): 236–40.
PubMed Abstract | Publisher Full Text

28. Lau R, Stevenson F, Ong BN, *et al.*: **Achieving change in primary care--causes of the evidence to practice gap: systematic reviews of reviews.** *Implement Sci.* 2016; **11**: 40.
PubMed Abstract | Publisher Full Text | Free Full Text

29. Wingfield T: **Research protocol for a mixed-methods study to characterise and address the socioeconomic impact of accessing TB diagnosis and care in Nepal.** 2020.
http://www.doi.org/10.17605/OSF.IO/6TC4F

30. Gospodarevskaya E, Tulloch O, Bunga C, *et al.*: **Patient costs during tuberculosis treatment in Bangladesh and Tanzania: the potential of shorter regimens.** *Int J Tuberc Lung Dis.* 2014; **18**(7): 810–17.
PubMed Abstract | Publisher Full Text

31. Gama E, Madan J, Langley I, *et al.*: **Economic evaluation of a shortened standardised treatment regimen of antituberculosis drugs for patients with multidrug-resistant tuberculosis (STREAM): study protocol.** *BMJ Open.* 2016; **6**(10): e014386.
PubMed Abstract | Publisher Full Text | Free Full Text

32. Weaver MS, Lonnroth K, Howard SC, *et al.*: **Interventions to improve adherence to treatment for paediatric tuberculosis in low- and middle-income countries: a systematic review and meta-analysis.** *Bull World Health Organ.* 2015; 93(10): 700–711B.
PubMed Abstract | Publisher Full Text | Free Full Text

33. Moore G: **Process evaluation of complex interventions.** Decipher.Uk.Net. 2014; 19–75.

34. Barter DM, Agboola SO, Murray MB, *et al.*: **Tuberculosis and poverty: the contribution of patient costs in sub-Saharan Africa--a systematic review.** *BMC Public Health.* 2012; **12**: 980.
PubMed Abstract | Publisher Full Text | Free Full Text

35. Thompson SG, Barber JA: **How should cost data in pragmatic randomised trials be analysed?** *BMJ.* 2000; **320**(7243): 1197–1200.
PubMed Abstract | Publisher Full Text | Free Full Text

36. Barber JA, Thompson SG: **Analysis and interpretation of cost data in randomised controlled trials: review of published studies.** *BMJ.* 1998; **317**(7167): 1195–1200.
PubMed Abstract | Publisher Full Text | Free Full Text

For peer review only

For peer review only - http://bmjopen.bmj.com/site/about/guidelines.xhtml

Wellcome Open Research
Wellcome Open Research 2020, 5:19 Last updated: 11 SEP 2020

Open Peer Review

Current Peer Review Status: ✔ ✔

Version 2

Reviewer Report 11 September 2020

https://doi.org/10.21956/wellcomeopenres.17629.r39129

© **2020 Boccia D.** This is an open access peer review report distributed under the terms of the Creative Commons Attribution License, which permits unrestricted use, distribution, and reproduction in any medium, provided the original work is properly cited.

✔ **Delia Boccia**
Faculty of Public Health and Policy, London School of Hygiene and Tropical Medicine, London, UK

I confirm that the authors have appropriately and convincingly addressed all my comments.

Competing Interests: No competing interests were disclosed.

Reviewer Expertise: Social determinants of TB, social protection.

I confirm that I have read this submission and believe that I have an appropriate level of expertise to confirm that it is of an acceptable scientific standard.

Reviewer Report 07 August 2020

https://doi.org/10.21956/wellcomeopenres.17629.r39128

© **2020 Shete P.** This is an open access peer review report distributed under the terms of the Creative Commons Attribution License, which permits unrestricted use, distribution, and reproduction in any medium, provided the original work is properly cited.

✔ **Priya B. Shete**
University of California San Francisco, San Francisco, CA, USA

No further comments. I appreciate the authors attention to my previous suggestions and feel that they have taken care to improve the paper with these details.

Competing Interests: No competing interests were disclosed.

Reviewer Expertise: Implementations science, tuberculosis, social protection.

For peer review only - http://bmjopen.bmj.com/site/about/guidelines.xhtml

I confirm that I have read this submission and believe that I have an appropriate level of expertise to confirm that it is of an acceptable scientific standard.

Version 1

Reviewer Report 26 February 2020

https://doi.org/10.21956/wellcomeopenres.17181.r37807

© **2020 Shete P.** This is an open access peer review report distributed under the terms of the Creative Commons Attribution License, which permits unrestricted use, distribution, and reproduction in any medium, provided the original work is properly cited.

?

Priya B. Shete
University of California San Francisco, San Francisco, CA, USA

The major area for improvement with this protocol would be more discussion in the methods about how the qualitative data will be coded and subsequently used to then generate a menu of feasible options for pilot implementation and evaluation to overcome targeted barriers. I would appreciate further discussion of the conceptual framework that informs the data collection tools/interview guides in Project 2. As Project 2 is particularly important for identifying targeted barriers and facilitators of care for future interventions, I would also like to see what deterministic framework or model is being used to develop those interventions. Any reference to frameworks from implementation science or social epidemiology would perhaps be helpful.

Is the rationale for, and objectives of, the study clearly described?
Yes

Is the study design appropriate for the research question?
Yes

Are sufficient details of the methods provided to allow replication by others?
Partly

Are the datasets clearly presented in a useable and accessible format?
Not applicable

Competing Interests: No competing interests were disclosed.

Reviewer Expertise: Implementations science, tuberculosis, social protection.

I confirm that I have read this submission and believe that I have an appropriate level of expertise to confirm that it is of an acceptable scientific standard, however I have significant reservations, as outlined above.

For peer review only - http://bmjopen.bmj.com/site/about/guidelines.xhtml

Author Response 11 Jun 2020

Tom Wingfield, Liverpool School of Tropical Medicine, UK

We are indebted to Reviewer 2 for these excellent comments, which we have addressed and which we now believe serve to strengthen the protocol. It is important to note that this Wellcome Trust funded study began in 2018 and data collection had already been completed prior to submission of the protocol to Wellcome Open Research. When the research team became aware of Wellcome Open Research, we approached the editorial team who advised that it was still appropriate to submit a study protocol despite data collection being complete. While this is important for critical peer review of the protocol it does, unfortunately, mean that we are unable to adjust parts of the protocol that have been completed and written-up including, for example, the coding strategy.

However, we were able to address Reviewer 2's comments by providing further detail about the coding techniques used and necessary clarification about how the quantitative and qualitative data fed informed outline of the design of the socioeconomic support interventions. In addition, we omitted to include the conceptual framework and logic model that we employed for this research, which we have now rectified and, again, this has helped to improve the protocol and will also be included in subsequent, related publications. Thank you to the Reviewer for their constructive review.

Study activities section: "Project 2 will use and develop mixed methods research techniques [10–15] to conduct a pre-FGD survey, FGDs, and workshop with approximately 40 key stakeholders in Nepal to identify the socioeconomic impact, barriers and facilitators to accessing and engaging with TB diagnosis and care. To inform the design of the FGDs and workshop, we created a conceptual framework for the barriers and facilitators to TB diagnosis and care, which was adapted from a World Health Organisation framework for medication adherence (Figure 3).[30]

(PLEASE SEE FIGURE IN UPDATED PROTOCOL)

Figure 3. Conceptual framework: the barriers and facilitators to TB diagnosis and care.

The central circle, which contains dimensions of medication adherence adapted from the World Health Organization[30], is surrounded by the five main categories of relevant potential interventions. The factors that may promote access to and engagement with TB services (facilitators) are shown in green boxes and factors that may threaten access and engagement (barriers) are shown in white boxes. Therapeutic alliance refers to strong provider-patient relationships. Although Project 2 would gather data on all five dimensions, the psychosocial and social protection dimensions were perceived by the project team to be most pertinent to development of the socioeconomic intervention and were selected for further focus and discussion during Project 2's workshop."

Study activities section: "The final activity in Project 2 will be a one-day workshop bringing together the 40 key stakeholders (see Project 2 Workshop, Extended data) [29] . The morning section of the workshop will consist of interactive presentations from the project team and stakeholder group representatives (including leaders of national social protection schemes

For peer review only - http://bmjopen.bmj.com/site/about/guidelines.xhtml

***in Nepal), and discussions exploring and validating the barriers and opportunities identified during the pre-FGD survey and interviews during FGDs. The afternoon section of the workshop will consist of multi-sectoral working groups (*≤10 diverse stakeholders) developing a shortlist of potential socioeconomic interventions for TB-affected households in Nepal. The shortlist is not intended to consist of defined, unalterable packages, which are immediately ready for trial implementation. Rather, the potential interventions selected are intended to consist of what stakeholders perceive to be key elements or ingredients of psychosocial and economic support for TB-affected households, which are feasible and locally-appropriate to the Nepalese context. The interventions will be presented to the group including strengths, weaknesses, and potential sources of funding for implementation.**

The activities involved in this research form part of a process to develop and evaluate a complex, socioeconomic support intervention. In line with the Medical Research Council's guidance on process evaluation of complex interventions, we developed a logic model to illustrate the developmental stages of the intervention (Figure 4).[31]

(PLEASE SEE FIGURE IN UPDATED PROTOCOL)

Figure 4. Logic model for research in Nepal to develop a locally-appropriate socioeconomic support intervention for TB-affected households.

This Wellcome research will aim to achieve the short-term outcomes described in the logic model. If follow-on funding is successfully obtained, the process to develop and implement the intervention and achieve the long-term outcomes will continue beyond this Wellcome research. This will include adaptation, piloting, and then large-scale randomised trial evaluation of the refined intervention."

Data analysis and statistical plan section: "The Framework method of thematic analysis will be used to manage and analyse data from Project 2 via the NVivo qualitative software package (Version 12) as per published social policy and tuberculosis research [21–24] . Specifically, two researchers (KD and TW) will familiarize themselves with the data through successive reading of transcripts; use both open and closed first order data coding to label data within NVivo; group codes together into a second order codebook of themes and sub-themes. Themes and subthemes will then be further stratified by third order coding to the level at which they predominantly occurred (e.g. individual or household level, health systems level, community level, or governmental level). The primary approach to codes, themes, and sub-themes will be deductive (top down), being informed by our conceptual framework and preliminary analysis of data from Project 1 relating to the social determinants and consequences identified. As coding continues, a secondary approach will integrate inductive (bottom up) coding in order to be iterative, responsive, and flexible as further data becomes available and is collated following each successive FGD."

Competing Interests: None.

Reviewer Report 21 February 2020

https://doi.org/10.21956/wellcomeopenres.17181.r37826

© 2020 Boccia D. This is an open access peer review report distributed under the terms of the Creative Commons Attribution License, which permits unrestricted use, distribution, and reproduction in any medium, provided the original work is properly cited.

? **Delia Boccia**

Faculty of Public Health and Policy, London School of Hygiene and Tropical Medicine, London, UK

This paper describes in detail the protocol of a study aiming at understanding better the social determinants of TB as well as the financial consequences of TB. The study is very relevant: it is epidemiologically sound and the rational for it is clear and strongly supported by the existing literature.

There are two gaps that – in my opinion – should be better filled to make the protocol stronger:

1. The selection of controls for the case-control study. This requires a further dissertation about how practically controls will be selected and how the authors are planning to minimise selection biases. Authors explain why an age-sex matching approach could not be adopted; however, the description of the selection of controls remain elusive which triggers questions like: what sampling strategy will be adopted? How randomness as well as representativeness will be respected? General population controls may be a good option for answering questions around the social determinants of TB; however, I am not convinced they are also the best choice when looking at catastrophic health expenditures. Obviously healthy controls will be better off and wonder whether this second research question could be not better addressed by selecting hospital controls or among people who are ill-affected (albeit not from TB). I suggest the authors to better justify their choices and discuss why alternative options have been dismissed.

2. The qualitative work should include a stronger effort to address not just barriers to access to TB care, but also barriers to existing social protection schemes experienced by TB-patients. It feels like a gigantic missed opportunity if not embedded into the existing plans. If indeed the authors are planning to ascertain existing social protection options for TB patients and access can be maximised, then this should be explicitly stated in the paper.

Is the rationale for, and objectives of, the study clearly described?

Yes

Is the study design appropriate for the research question?

Partly

Are sufficient details of the methods provided to allow replication by others?

Partly

Are the datasets clearly presented in a useable and accessible format?

For peer review only - http://bmjopen.bmj.com/site/about/guidelines.xhtml

Not applicable

Competing Interests: No competing interests were disclosed.

Reviewer Expertise: Social determinants of TB, social protection.

I confirm that I have read this submission and believe that I have an appropriate level of expertise to confirm that it is of an acceptable scientific standard, however I have significant reservations, as outlined above.

Author Response 11 Jun 2020

Tom Wingfield, Liverpool School of Tropical Medicine, UK

1. The selection of controls for the case-control study. This requires a further dissertation about how practically controls will be selected and how the authors are planning to minimise selection biases. Authors explain why an age-sex matching approach could not be adopted; however, the description of the selection of controls remain elusive which triggers questions like: what sampling strategy will be adopted? How randomness as well as representativeness will be respected? General population controls may be a good option for answering questions around the social determinants of TB; however, I am not convinced they are also the best choice when looking at catastrophic health expenditures. Obviously healthy controls will be better off and wonder whether this second research question could be not better addressed by selecting hospital controls or among people who are ill-affected (albeit not from TB). I suggest the authors to better justify their choices and discuss why alternative options have been dismissed.

Author response: Thank you for raising this important issue with which we wholeheartedly agree. It is important to note that this Wellcome Trust funded study began in 2018 and data collection had already been completed prior to submission of the protocol to Wellcome Open Research. When the research team became aware of Wellcome Open Research, we approached the editorial team who advised that it was still appropriate to submit a study protocol despite data collection being complete. While this is important for critical peer review of the protocol it does, unfortunately, mean that we are unable to adjust the protocol including, for example, sampling strategies at this stage. However, we have attempted to respond to the reviewer's comments about sampling strategy and control population randomness and representativeness. We have also made it clearer that the data from controls will only be used to characterise social determinants of TB and there will be no analysis comparing health expenditure in controls vs people with TB, which we agree would be inappropriate with general population controls.

Study population section: "Concurrently, 100 people without TB from the study sites (controls) will be invited to participate with written, informed consent (see Project 1 Interview Healthy Control Consent Form, Extended data)[29]. Due to constraints in study budget, duration, and field logistics, it will not be possible to age and sex match cases and controls nor to randomly select controls using geospatial or other household randomisation techniques. Therefore, a convenience sampling strategy was used. In order to be as widely representative of and generalisable to the background population as

possible, following project team and community mobiliser discussions in the study sites, we opted to recruit control participants from diverse locations within the districts, which were attended by a broad demographic cross-section of the local population in terms of age, gender, and socioeconomic position. These diverse study site locations include: tea houses, primary healthcare centres, antenatal and immunization clinics, door-to-door visits following sputum camps (e.g. people who tested negative for TB), and public gathering places. Interviewers will visit the control recruitment locations at similar times of the morning and aim to consecutively recruit all individuals in attendance at that location. Similar numbers of participants from each recruitment location will be recruited until the sample size of 100 people without TB is reached. We acknowledge that convenience sampling may be associated with a higher likelihood of a non-generalisable control cohort than other techniques. To try to address this, the survey used to collect data from the controls has incorporated multiple questions concerning sociodemographic variables, including education level, occupation, amenities, and assets, from the most recent version of the Nepal Household Survey, which is publicly available. Descriptive analysis will evaluate whether the controls recruited are representative of the wider population in those districts through comparison of their sociodemographic data with respondents to the national survey in the same districts. Any differences between the control population and background population will then be highlighted transparently in corresponding research outputs and publications."

"Data analysis and statistical plan section: "Social determinants of TB including sociodemographic characteristics, socioeconomic position, stigma and social capital levels, and TB-related knowledge will be compared between ACF patients, PCF patients, and controls using Chi-squared test, Pearson's test, one-way ANOVA, and multiple logistic regression models where appropriate. No comparison will be made between patients and controls concerning healthcare expenditure as this data was not collected from controls."

2. The qualitative work should include a stronger effort to address not just barriers to access to TB care, but also barriers to existing social protection schemes experienced by TB-patients. It feels like a gigantic missed opportunity if not embedded into the existing plans. If indeed the authors are planning to ascertain existing social protection options for TB patients and access can be maximised, then this should be explicitly stated in the paper.

Author Response: We agree entirely with the reviewer and are grateful for this apposite point. Within the confines of not being able to alter the protocol given that data collection is now completed, we have clarified this important issue in the protocol to explain that, across Projects 1 and 2, quantitative and qualitative data concerning social protection in Nepal was gathered, including the coverage, target recipients, barriers to access, and limitations of existing packages.

Study activities section:** "**The interviews will be structured (see Project 1 Interview, Extended data) [29] and gather data on: i) socioeconomic position, evaluated by a multi-dimensional poverty score [6, 9, 13, 15] assessing dwelling characteristics, assets, and access to amenities; ii) household structure, including distribution of age, sex, and employment of household members; iii) food expenditure and security; iv) costs of engaging with TB care including direct costs (e.g. medicines, clinic visits, food, and travel) and indirect costs (e.g.

lost income), which will be evaluated using an adapted version of WHO's TB Patient Costs Survey integrated into the interview [22, 23] ; v) coping strategies including dissaving (e.g. selling assets), schooldays lost, and temporary income-generating activities; vi) TB-related knowledge including understanding of transmission, prevention, and treatment of TB; vii) psychosocial situation evaluated through questions relating to social capital, quality of life, and stigma (Controls without TB will not be asked any questions about the impact that having TB disease has on their psychosocial situation); and viii) access and uptake of existing social protection schemes (whether TB-specific or TB-inclusive) and support for TB-affected households, evaluated through use of both closed ranking and open free-text questions to establish what socioeconomic and other support people with TB and their households receive or would like to receive."

Study activities section: "A short individual pre-FGD survey (see Project 2 Survey, Extended data) [29] will be provided to participants prior to the initiation of an FGD. The survey will detail: participants' demographics; the stakeholder group to which the participant belongs; and their opinions on community, patient, health system, existing social protection schemes, and wider obstacles to achieving successful TB treatment outcomes.

The FGDs will be semi-structured and incorporate open-ended questions concerning barriers and facilitators to accessing and engaging with TB care in Nepal, and existing platforms and potential opportunities to mitigate these barriers including social protection schemes (see Project 2 Focus Groups, Extended data) [29] .

Study activities section: "The final activity in Project 2 will be a one-day workshop bringing together the 40 key stakeholders (see Project 2 Workshop, Extended data) [29] . The morning section of the workshop will consist of interactive presentations from the project team and stakeholder group representatives (including leaders of national social protection schemes in Nepal), and discussions exploring and validating the barriers and opportunities identified during the pre-FGD survey and interviews during FGDs."

Competing Interests: None.

For peer review only - http://bmjopen.bmj.com/site/about/guidelines.xhtml

COREQ (COnsolidated criteria for REporting Qualitative research) Checklist

A checklist of items that should be included in reports of qualitative research. You must report the page number in your manuscript where you consider each of the items listed in this checklist. If you have not included this information, either revise your manuscript accordingly before submitting or note N/A.

Topic	Item No.	Guide Questions/Description	Reported on Page No.
**Domain 1: Research team and reflexivity**			
Personal characteristics			
Interviewer/facilitator	1	Which author/s conducted the interview or focus group?	
Credentials	2	What were the researcher's credentials? E.g. PhD, MD	
Occupation	3	What was their occupation at the time of the study?	
Gender	4	Was the researcher male or female?	
Experience and training	5	What experience or training did the researcher have?	
Relationship with participants			
Relationship established	6	Was a relationship established prior to study commencement?	
Participant knowledge of the interviewer	7	What did the participants know about the researcher? e.g. personal goals, reasons for doing the research	
Interviewer characteristics	8	What characteristics were reported about the inter viewer/facilitator? e.g. Bias, assumptions, reasons and interests in the research topic	
**Domain 2: Study design**			
Theoretical framework			
Methodological orientation and Theory	9	What methodological orientation was stated to underpin the study? e.g. grounded theory, discourse analysis, ethnography, phenomenology, content analysis	
Participant selection			
Sampling	10	How were participants selected? e.g. purposive, convenience, consecutive, snowball	
Method of approach	11	How were participants approached? e.g. face-to-face, telephone, mail, email	
Sample size	12	How many participants were in the study?	
Non-participation	13	How many people refused to participate or dropped out? Reasons?	
Setting			
Setting of data collection	14	Where was the data collected? e.g. home, clinic, workplace	
Presence of non-participants	15	Was anyone else present besides the participants and researchers?	
Description of sample	16	What are the important characteristics of the sample? e.g. demographic data, date	
Data collection			
Interview guide	17	Were questions, prompts, guides provided by the authors? Was it pilot tested?	
Repeat interviews	18	Were repeat inter views carried out? If yes, how many?	
Audio/visual recording	19	Did the research use audio or visual recording to collect the data?	
Field notes	20	Were field notes made during and/or after the inter view or focus group?	
Duration	21	What was the duration of the inter views or focus group?	
Data saturation	22	Was data saturation discussed?	
Transcripts returned	23	Were transcripts returned to participants for comment and/or	

For peer review only

Topic	Item No.	Guide Questions/Description	Reported on Page No.
		correction?	
**Domain 3: analysis and findings**			
Data analysis			
Number of data coders	24	How many data coders coded the data?	
Description of the coding tree	25	Did authors provide a description of the coding tree?	
Derivation of themes	26	Were themes identified in advance or derived from the data?	
Software	27	What software, if applicable, was used to manage the data?	
Participant checking	28	Did participants provide feedback on the findings?	
Reporting			
Quotations presented	29	Were participant quotations presented to illustrate the themes/findings? Was each quotation identified? e.g. participant number	
Data and findings consistent	30	Was there consistency between the data presented and the findings?	
Clarity of major themes	31	Were major themes clearly presented in the findings?	
Clarity of minor themes	32	Is there a description of diverse cases or discussion of minor themes?	

Developed from: Tong A, Sainsbury P, Craig J. Consolidated criteria for reporting qualitative research (COREQ): a 32-item checklist for interviews and focus groups. *International Journal for Quality in Health Care*. 2007. Volume 19, Number 6: pp. 349 – 357

Once you have completed this checklist, please save a copy and upload it as part of your submission. DO NOT include this checklist as part of the main manuscript document. It must be uploaded as a separate file.

For peer review only

BMJ Open

Barriers and facilitators to accessing tuberculosis care in Nepal: a qualitative study to inform the design of a socioeconomic support intervention

Journal:	*BMJ Open*
Manuscript ID	bmjopen-2021-049900.R1
Article Type:	Original research
Date Submitted by the Author:	16-Jun-2021
Complete List of Authors:	Dixit, Kritika; BNMT; Karolinska Institute, Department of Global Public Health. WHO Collaborating Centre on Tuberculosis and Social Medicine<br>Biermann, Olivia; Karolinska Institute, Department of Global Public Health. WHO Collaborating Centre on Tuberculosis and Social Medicine<br>Rai, Bhola; BNMT<br>Aryal, Tara ; BNMT<br>Mishra, Gokul; BNMT; Liverpool School of Tropical Medicine<br>Teixeira de Siqueira-Filha, Noemia; Liverpool School of Tropical Medicine; University of York<br>Paudel, Puskar; BNMT; KNCV Tuberculosis Foundation<br>Pandit, Ram ; BNMT<br>Sah, Manoj; BNMT<br>Majhi, Govinda ; BNMT<br>Levy, Jens; KNCV<br>Rest, Job; KNCV<br>Gurung, Suman; BNMT; Liverpool School of Tropical Medicine,<br>Dhital, Raghu; BNMT<br>Lönnroth, Knut; Karolinska Institute, Department of Global Public Health. WHO Collaborating Centre on Tuberculosis and Social Medicine<br>Squire, Bertie; Liverpool School of Tropical Medicine; Royal Liverpool Hospital, Respiratory Medicine<br>Caws, Maxine; BNMT; Liverpool School of Tropical Medicine<br>Sidney, Kristi; Karolinska Institute, Department of Global Public Health. WHO Collaborating Centre on Tuberculosis and Social Medicine<br>Wingfield, Tom; Liverpool School of Tropical Medicine, Department of Global Public Health. WHO Collaborating Centre on Tuberculosis and Social Medicine; Karolinska Institute
**Primary Subject Heading**:	Infectious diseases
Secondary Subject Heading:	Qualitative research
Keywords:	Tuberculosis < INFECTIOUS DISEASES, PUBLIC HEALTH, QUALITATIVE RESEARCH

I, the Submitting Author has the right to grant and does grant on behalf of all authors of the Work (as defined in the below author licence), an exclusive licence and/or a non-exclusive licence for contributions from authors who are: i) UK Crown employees; ii) where BMJ has agreed a CC-BY licence shall apply, and/or iii) in accordance with the terms applicable for US Federal Government officers or employees acting as part of their official duties; on a worldwide, perpetual, irrevocable, royalty-free basis to BMJ Publishing Group Ltd ("BMJ") its licensees and where the relevant Journal is co-owned by BMJ to the co-owners of the Journal, to publish the Work in this journal and any other BMJ products and to exploit all rights, as set out in our licence.

The Submitting Author accepts and understands that any supply made under these terms is made by BMJ to the Submitting Author unless you are acting as an employee on behalf of your employer or a postgraduate student of an affiliated institution which is paying any applicable article publishing charge ("APC") for Open Access articles. Where the Submitting Author wishes to make the Work available on an Open Access basis (and intends to pay the relevant APC), the terms of reuse of such Open Access shall be governed by a Creative Commons licence – details of these licences and which Creative Commons licence will apply to this Work are set out in our licence referred to above.

Other than as permitted in any relevant BMJ Author's Self Archiving Policies, I confirm this Work has not been accepted for publication elsewhere, is not being considered for publication elsewhere and does not duplicate material already published. I confirm all authors consent to publication of this Work and authorise the granting of this licence.

For peer review only

Barriers and facilitators to accessing tuberculosis care in Nepal: a qualitative study to inform the design of a socioeconomic support intervention

Authors

Kritika Dixit,[1,2] Olivia Biermann,[2] Bhola Rai,[1] Tara Prasad Aryal,[1] Gokul Mishra,[1,3] Noemia Teixeira de Siqueira-Filha,[3,4] Puskar Raj Paudel, [1,5] Ram Narayan Pandit,[1] Manoj Kumar Sah,[1] Govinda Majhi, [1] Jens W. Levy,[5] Job van Rest,[5] Suman Chandra Gurung,[1,3] Raghu Dhital,[1] Knut Lönnroth,[2] S Bertel Squire,[3] Maxine Caws,[1,3] *Kristi Sidney Annerstedt,[2] *Tom Wingfield [2,6]

Affiliations

[1] Birat Nepal Medical Trust, Lazimpat-2, Kathmandu, Nepal. Tel: +9774436434

[2] Department of Global Public Health, WHO Collaborating Centre on Tuberculosis and Social Medicine, Karolinska Institutet, 17177 Solna, Sweden

[3] Department of Clinical Sciences, Liverpool School of Tropical Medicine, Pembroke Place, Liverpool L3 5QA, UK

[4] Department of Health Sciences, University of York, Heslington York, YO10 5DD, UK

[5] KNCV Tuberculosis Foundation, 2514 JD The Hague, The Netherlands

[6] Departments of International Public Health and Clinical Sciences, Liverpool School of Tropical Medicine, Pembroke Place, Liverpool, L3 5QA, UK.

*Joint Final Authors

Corresponding Author

Dr Tom Wingfield, Senior Clinical Lecturer and Honorary Consultant Physician, Room G216, LIV-TB Collaboration, Departments of International Public Health and Clinical Sciences, Liverpool School of Tropical Medicine, Pembroke Place, Liverpool, L3 5QA, UK. Tel: +441517053736. Email: tom.wingfield@lstmed.ac.uk

For peer review only

ABSTRACT

Objective

Psychosocial and economic (socioeconomic) barriers, including poverty, stigma, and catastrophic costs, impede access to tuberculosis (TB) services in low-income countries. We aimed to characterise the socioeconomic barriers and facilitators of accessing TB services in Nepal to inform the design of a locally-appropriate socioeconomic support intervention for TB affected households.

Design

From August 2018 to July 2019, we conducted an exploratory qualitative study consisting of semi-structured focus group discussions (FGDs) with purposively-selected multi-sectoral stakeholders. The data was managed in NVivo 12, coded by consensus, and analysed thematically.

Setting

The study was conducted in four districts, Makwanpur, Chitwan, Dhanusha and Mahottari, which have a high prevalence of poverty and TB.

Participants

Seven FGDs were conducted with 54 in-country stakeholders, grouped by stakeholders, including people with TB (n=21), community stakeholders (n=13), and multidisciplinary TB healthcare professionals (n=20) from the National TB Program.

Results

The perceived socioeconomic barriers to accessing TB services were: inadequate TB knowledge and advocacy; high food and transportation costs; income loss; and stigma. The perceived facilitators to accessing TB care and services were: enhanced championing and awareness-raising about TB and TB services; social protection including health insurance; cash, vouchers and/or nutritional allowance to cover food and travel costs; and psychosocial support and counselling integrated with existing adherence counseling from the National TB Program.

Conclusion

These results suggest that support interventions that integrate TB education, psychosocial counselling, and expand on existing cash transfer schemes would be locally-appropriate and could address the socioeconomic barriers to accessing and engaging with TB services faced by TB-affected households in Nepal. The findings have been used to inform the design of a

For peer review only

socioeconomic support intervention for TB-affected households. The acceptability, feasibility, and impact of this intervention on TB-related costs, stigma and TB treatment outcomes, is now being evaluated in a pilot implementation study in Nepal.

Keywords

Tuberculosis; barriers and facilitators; socioeconomic support; healthcare access; Nepal

For peer review only

For peer review only - http://bmjopen.bmj.com/site/about/guidelines.xhtml

Strengths and limitations

- The focus group discussions contributed to new knowledge on optimal local strategies to mitigate the socioeconomic impact of TB.

- The evidence has directly informed the design of a novel socioeconomic support intervention for TB-affected households, which is undergoing pilot evaluation in Nepal.

- The credibility and trustworthiness of the study was maintained through member checking, using multiple coders, conducting a consensus-based coding, recruiting local interviewers for data collection, performing triangulation and including a broad selection of multidisciplinary stakeholders to inform the study conclusion.

- The study was conducted in four districts of Nepal, mostly lowland "plains" districts, which could affect the transferability of the findings.

- People who were diagnosed with TB in private sectors or those lost to follow up did not participate in the study despite, in other settings, having been shown to be groups at high risk of severe socioeconomic impact of TB.

For peer review only

BACKGROUND

Tuberculosis (TB) kills 1.3 million people each year worldwide, more than any other single infectious disease including, up to the time of writing, Covid-19.[1] In 2019, an estimated 10 million became ill with TB, of whom 2.9 million were not notified or remained undiagnosed and untreated.[1] In low- and middle-income countries (LMICs), stigma, marginalization, and catastrophic costs of accessing TB diagnosis and care, coupled with limited social protection coverage, can delay diagnosis, decrease TB treatment success rates, and push TB-affected households into further impoverishment.[2,3] To address this and move towards TB elimination, the World Health Organization's (WHO) 2015 End TB Strategy set the bold target that "Zero TB-affected families should face catastrophic costs" and that psychosocial and economic (socioeconomic) support should be provided to TB-affected people.[4]

Nepal is a lower-middle-income country in South Asia with significant TB incidence (annual incidence 245/100,000) and mortality.[5] Despite free basic TB diagnostic tests, medicines and financial support for people with drug-resistant (DR-TB), approximately one in two people with TB face catastrophic costs (defined as the total TB-related costs equivalent to greater than 20% of a household's annual income) while accessing TB care in Nepal.[6,7] Such costs include travel for Directly Observed Treatment Short-course (DOTS), additional food expenditure, and lost income, which can contribute to adverse TB treatment outcomes, especially for the poorest, most vulnerable households.[6,8–10]

The Nepal National TB Program (NTP) provides 3,000 Nepalese Rupees (~27 USD) in cash incentives monthly for transportation and nutritional support to people with multi-drug resistant TB (MDR-TB)[11] who are enrolled in government treatment centers and receiving ambulatory care. There is currently no cash incentive scheme for people with drug-sensitive TB (DS-TB) in Nepal.[12] In other settings, socioeconomic support for TB-affected households, including mutual support groups and cash transfers, has been shown to help overcome barriers to accessing TB services, defraying catastrophic costs, and improving treatment success rates.[3,13–16] However, there is limited context-specific understanding of the barriers and facilitators to TB diagnosis and care in

For peer review only - http://bmjopen.bmj.com/site/about/guidelines.xhtml

LMICs with which to inform the development of tailored socioeconomic support interventions for people with TB and their households.[1] This study aimed to address this knowledge gap in Nepal.

MATERIALS and METHODS

Study design: We conducted an exploratory qualitative study, which used semi-structured focus group discussions (FGDs) to collate the perceptions of key stakeholders in Nepal regarding socioeconomic barriers and facilitators of accessing and engaging with TB diagnosis and care. The study formed part of a larger programme of mixed-methods research[17] to design a locally-appropriate socioeconomic support intervention for TB-affected households. The study adhered to the COnsolidated criteria for REporting Qualitative research (COREQ) Checklist (Research Checklist).[18]

Study setting: The study was conducted in four districts of Nepal where Birat Nepal Medical Trust (BNMT), a Nepalese organization with a focus on TB-related implementation research, implemented IMPACT-TB project. The districts: Makwanpur, Chitwan, Dhanusha, and Mahottari have a high prevalence of poverty and TB (Figure 1).[11] Makwanpur is a hilly district with limited road networks. Other three districts are lowland plains and challenged by high population density, poor health indicators, and high rates of illiteracy.

Figure 1. Map of population and TB case notification rates in the four study districts

(Authors suggest to insert the Figure 1 here in this section)

Sampling: A desk-based scoping exercise was initially performed by team members (KD, RD, TW) to identify participants from relevant stakeholder groups in Nepal. To collate diverse perspectives on barriers and facilitators of TB diagnosis and treatment, the team purposively selected participants who had direct or indirect experiences with TB services. The participants included: people affected by TB who were currently receiving or had recently completed DS-TB or MDR-TB treatment with the NTP (referred to as "people with TB" in the study); community leaders such as female community health volunteers, teachers, and social leaders from civil society organisations (CSOs, referred to as "community stakeholders"); and TB healthcare

For peer review only - http://bmjopen.bmj.com/site/about/guidelines.xhtml

professionals, including those working with the NTP, community volunteers, and TB-focused NGOs (referred to as “NTP stakeholders”) [Table 1]. The list of people with TB, including their demographics were gathered from the IMPACT TB database or registers of the health clinics in each district. Community stakeholders were community leaders or those working in civil society and were selected based on their in-depth knowledge on the locak context and experiences of working with the communities, preferable in health progams. TB healthcare professionals, such as those working with the NTP or TB-focused NGOs, have several years’ experience in planning, designing, and implementing NTP activities. Community volunteers or mobilizers were people working with the IMPACT-TB project, who have first-hand experiences in screening symptoms of TB and supporting people with TB to adhere to and complete their treatment. These participants were selected based on the expertise in delivering community programs and to bring diverse perception of the stakeholders into the study. Using telephone, email, or in-person meetings, we invited 55 individuals to participate in the study. Inclusion criteria were being 18 years of age or older and being able to give informed consent. Seven participants were invited to each of the seven FGDs with the exception of the TB healthcare professional FGD, which consisted of 12 participants. This related to the logistical challenges of organizing more than one FGD with this group due to their working hours and time constraints coupled with the aim of representation from the public, private, and NGO sectors of TB healthcare.

Data collection: The study team consisted of diverse members from multiple sectors including a physician, senior TB researchers, social scientists, public health professionals, and project managers. An interview guide was developed by the coauthors with previous qualitative methods experience: TW (Male, Principal Investigator, TB researcher), KD (Female, doctoral student, Project Manager), and BR (Male, Public Health Specialist, Research Associate); BR and KD are employed by BNMT. The interview guide consisted of open-ended questions to explore the perceptions of participants concerning protective factors and risk factors for exposure to TB and development of TB disease; barriers and facilitators to accessing and engaging with TB diagnosis

For peer review only - http://bmjopen.bmj.com/site/about/guidelines.xhtml

and care, including the recommendations for better access and engagement with TB diagnosis and care; and the socio-economic impact on people with TB of being ill with the disease.

Prior to conducting FGDs, participants were provided with a '*Participant Information Sheet'* that explained the purpose of the study, benefit and harm, and confidentiality.[17] Participants were provided time as they would require to read and understand the information in the paper and then decide if they are willing to participate in the study. The FGDs were conducted in a local hotel accessible to participants in the study districts. The topic guide was piloted with a group of seven female and male participants with TB resulting in minor refinements to the FGD structure and delivery techniques. TW moderated the FGD with TB healthcare professional and KD and BR moderated the other six FGDs. Apart from these researchers, district field staff who supported patients attended the discussions and facilitated any dialectic interpretation or contextual explanations related to access to and engagement with TB services.

We conducted seven FGDs with 54 participants, which the project team perceived as giving sufficient information power for the study.[19] Of the participants, three quarters were male and the average age was 42 years (Table 1). To encourage an environment in which participants felt comfortable and able to share their opinions and to balance gender representation, two of these FGDs were specifically for females with DS-TB and female TB community mobilizers. In all the FGDs, there were seven participants, except for the FGD with community leaders (n=6), FGD with TB healthcare professionals (n=12) and FGD with community mobilizers (n=8). One invitee from the community leader's FGD declined to participate due to lack of time. One additional community mobilizer showed interest to participate in the FGD with community mobilizers' and was also included. We did not conduct any follow-up discussions with participants but some of the participants attended a workshop to discuss the FGD findings, the outputs of which are published elsewhere.[12] We performed real-time member checking in each FGD by noting key points of the discussion, summarizing them on a wall chart, and clarifying their accuracy with the group. No formal field notes were taken. The FGDs, which lasted 90-120 minutes, were all conducted in Nepali language apart from the FGD with TB healthcare professionals, which was

For peer review only - http://bmjopen.bmj.com/site/about/guidelines.xhtml

conducted in English. FGDs were audio recorded, translated into English from Nepali language, and back-translated by an independent translator who was not part of the project team. Each FGD was concluded when the facilitators collectively felt the topics in the FGD interview guide were sufficiently explored.

Table 1: List of FGD stakeholder groups and participants

Stakeholder group	Sex	Age group (years)	District	Total number of participants
People diagnosed with TB, mixed sex group	Female	Under 20	Makwanpur	7
	Male	25-30	Mahottari	
	Male	45-50	Makwanpur	
	Male	20-25	Dhanusha	
	Female	Under 20	Makwanpur	
	Male	30-35	Chitwan	
	Male	55-60	Chitwan	
People diagnosed with MDR-TB	Male	40-45	Chitwan	7
	Male	70-75	Chitwan	
	Male	20-25	Chitwan	
	Male	20-25	Chitwan	
	Male	45-50	Chitwan	
	Male	45-50	Chitwan	
	Female	20-25	Chitwan	
Females diagnosed with TB	Female	60-65	Mahottari	7
	Female	25-30	Makwanpur	
	Female	40-45	Mahottari	
	Female	45-50	Chitwan	
	Female	45-50	Dhanusha	
	Female	25-30	Dhanusha	
	Female	25-30	Makwanpur	
Community Leaders	Female	50-55	Chitwan	6
	Male	45-50	Mahottari	
	Male	35-40	Makwanpur	
	Male	45-50	Chitwan	

For peer review only

	Female	50-55	Chitwan	
	Male	40-45	Dhanusha	
Civil Society Organization	Male	35-40	Chitwan	7
	Male	40-45	Chitwan	
	Male	65-70	Chitwan	
	Male	25-30	Mahottari	
	Male	45-50	Chitwan	
	Male	45-50	Makwanpur	
	Male	25-30	Dhanusha	
TB Healthcare Professionals	Male	55-60	Kathmandu	12
	Male	30-35	Kathmandu	
	Male	30-35	Kathmandu	
	Male	30-35	Kathmandu	
	Female	25-30	Kathmandu	
	Male	55-60	Kathmandu	
	Male	45-50	Kathmandu	
	Male	45-50	Kathmandu	
	Male	55-60	Kathmandu	
	Male	55-60	Kathmandu	
	Male	55-60	Kathmandu	
	Male	45-50	Kathmandu	
Community Mobilizers	Male	45-50	Dhanusha	8
	Female	30-35	Chitwan	
	Female	30-35	Makwanpur	
	Male	25-30	Dhanusha	
	Male	30-35	Chitwan	
	Male	40-45	Mahottari	
	Male	25-30	Mahottari	
	Female	20-25	Makwanpur	
Total	Male: 38<br>Female: 16			54

Analysis: We applied thematic analysis using NVivo 12 to manage the data.[20] The study used multiple coders, KD and TW, who familiarised themselves with the data through successive reading of transcripts. KD and TW separately generated the initial codes for each transcript before discussing and comparing the perception of understanding of the codes. The codes were updated through regular discussion as further data became available and collated following each successive FGD. To increase trustworthiness of the study, after all the transcripts were coded and analysed, KD and TW independently reviewed coding and themes and refined them through further discussion, triangulation, and consensus where necessary.[21] Both open and closed first-order categories were used to label data. Categories were then grouped into second-order and third-order themes (Supplementary file I). Table 2 shows an example of the analysis process of

For peer review only - http://bmjopen.bmj.com/site/about/guidelines.xhtml

codes and themes. To better inform the design and delivery of the socioeconomic intervention for TB-affected households within the wider context of health services delivery, the first-order themes were then mapped to four levels of an adapted WHO Treatment Adherence Framework: I. TB, health, and basic education; II. Social protection and nutrition; III. Psychosocial; IV. Healthcare system, TB diagnosis and care delivery.[22] We chose to structure our analysis on the themes mapped to levels I-III of the WHO Framework because these levels were the most relevant to the study's aim of informing design and development of a socioeconomic support intervention for TB-affected households. While important, themes identified that mapped to category IV of the Framework, such as governmental policy, political commitment, public-private mix, and healthcare infrastructure were perceived by the study team to be largely unmodifiable by a household-level socioeconomic support intervention. These themes are reported under health system categories and are shown in Supplementary File II.

Table 2: An example of coding from the FGDs

FGD	Quote	First-order category*	Second-order Themes	Third-order themes
People diagnosed with TB	FGD with people diagnosed with TB, 30-35 years age group, male: *"People get criticized for having TB. The community perceives a TB patient isn't the same as a normal person…. due to*	Psychosocial	- Enacted stigma<br>- Perceived stigma<br>- Lack of knowledge	Stigma as social barrier to access

For peer review only - http://bmjopen.bmj.com/site/about/guidelines.xhtml

	lack of awareness."			
* Adapted from a World Health Organization Medication Adherence Framework[22]				

The study protocol is provided in the Supplementary file III. The study received ethical approvals from the University of Liverpool (No. 2436) and the Nepal Health Research Council (208/2018). Written consent was obtained from all participants. Confidentiality of the participants was maintained by anonymizing FGD responses, keeping any paper data in a locked cabinet at BNMT's office, and securing the data in a password-protected database.

Patient and public involvement

Patient and/ or the public were not involved in the design or conduct of this research.

RESULTS

Overall, 36 codes related to eight themes were identified [Supplementary File I]. Below, we focus only on the perceived barriers and facilitators that mapped to categories I. TB, health, and basic education, II. Social protection, and nutrition III. Psychosocial, of the adapted WHO Framework. These barriers and facilitators are shown in Figure 2.

TB, health, and basic education

Theme: Information barrier to access and adhere to TB diagnosis and treatment

Across FGDs, we identified low literacy and education about TB as a barrier to accessing TB diagnosis and engaging with TB treatment (Figure 2). Knowledge about TB including transmission, prevention, symptoms, how and where to get diagnosed, treatment regimens and duration, and the TB services available at health facilities were perceived to be limited, especially among illiterate and underserved groups or rural populations. A female participant with TB said: *"I didn't know that TB medicines were free in hospitals. I knew it only when I visited the health post."*

For peer review only - http://bmjopen.bmj.com/site/about/guidelines.xhtml

The FGD with community stakeholders suggested that this limited knowledge about TB negatively impacted engagement with TB services and treatment adherence. This was noted to potentially increase the likelihood of a delayed diagnosis, more advanced disease at presentation, and acquisition of DR-TB during treatment. The perceived lack of knowledge was predominantly felt to relate to suboptimal TB education, advocacy, and political commitment from health system and governmental leaders. Participants also reported that the current health education programs are scarce and unable to reach poorer, educationally and socially marginalized communities with high TB risk.

Theme: Facilitating treatment access through education about TB disease and advocacy about TB services, especially in remote communities

Across all the FGDs, participants described the need to raise community awareness on risk factors for TB, mode of TB transmission, and TB signs and symptoms. Community stakeholders particularly felt that future awareness-raising programs would benefit by informing communities about available free TB diagnostic and treatment services in their local area and adding a component to reduce TB-related stigma. Further, NTP stakeholders stressed the importance of not only providing education but also, in the face of competing health beliefs, influencing attitudes, and promoting behaviour change.

Community stakeholders suggested that the government should take responsibility for the development and implementation of intensive household- and village-level awareness programs using technology such as smartphone applications and social media. They also recommended broadcasting educational campaigns on television adapted from similar campaigns in the field of HIV (*HIV ka sawal)* and maternal care (*Aama ko Maya)* in Nepal. However, there was concern that information transmitted through media could bypass vulnerable, poor, and marginalized populations. To overcome this, the group suggested innovative and interactive community-based approaches such as coordination with local women's group-initiated self-help enterprises, street plays, and engaging TB survivors as peer champions and educators to improve TB education.

For peer review only - http://bmjopen.bmj.com/site/about/guidelines.xhtml

The involvement and ownership of TB education programs by community leaders, including volunteers, teachers, and community mobilizers, was also deemed important to achieve effective, decentralized delivery. Furthermore, the stakeholders acknowledged the essential role of healthcare provider-led education as part of a client-provider contract because people with TB need - and will follow - advice from healthcare providers only when that advice is relayed sensitively and understood thoroughly. Nevertheless, people with TB indicated that both sensitization and education from a trusted source were key to deliver education successfully and to enable and empower communities.

> **FGD with community leaders, 50-55 years age group, female:** *"We need to create [educational] groups attached to health centres and schools. Community and locally-elected leaders and teachers could give education to their communities and conduct TB awareness training and workshops."*

II. Social protection and nutrition

Theme: Social and economic barriers to accessing and engaging with TB diagnosis and treatment

Across FGDs, the participants reported food insecurity, high travel and food costs, and lost income as key barriers to accessing and engaging with TB diagnosis and treatment (Figure 2). The direct out-of-pocket costs of seeking TB diagnosis and engaging with DOTS at both public and private clinics, including food and transport, were raised repeatedly across FGDs as a significant barrier to timely diagnosis and medication adherence.

> **FGD with community leaders, 50-55 years age group, female:** *"TB medicines are free but people also need money for two-way transportation and food. TB illness [and even] TB treatment can make people weak and nutrition is needed. How can people afford these [nutrition and transportation] costs?"*

Nonetheless, patient journeys were repeatedly reported as long and convoluted, including a public-private (PPM) mix of traditional medicine, pharmacies, local private healthcare providers,

For peer review only - http://bmjopen.bmj.com/site/about/guidelines.xhtml

and larger private clinics before reaching NTP diagnostic and treatment services (Figure 2). As a result, TB affected households incurred significant expenses.

FGD with people diagnosed with TB, 45-50 years age group, male: *"I visited all the pharmacies in my city, about 15-17 medicals [pharmacies] overall. I used to buy pneumonia medicine and take it every time. TB was not initially identified. After a month without taking any medicines, TB was identified [at the government clinic]….. The barrier is more financial. I spent approximately 1.7 lakhs [~1530 USD] for my treatment."*

Undernutrition and food insecurity were recognized as risk factors for TB and to contribute to suboptimal adherence. Across FGDs, it was noted that many TB-affected households have insufficient resources to buy nutritious food. In addition, it was perceived that lack of education negatively impacted decision-making related to nutrition to recover during TB treatment. In particular, community leaders and people with DR-TB raised concerns that the current monthly governmental nutrition allowance was insufficient to obtain nutritional food, such as meat and fruits, which is often recommended by healthcare providers. Female participants with TB mentioned a trade-off between out-of-pocket expenses to purchase the recommended nutritional food versus transportation expenses associated with DOTS at TB clinics.

Lost income was identified in the majority of FGDs as a notable barrier to accessing diagnosis and engaging with TB care, which compounded the economic impact of out-of-pocket travel and food costs. People with TB expressed guilt and stress related to having to stop work following their TB diagnosis and the fear of not getting employment again. Female participants described the impact as hardest on poorer populations, women working in the fields, and labourers, who lack the free time required to go to the health facility for daily DOTS, especially when opening hours and prime labouring hours coincide.

Community stakeholders and people with TB frequently cited that TB-affected households use coping strategies to mitigate the economic impact of TB, most commonly to obtain funds to buy food. Selling assets, such as livestock, milk, land, and jewelry, was reported as a predominant

For peer review only - http://bmjopen.bmj.com/site/about/guidelines.xhtml

coping strategy. However, it was noted that some vulnerable patients were so poor that they have nothing to sell. Other coping strategies mentioned included borrowing money, formally and informally, which led to further economic hardship and difficulties maintaining adherence.

> **FGD with people diagnosed with TB, 25-30 years age group, male:** *"I had difficulties [to pay money to access TB services] and wasn't able to go [to the clinic] for a month. I had to borrow money from my friends."*

Theme: Facilitating treatment adherence by people with TB through nutritional and/or economic support

Participants discussed the need for social protection including insurance, transportation allowance, and nutritional support for TB-affected people (Figure 2).

The potential for economic support to improve nutrition, defray travel and other out-of-pocket costs, and increase TB treatment success, was raised in all FGDs. It was acknowledged, particularly in FGDs with NTP stakeholders and people with TB, that the government of Nepal provides Rs 3,000/month (~USD 27 for ambulatory MDR-TB cases and Rs 1,000/month (~USD 8) for those staying at DR-TB hostels. While the NTP stipulates that this is intended as nutritional and/or transport allowance, participants noted that how the cash is spent is not monitored. In addition, issues were raised with this existing transfer scheme, including delays in delivery of the allowance.

> **FGD with civil society organization, 45-50 years age group, male:** *"The most important question is when to give the allowance. It would be better in the first phase [of treatment] because it is [most] valuable at this time when one needs it most."*

There was further debate concerning whether cash or nutritional support was most appropriate. Some community stakeholders raised concerns regarding misuse of cash payments (e.g. to buy alcohol) and suggested that it would be preferable to provide nutritious food such as milk, ghee

For peer review only - http://bmjopen.bmj.com/site/about/guidelines.xhtml

(local butter), meat, and eggs. However, females with TB perceived that any cash received would nevertheless be spent on food, primarily staple foods such as rice, to feed their household.

Finally, it was perceived across FGDs that any nutritional or economic support should either be provided to all or stratified by need rather than TB drug resistance profile.

> **FGD with people diagnosed with TB, 30-35 years age group, male:** *"The government should provide nutritious food based on the economic status of patients. Drug-sensitive patients should also be provided with an allowance based on their level of poverty."*

III. Psychosocial

Theme: Psychosocial barriers to treatment adherence

Across all FGDs, stigma was perceived to be a significant barrier to seeking, accessing, and engaging with TB diagnostic and treatment services. People with TB described feeling discriminated against, mistreated, isolated, or hated. They reported perceptions or instances of people talking behind their back or remaining physically distanced. Participants of the FGD with community stakeholders shared that sometimes people with TB experienced extreme negative behavior such as physical or psychological mistreatment from their own family members. The situation was discussed as being even more pronounced for young married females because of a lack of personal agency within their husband's family. While this stigmatizing behaviour towards people with TB was reported to occur across socioeconomic groups, participants described that a *'blame and shame culture'* was prevalent among family members belonging to groups perceived as "higher" in the caste-related, social hierarchy. In alignment with this assertion, community mobilisers mentioned that lack of social and family support can cause people to conceal their TB status and not adhere to or complete TB treatment.

Reports of stigmatizing behaviour were not limited to the community and family members. Perceived negative behaviour of healthcare providers towards people with TB was noted across FGDs as an issue that compounded self-stigma and led to a breakdown of trust within the client-

For peer review only - http://bmjopen.bmj.com/site/about/guidelines.xhtml

provider relationship. Although participants felt that, generally, enacted stigma had decreased in Nepalese communities, FGDs with people with TB and community stakeholders shared that people still fear TB disease, especially in rural villages.

FGD with people diagnosed with TB, 30-35 years age group, male: *"People may know they have symptoms of TB but are too ashamed to go to the health facilities. People can't say out loud that they have TB. TB is regarded as a big disease and people get criticized for having it. The community perceives a TB patient differently than a normal person due to lack of awareness. That's why it's difficult to end TB."*

People with MDR-TB reported profound psychosocial impact including anxiety and isolation, especially during the first months of treatment. Depression, suicidal ideation, and shame related to stigma and also well-recognized side effects of MDR-TB medications were mentioned[23].

FGD with people diagnosed with MDR-TB, 45-50 years age group, male: *"I wanted to die. One of my friends [with MDR-TB] committed suicide after 16 months [of treatment]."*

Theme: Mutual or social support as a facilitator to treatment adherence

Social support from family and friends was perceived as a facilitator to adhering to TB treatment and becoming cured (Figure 2). This included visiting, spending time with, and showing affection towards people with TB to demonstrate solidarity and reduce feelings of isolation.

People with TB also shared the importance of mutual support beyond family and friends, including the wider community, leaders, elders, and other important local figures. The participants believed that this kind of support would help people with TB to cope and reflected the close communities and rich socio-cultural values inherent to Nepalese culture.

FGD with people diagnosed with MDR-TB, 40-45 years age group, male: *"My friends and the people in my village told me 'TB is a normal disease and encouraged me that, if I took my medicine, I'd be alright."*

For peer review only - http://bmjopen.bmj.com/site/about/guidelines.xhtml

Interactions and consultations with healthcare providers were also seen as opportune occasions to provide education and counseling to address the psychological impact of TB. FGD with community stakeholders raised the perceived importance of healthcare providers simply recognizing, acknowledging, and being understanding of patients' fears, concerns, and expectations. While healthcare provider-led counselling on medication adherence was noted to be commonplace at treatment initiation, counselling TB patients about TB-related fear, stigma, depression, and anxiety was broadly overlooked. It was noted that integration of medication and psychological counselling by healthcare providers could be a suitable method to deliver clear and open information about stigma and discrimination, which could improve TB treatment adherence and completion rates and potentially support mental wellness and empowerment.

Figure 2: Barriers and facilitators to accessing tuberculosis care in Nepal

(Authors suggest to use the same color for online prints as used in the figure)

DISCUSSION

This qualitative study generated new evidence regarding barriers and facilitators to accessing and engaging with TB services in Nepal. Multi-sectoral stakeholder participants highlighted that the barriers were predominantly related to the poor socioeconomic conditions of people with TB, including lack of education and endemic poverty. The findings showed that the costs of care-seeking and clinic-based DOTS can further compound poverty and, when combined with psychological impacts including stigma and anxiety, were perceived to negatively influence access to TB services. Participants cited multiple potential socioeconomic interventions, both integrated and discrete, including TB education, economic, nutritional, and social support, to mitigate catastrophic costs of TB-affected households and support people with TB to get cured.

Knowledge and awareness about TB

Low education levels and limited awareness of TB are associated with delays in healthcare seeking.[24–26] A study in Nepal showed inadequate knowledge of TB was associated with

For peer review only - http://bmjopen.bmj.com/site/about/guidelines.xhtml

increased likelihood of consulting traditional healers, resulting in TB diagnostic delay.[27] Our findings are also similar to other studies that suggested knowledge about TB was limited in poor, marginalized, and/or rural communities in Nepal.[8,28] This implies that any existing TB education and advocacy programs may not be reaching crucial, high-risk target groups and new approaches are required if Nepal is to end TB.

Educational support interventions that enhance knowledge about TB transmission, symptoms, treatment, and prevention, are important contributory factors in both care-seeking behaviour and treatment outcomes in diverse settings.[27,29,30] In India, the Global Fund-supported advocacy, communication, and social mobilization project, “Axshya”, has made progress towards reaching underserved groups through intense community outreach and education.[31] FGD participants in our study cited a dearth of awareness-raising interventions and campaigns in recent years in Nepal. Previously, similar campaigns focused on TB awareness through door-to-door visits, health promotion at health facilities, or educational outreach into communities. Such campaigns were perceived to increase knowledge on TB, advocate for free TB services, and empower communities to make informed choices. As highlighted by participants, in addition to commonly used platforms such as leaflets, radio, and television, future educational campaigns in Nepal would benefit from using technology such as mobile phones - which are used by over 90% households in both urban and rural areas[32] - or, where appropriate, social media.

Psychosocial impact

Of the perceived psychosocial barriers to accessing TB diagnosis and care in Nepal, stigma predominated. Participants mentioned feelings of guilt among people with TB, fear of disclosure, and experience of discrimination. This mirrors findings from diverse settings, which show that experiences of stigma are highly prevalent among people with TB and can impede access to TB services.[33] For example, in Zambia, a cohort study showed that anticipated and enacted stigma of people with TB resulted in delayed diagnosis, poor treatment adherence, reduced quality of life, and represented a distinct challenge to successful screening of their household contacts.[34]

For peer review only - http://bmjopen.bmj.com/site/about/guidelines.xhtml

It was notable that people with MDR-TB reported severe negative psychosocial impacts of their illness. These included profound feelings of anxiety, isolation related not only to their diagnosis but also to physical distance from their families, and recognized side effects of certain MDR-TB medications (e.g. Cycloserine) such as depression and despair.[35–37] Participants perceived an association between the psychosocial impact of MDR-TB and the potential for non-adherence to long, arduous treatment regimens including injectable agents. Discussion across FGDs suggested that existing medication adherence counselling delivered by NTP staff at treatment initiation would be a suitable platform upon which to integrate complementary psychosocial counselling about overcoming TB-related stigma and addressing ill mental health.

Economic burden of TB

The economic impact of accessing TB diagnosis and care was perceived to be severe. This was mainly due to high costs associated with transportation to clinics, maintaining adequate nutrition, and time and income loss. Participants with MDR-TB indicated that there was delay or unavailability of the NTP's financial assistance scheme during their treatment. The financial impact of belonging to a household affected by TB was cited in FGDs as forcing households to resort to coping strategies such as taking out loans, using savings, and selling assets. These findings are in line with the rapidly growing global body of evidence relating to the economic burden of TB. Such findings suggest that coping strategies remain common and only limited progress has been made towards the WHO target of "zero TB-affected families face catastrophic costs by 2020".[1,38] TB Patient Costs Surveys conducted in various LMICs have demonstrated that a substantial proportion of TB-affected households incur catastrophic costs, which can push them into further impoverishment and contribute to adverse TB treatment outcomes.[6,39–44] Studies demonstrated that more than 60% of TB-affected households in Nepal incurred catastrophic costs[6,7] and stark economic impact.[27,45–47]

Although this study focused on barriers amenable to interventions at the household level rather than health system level, our findings showed that when TB diagnosis and care were sought from

For peer review only

both the public and private sector, patient pathways to TB diagnosis and care were protracted and their costs, especially out-of-pocket costs, escalated (described in detail in Supplementary File II). This finding is consistent with the findings of systematic reviews from Nepal, India and Uganda,[48–54] which also highlighted that interventions to strengthen public-private partnerships can streamline diagnostic and referral pathways and potentially increase TB notifications to the NTP. Studies in India and Vietnam have demonstrated enhanced engagement with private pharmacies and medical practioners by providing them subsidies directly or through intermediary agencies for every notified case.[55,56] This could be a potential strategy to improve access to TB care in Nepal, where approximately 20% people with TB receive paid treatment from the private sector.[57]

Social and economic support

To address the psychosocial and economic impact of TB and improve TB cure and prevention rates, our findings imply a need for both social and economic support. This is supported by the results of studies in Nepal and other LMICs, which showed that providing both counseling and economic assistance to people with MDR-TB improved cure rates.[9,58–60] However, it must be noted that a significant proportion of people with DS-TB in Nepal experience enduring psychological, social and economic impacts of TB but receive no additional support.[6,7,12] In line with a study from Ethiopia,[61] our findings also suggest that the timing of provision of financial support is important. Participants advocated for early cash support delivery in the initiation phase of treatment when they perceived it to be needed most. Involvement of family members and peers in such interventions was also noted by participants as a vital aspect of support to complete TB treatment. This has also been reported in a systematic review of factors affecting medication adherence in LMICs.[62]

A well-designed socioeconomic support intervention would ideally be tailored or stratified to the individual or household needs of a person with TB and include overlapping elements such as increasing knowledge, awareness-raising, cost mitigation (e.g. through cash transfers or

For peer review only - http://bmjopen.bmj.com/site/about/guidelines.xhtml

transport vouchers) and stigma-reduction activities (e.g. mutual support, peer groups, enhanced medication counselling sessions), integrated into existing TB services.[63,64] However, it must be acknowledged that such an intervention would need to balance stratification with feasibility and pragmatism.

Strengths and limitations

Our study fills an important gap in knowledge about household-level socio-economic barriers to accessing TB services in Nepal and expanded on perceived facilitators and enablers to overcome these barriers. A major strength of the study methods was the trustworthiness and validity harnessed by garnering perspectives of the diverse study participants.[65]

The study has several limitations. As the participants were predominantly from Terai plains districts the findings, therefore, should be cautiously applied in other settings or countries. Nevertheless, we described the study setting to improve transferability. Second, participants from NGOs and healthcare professionals working with the NTP were over-represented within the participant cohort. However, we tried to strike a suitable balance by including the views of people with TB and community groups, which historically have been overlooked in similar research. FGDs consisted of limited women participants. We minimized the issue by specifically describing the female participants' viewpoint in the analysis. Similarly, there was no participation of people diagnosed with TB in private sectors or those who had been loss to follow-up who are the vulnerable to restricted access to TB services and poor outcomes. Their engagement would enable us to fully understand the most important barriers to care.[39,64]

CONCLUSION

There are multiple socioeconomic barriers to accessing and engaging with TB services in Nepal. TB education and advocacy, economic support, and psychosocial counselling integrated with medication adherence counselling could address these barriers and potentially reduce stigma, mitigate TB-related costs, and improve TB treatment outcomes. These elements are now being

For peer review only - http://bmjopen.bmj.com/site/about/guidelines.xhtml

incorporated into the design of a locally-appropriate socioeconomic support intervention for TB-affected households for pilot implementation in Nepal.

For peer review only

For peer review only - http://bmjopen.bmj.com/site/about/guidelines.xhtml

Abbreviations

BNMT Birat Nepal Medical Trust

DOTS Directly Observed Treatment Shortcourse

DS-TB Drug Suceptible TB

MDR-TB Multi Drug Resistant-TB

NTP National Tuberculosis Program

SDG Sustainable Development Goal

TB Tuberculosis

Acknowledgment

We would like to acknowledge National Tuberculosis Control Center, Ministry of Health and Population, Nepal Health Research Council, Health Directorate of Province 2 and Province 3, and all related health facilities and authorities for their support and collaboration with this project. Most importantly we are grateful to people with TB for their generosity of time and sharing their feedback and experiences during this study.

Contributorship statement:

Kritika Dixit (KD): Data Curation, Investigation, Project Administration, Methodology, Analysis, First draft preparation, Writing- Review & Editing

Olivia Biermann (OB), Kristi Sidney Annerstedt (KSA): Methodology, Writing – Review & Editing

Bhola Rai (BR), Puskar Raj Paudel (PRP): Data Curation, Project Administration, Writing – Review & Editing

For peer review only - http://bmjopen.bmj.com/site/about/guidelines.xhtml

Tara Prasad Aryal (TPA), Ram Narayan Pandit (RNP), Manoj Kumar Sah (MKS), Govinda Majhi (GoM): Investigation, Project Administration, Writing – Review & Editing

Gokul Mishra (GM): Investigation, Methodology, Supervision, Writing – Review & Editing

Noemia Teixeira de Siqueira-Filha (NTSF): Data Curation, Formal Analysis, Investigation, Methodology, Project Administration, Writing – Original Draft Preparation, Writing – Review & Editing

Jens W. Levy (JWL), Job van Rest (JvR): Data Curation, Resources, Validation, Writing – Review & Editing

Suman Chandra Gurung (SCG), Raghu Dhital (RG): Investigation, Project Administration, Resources, Supervision, Writing – Original Draft Preparation, Writing – Review & Editing

Knut Lönnroth (KL), S Bertel Squire (SBS): Conceptualization, Methodology, Supervision, Writing – Original Draft Preparation, Writing – Review & Editing

Maxine Caws (MC): Conceptualization, Formal Analysis, Funding Acquisition, Investigation, Methodology, Project Administration, Supervision, Writing – Original Draft Preparation, Writing – Review & Editing

Tom Wingfield (TW): Conceptualization, Data Curation, Formal Analysis, Funding Acquisition, Investigation, Methodology, Project Administration, Supervision, Validation, Visualization, Writing – Original Draft Preparation, Writing – Review & Editing

Competing interest: Authors declare no competing interest

Funding

KD receives support from the Farrar Foundation and Royal Society of Tropical Medicine and Hygiene (RSTMH) and National Institute of Health Research (NIHR). TW is supported by grants from: the Wellcome Trust, UK (209075/Z/17/Z); the Medical Research Council, Department for International Development, and Wellcome Trust (Joint Global Health Trials, MR/V004832/1), the Academy of Medical Sciences, UK; and the Swedish Health Research Council, Sweden. All other

For peer review only - http://bmjopen.bmj.com/site/about/guidelines.xhtml

authors (KD, OB, BR, TPA, GM, GoM, NTSF, PRP, RNP, MKS, JL, JvR, SCG, RD, KL, SBS, MC, KSA) are supported by EU Horizon2020 grant 733174 IMPACT TB.

Data availability statement: All data relevant to the study are included in the article or uploaded as supplementary information

For peer review only

For peer review only - http://bmjopen.bmj.com/site/about/guidelines.xhtml

REFERENCES

1. World Health Organization (WHO). Global Tuberculosis Report [Internet]. Geneva; 2020 [cited 2020 Oct 20]. Available from: https://apps.who.int/iris/bitstream/handle/10665/336069/9789240013131-eng.pdf?ua=1

2. Wingfield T, Tovar MA, Datta S, et al. Addressing social determinants to end tuberculosis. Lancet. 2018;391(10126):1129–32.

3. Wingfield T, Tovar MA, Huff D, et al. Beyond pills and tests: addressing the social determinants of tuberculosis. Clinical medicine. 2016;16:s79–91.

4. World Health Organization. Implementing the End TB Strategy: The essentials [Internet]. Geneva, Switzerland: World Health Organization; 2015 [cited 2020 Sep 8]. Available from: https://www.who.int/tb/publications/2015/end_tb_essential.pdf?ua=1

5. National Tuberculosis Control Center. Nepal National TB Prevalence Survey Brief [Internet]. 2020 [cited 2020 Apr 19]. Available from: https://nepalntp.gov.np/wp-content/uploads/2020/03/NEPAL-NATIONAL-TB-PREVALENCE-SURVEY-BRIEF-March-24-2020.pdf

6. Gurung S, Dixit K, Rai B, et al. The role of active case finding in reducing patient incurred catastrophic costs for tuberculosis in Nepal. Infectious Diseases of Poverty. 2019;8(99).

7. Gurung SC, Rai B, Dixit K, et al. Active case finding reduces household costs for people affected by tuberculosis : The IMPACT TB longitudinal costing survey in Nepal. Health Policy and Planning. 2020;

8. Bam TS, Gunneberg C, Chamroonsawasdi K, et al. Factors affecting patient adherence to DOTS in urban Kathmandu , Nepal. The International Journal of Tuberculosis and Lung Disease [Internet]. 2006 [cited 2020 Jan 28];10(9517):270–6. Available from: http://docserver.ingentaconnect.com/deliver/connect/iuatld/10273719/v10n3/s9.pdf?expires=1605638319&id=0000&titleid=3764&checksum=74FA4BE1638619D0003281BF3196

For peer review only - http://bmjopen.bmj.com/site/about/guidelines.xhtml

64C4

9. Baral SC, Aryal Y, Bhattrai R, et al. The importance of providing counselling and financial support to patients receiving treatment for multi-drug resistant TB: mixed method qualitative and pilot intervention studies. BMC Public Health [Internet]. 2014 Dec 17 [cited 2020 Jan 28];14(1):46. Available from: http://bmcpublichealth.biomedcentral.com/articles/10.1186/1471-2458-14-46

10. de Cuevas RMA, Lawson L, Al-Sonboli N, et al. Patients direct costs to undergo TB diagnosis. Infectious Diseases of Poverty [Internet]. 2016 [cited 2020 Jan 28];5. Available from: https://idpjournal.biomedcentral.com/articles/10.1186/s40249-016-0117-x

11. NTC. National Tuberculosis Program Nepal. Annual report 2074/75 (2018). Vol. 75. 2018.

12. Rai B, Dixit K, Aryal TP, et al. Developing feasible, locally appropriate socioeconomic support for TB-affected households in Nepal. Tropical Medicine and Infectious Disease. 2020;5(2).

13. Vinicius K, Andrade F De, Nery JS, et al. Effects of social protection on tuberculosis treatment outcomes in low or middle-income and in high-burden countries : systematic review and meta-analysis Efeitos da proteção social sobre os desfechos do tratamento da tuberculose em países de renda baixa . 2018;34(1):1–18.

14. Gomes J, Oliosi N, Reis-santos B, et al. Effect of the Bolsa Familia Programme on the outcome of tuberculosis treatment : a prospective cohort study. Lancet Glob Health [Internet]. 2018 [cited 2020 Jan 28];219–26. Available from: https://www.thelancet.com/journals/langlo/article/PIIS2214-109X(18)30478-9/fulltext

15. Richterman A, Steer-massaro J, Jarolimova J, et al. Cash interventions to improve clinical outcomes for pulmonary tuberculosis : systematic review and meta-analysis. Bull World Health Organ [Internet]. 2018 [cited 2020 Jan 28];(January):471–83. Available

For peer review only - http://bmjopen.bmj.com/site/about/guidelines.xhtml

from: https://www.ncbi.nlm.nih.gov/pmc/articles/PMC6022611/

16. Hoorn R Van, Jaramillo E, Collins D, et al. The Effects of Psycho-Emotional and Socio-Economic Support for Tuberculosis Patients on Treatment Adherence and Treatment Outcomes – A Systematic Review and Meta- Analysis. Plos One. 2016;1–27.

17. Dixit K, Rai B, Prasad Aryal T, et al. Research protocol for a mixed-methods study to characterise and address the socioeconomic impact of accessing TB diagnosis and care in Nepal. Wellcome Open Research. 2020;5(19).

18. Tong A, Sainsbury P, Craig J. Consolidated criteria for reporting qualitative research (COREQ): A 32-item checklist for interviews and focus groups. International Journal for Quality in Health Care [Internet]. 2007 [cited 2020 Jul 22];19(6):349–57. Available from: https://doi.org/10.1093/intqhc/mzm042

19. Malterud K, Siersma VD, Guassora AD. Sample Size in Qualitative Interview Studies : Guided by Information Power. Qualitative Heath Research [Internet]. 2016 [cited 2020 May 11];26(13):1753–60. Available from: https://pubmed.ncbi.nlm.nih.gov/26613970/

20. Braun V, Clarke V. Using thematic analysis in psychology. Qualitative Research in Psychology [Internet]. 2006 [cited 2020 Sep 11];3(2):77–101. Available from: https://www.tandfonline.com/doi/abs/10.1191/1478088706qp063oa

21. Holloway I, Wheeler S. Qualitative Research in Nursing and Healthcare [Internet]. 3rd Editio. John Wiley & Sons. Wiley-Blackwell; 2010. 1–351 p. Available from: http://eu.wiley.com/WileyCDA/WileyTitle/productCd-1405161221.html

22. Weaver MS, Lönnroth K, Howard SC, et al. Interventions to improve adherence to treatment for paediatric tuberculosis in low- and middle-income countries: a systematic review and meta-analysis. Bulletin of the World Health Organization [Internet]. 2015 [cited 2020 Jul 29];93(10):700-711B. Available from: https://www.ncbi.nlm.nih.gov/pmc/articles/PMC4645428/

For peer review only - http://bmjopen.bmj.com/site/about/guidelines.xhtml

23. Vega P, Sweetland A, Acha J, et al. Psychiatric issues in the management of patients with multidrug-resistant tuberculosis. International Journal of Tuberculosis and Lung Disease [Internet]. 2004 [cited 2020 Jul 22];8(November 2003):749–59. Available from: http://docserver.ingentaconnect.com/deliver/connect/iuatld/10273719/v8n6/s11.pdf?expires=1605965794&id=0000&titleid=3764&checksum=BB7C2606D65A6484F704EDA415435129

24. Huq KATME, Moriyama M, Zaman K, et al. Health seeking behaviour and delayed management of tuberculosis patients in rural Bangladesh. BMC Infectious Diseases [Internet]. 2018 [cited 2020 Apr 19];18(515). Available from: https://bmcinfectdis.biomedcentral.com/articles/10.1186/s12879-018-3430-0

25. Tola HH, Tol A, Shojaeizadeh D. Tuberculosis Treatment Non-Adherence and Lost to Follow Up among TB Patients with or without HIV in Developing Countries : A Systematic Review. Bull World Health Organ [Internet]. 2015 [cited 2020 Jan 28];44(1). Available from: https://www.ncbi.nlm.nih.gov/pmc/articles/PMC4449995/

26. Gelaw SM. Socioeconomic Factors Associated with Knowledge on Tuberculosis among Adults in Ethiopia. Tuberculosis Research and Treatment [Internet]. 2016 [cited 2020 Sep 12];2016. Available from: http://downloads.hindawi.com/journals/trt/2016/6207457.pdf

27. Marahatta SB, Yadav RK, Giri D, et al. Barriers in the access, diagnosis and treatment completion for tuberculosis patients in central and western Nepal: A qualitative study among patients, community members and health care workers. PloS one [Internet]. 2020 [cited 2020 Sep 13];15(1):e0227293. Available from: https://journals.plos.org/plosone/article?id=10.1371/journal.pone.0227293

28. Khanal S, Elsey H, King R, et al. Development of a patient-centred, psychosocial support intervention for multi-drug-resistant tuberculosis (MDR-TB) care in Nepal. PLoS ONE [Internet]. 2017 [cited 2020 Apr 19];12(1):1–16. Available from:

For peer review only - http://bmjopen.bmj.com/site/about/guidelines.xhtml

https://journals.plos.org/plosone/article?id=10.1371/journal.pone.0167559

29. Westerlund EE, Tovar MA, Lönnermark E, et al. Tuberculosis-related knowledge is associated with patient outcomes in shantytown residents; Results from a cohort study, Peru. Journal of Infection [Internet]. 2015 [cited 2020 Jul 22];71(3):347–57. Available from: https://www.journalofinfection.com/article/S0163-4453(15)00188-7/fulltext

30. Mahato RK, Laohasiriwong W, Vaeteewootacharn K. Major Delays in the Diagnosis and Management of Tuberculosis Patients in Nepal. Journal of Clinical and Diagnostic Research [Internet]. 2015 [cited 2020 Feb 1];9(10):5–9. Available from: https://www.ncbi.nlm.nih.gov/pmc/articles/PMC4625264/#:~:text=reduce such delays.-,Conclusion,facilities%2C poor knowledge on TB.

31. Thapa B, Chadha SS, Das A, et al. High and equitable tuberculosis awareness coverage in the community-driven Axshya TB control project in India. Public Health Action [Internet]. 2015 [cited 2020 Jul 22];5(1):70–3. Available from: https://europepmc.org/article/pmc/4525368

32. Ministry of Health, New ERA, ICF. Nepal Demographic and Health Survey 2016 [Internet]. Kathmandu; 2017 [cited 2020 Nov 17]. Available from: https://www.dhsprogram.com/pubs/pdf/fr336/fr336.pdf

33. Ngamvithayapong-Yanai J, Luangjina S, Thawthong S, et al. Stigma against tuberculosis may hinder non-household contact investigation: a qualitative study in Thailand. Public Health Action [Internet]. 2019 [cited 2020 Nov 1];9(1):15–23. Available from: https://www.ncbi.nlm.nih.gov/pmc/articles/PMC6436487/pdf/i2220-8372-9-1-15.pdf

34. Cremers AL, Laat MM De, Kapata N, et al. Assessing the Consequences of Stigma for Tuberculosis Patients in Urban Zambia. Plos One [Internet]. 2015 [cited 2020 Apr 19];10(3). Available from: https://pubmed.ncbi.nlm.nih.gov/25806955/

35. Munro SA, Lewin SA, Smith HJ, et al. Patient Adherence to Tuberculosis Treatment : A

For peer review only - http://bmjopen.bmj.com/site/about/guidelines.xhtml

Systematic Review of Qualitative Research. Plos Medicine [Internet]. 2007 [cited 2020 Aug 6];4(7). Available from: https://journals.plos.org/plosmedicine/article?id=10.1371/journal.pmed.0040238

36. Stosic M, Vukovic D, Babic D, et al. Risk factors for multidrug-resistant tuberculosis among tuberculosis patients in Serbia : a case-control study. BMC Public Health [Internet]. 2018 [cited 2020 Nov 18];18(1114). Available from: https://bmcpublichealth.biomedcentral.com/articles/10.1186/s12889-018-6021-5

37. Marahatta S, Kaewkungwal J, Ramasoota P, et al. Risk factors of Multidrug Resistant Tuberculosis in central Nepal : A pilot study. Kathmandu University Medical Journal [Internet]. 2010 [cited 2020 Oct 20];9(32):392–7. Available from: https://www.nepjol.info/index.php/KUMJ/article/view/6238

38. Uplekar M, Weil D, Lonnroth K, et al. WHO's new end TB strategy. The Lancet [Internet]. 2015 [cited 2020 Jul 2];385(9979):1799–801. Available from: https://linkinghub.elsevier.com/retrieve/pii/S0140-6736(15)60570-0

39. Wingfield T, Boccia D, Tovar M, et al. Defining Catastrophic Costs and Comparing Their Importance for Adverse Tuberculosis Outcome with Multi-Drug Resistance: A Prospective Cohort Study, Peru. PLoS Medicine. 2014;11(7).

40. Tanimura T, Jaramillo E, Weil D, et al. Financial burden for tuberculosis patients in low-And middle-income countries: A systematic review. European Respiratory Journal [Internet]. 2014 [cited 2020 Jul 2];43(6):1763–75. Available from: https://erj.ersjournals.com/content/erj/43/6/1763.full.pdf

41. Hoa N, Nhung N. National tuberculosis patients cost survey: research findings lead to change in policy and practice. Viet Nam. Public Health Action [Internet]. 2019 [cited 2020 Jul 22];9(2):50–2. Available from: https://www.ncbi.nlm.nih.gov/pmc/articles/PMC6645445/

For peer review only

For peer review only - http://bmjopen.bmj.com/site/about/guidelines.xhtml

42. Viney K, Islam T, Hoa NB, et al. The Financial Burden of Tuberculosis for Patients in the Western-Pacific Region. Tropical Medicine and Infectious Disease [Internet]. 2019 [cited 2020 Jul 22];4(2):94. Available from: https://www.ncbi.nlm.nih.gov/pmc/articles/PMC6631110/

43. Pedrazzoli D, Borghi J, Viney K, et al. Measuring the economic burden for TB patients in the end TB strategy and universal health coverage frameworks. International Journal of Tuberculosis and Lung Disease. 2019;23(1):5–11.

44. Mcintyre D, Thiede M, Whitehead M. What are the economic consequences for households of illness and of paying for health care in low- and middle-income country contexts ? Social Science & Medicine [Internet]. 2006 [cited 2020 Apr 19];62:858–65. Available from: https://www.sciencedirect.com/science/article/abs/pii/S0277953605003631?via%3Dihub

45. Mishra P, Hansen EH, Sabroe S, et al. Socio-economic status and adherence to tuberculosis treatment: a case-control study in a district of Nepal. Int J Tuberc Lung Dis [Internet]. 2005 [cited 2020 Jan 28];9(10):1134–9. Available from: http://docserver.ingentaconnect.com/deliver/connect/iuatld/10273719/v9n10/s11.pdf?expires=1605975237&id=0000&titleid=3764&checksum=7F8B03920562B289941E5A89AAD1F81A

46. Laohasiriwong W, Mahato RK, Koju R, et al. Delay for First Consultation and Its Associated Factors among New Pulmonary Tuberculosis Patients of Central Nepal. Tuberculosis Research and Treatment [Internet]. 2016;2016:4583871. Available from: http://downloads.hindawi.com/journals/trt/2016/4583871.pdf

47. Zegeye A, Dessie G, Wagnew F, et al. Prevalence and determinants of anti-tuberculosis treatment non-adherence in Ethiopia: A systematic review and meta-analysis. PloS one [Internet]. 2019 [cited 2020 Sep 13];14(1):e0210422. Available from: https://journals.plos.org/plosone/article/file?id=10.1371/journal.pone.0210422&type=print

For peer review only - http://bmjopen.bmj.com/site/about/guidelines.xhtml

able

48. Ha A, Bijlsma MW, Malla P, et al. The road to tuberculosis treatment in rural Nepal : A qualitative assessment of 26 journeys. BMC Health Services Research [Internet]. 2008 [cited 2020 Jan 28];10:1–10. Available from: https://bmchealthservres.biomedcentral.com/articles/10.1186/1472-6963-8-7

49. Arinaminpathy N, Batra D, Khaparde S, et al. The number of privately treated tuberculosis cases in India: an estimation from drug sales data. The Lancet Infectious Diseases [Internet]. 2016 [cited 2020 Jul 22];16(11):1255–60. Available from: https://www.thelancet.com/action/showPdf?pii=S1473-3099%2816%2930259-6

50. Joloba M, Mwangi C, Alexander H, et al. Strengthening the Tuberculosis Specimen Referral Network in Uganda: The Role of Public-Private Partnerships. Journal of Infectious Diseases [Internet]. 2016 [cited 2020 Nov 1];213(S2):S41–6. Available from: https://www.ncbi.nlm.nih.gov/pmc/articles/PMC4914746/

51. Subbaraman R, Nathavitharana RR, Satyanarayana S, et al. The Tuberculosis Cascade of Care in India's Public Sector: A Systematic Review and Meta-analysis. PLoS Medicine [Internet]. 2016 [cited 2020 Jul 22];13(10):1–38. Available from: https://journals.plos.org/plosmedicine/article/file?id=10.1371/journal.pmed.1002149&type=printable

52. Storla DG, Yimer S, Bjune GA. A systematic review of delay in the diagnosis and treatment of tuberculosis. BMC Public Health [Internet]. 2008 [cited 2020 Jul 22];8:1–9. Available from: https://bmcpublichealth.biomedcentral.com/articles/10.1186/1471-2458-8-15

53. Buregyeya E, Criel B, Nuwaha F, et al. Delays in diagnosis and treatment of pulmonary tuberculosis in Wakiso and Mukono districts, Uganda. BMC public health [Internet]. 2014 [cited 2020 Jul 22];14:586. Available from:

For peer review only

For peer review only - http://bmjopen.bmj.com/site/about/guidelines.xhtml

https://bmcpublichealth.biomedcentral.com/articles/10.1186/1471-2458-14-586

54. Kamineni VV, Wilson N, Das A, et al. Addressing poverty through disease control programmes: Examples from Tuberculosis control in India. International Journal for Equity in Health [Internet]. 2012 [cited 2020 Jul 22];11(1). Available from: https://equityhealthj.biomedcentral.com/articles/10.1186/1475-9276-11-17

55. Anand T, Babu R, Jacob A, et al. Enhancing the role of private practitioners in tuberculosis prevention and care activities in India. Lung India [Internet]. 2017;34(6):538. Available from: http://www.lungindia.com/text.asp?2017/34/6/538/217577

56. Vo LNQ, Codlin AJ, Huynh HB, et al. Enhanced Private Sector Engagement for Tuberculosis Diagnosis and Reporting through an Intermediary Agency in Ho Chi Minh City, Viet Nam. Tropical Medicine and Infectious Disease [Internet]. 2020 Sep 14;5(3):143. Available from: https://www.mdpi.com/2414-6366/5/3/143

57. National Tuberculosis Control Center. Annual Report 2075/76 (2018/19) [Internet]. 2019 [cited 2020 Aug 6]. Available from: https://nepalntp.gov.np/wp-content/uploads/2020/04/NTP-Annual-Report-2075-76-2018-19.pdf

58. Carter DJ, Glaziou P, Lönnroth K, et al. The impact of social protection and poverty elimination on global tuberculosis incidence: a statistical modelling analysis of Sustainable Development Goal 1. The Lancet Global Health. 2018;6(5):e514–22.

59. Oliosi JGN, Reis-Santos B, Locatelli RL, et al. Effect of the Bolsa Familia Programme on the outcome of tuberculosis treatment: a prospective cohort study. The Lancet Global Health. 2018;219–26.

60. Klein K, Bernachea MP, Id SI, et al. Evaluation of a social protection policy on tuberculosis treatment outcomes : A prospective cohort study. PLoS Medicine. 2019;9:1–16.

For peer review only

61. Gebremariam MK, Bjune GA, Frich JC. Barriers and facilitators of adherence to TB treatment in patients on concomitant TB and HIV treatment: A qualitative study. BMC Public Health. 2010;10.

62. Vries SG de, Cremers AL, Heuvelings CC, et al. Barriers and facilitators to the uptake of tuberculosis diagnostic and treatment services by hard-to-reach populations in countries of low and medium tuberculosis incidence: a systematic review of qualitative literature. Lancet Infectious Disease [Internet]. 2017 [cited 2020 Jan 28];17(5):e128–43. Available from: https://bmcpublichealth.biomedcentral.com/articles/10.1186/1471-2458-10-651

63. Wingfield T, Boccia D, Tovar MA, et al. Designing and implementing a socioeconomic intervention to enhance TB control : operational evidence from the CRESIPT project in Peru. 2015;15.

64. Wingfield T, Tovar MA, Huff D, et al. The economic effects of supporting tuberculosis-affected households in Peru. Eur Respir J. 2016;48:1396–410.

65. Holloway Immy, Galvin Kathleen. Qualitative research in nursing and healthcare. John Wiley & Sons; 2016.

For peer review only

Reference (From table 1)

Table 1

[22] Weaver MS, Lönnroth K, Howard SC, et al. Interventions to improve adherence to treatment for paediatric tuberculosis in low- and middle-income countries: a systematic review and meta-analysis. Bull World Health Organ [Internet]. 2015 [cited 2020 Jul 29];93(10):700-711B. Available from: https://www.ncbi.nlm.nih.gov/pmc/articles/PMC4645428/

LEGEND

Figure 1: The highlighted color represents the study districts in Nepal. Dhanusha, Mahottari and Chitwan are 'plains' or 'Terai' districts. Makwanpur is a hilly district. The district's data for population numbers and TB case notification rate highlights the burden of tuberculosis in each district (National TB Control Center Annual Report, 2018).

Figure 2: The inner white circle contains the key categories that influence TB service access and engagement, which are adapted from a World Health Organization Medication Adherence Framework (see Methods).[22] The middle red circle indicates the main barriers identified for each category, which may threaten access to TB services. The outer green circle indicates the main facilitators (current or potential) for each category, which may enhance access to TB services. Barriers relating to "TB, health, and basic education", "Social protection and nutrition", and "Psychosocial" were perceived by the project team to be modifiable by a household level socioeconomic intervention. Barriers relating to the "Health System" were perceived by the project team to be non-modifiable by a household level socioeconomic intervention and are, therefore, separated from the other categories and represented by dotted lines. "PPM" as a health system barrier refers to the protracted and convoluted patient journey through public and private healthcare providers, which was reported as being associated with increased economic impact, especially related to out-of-pocket costs. The surrounding bidirectional arrows indicate the cross-FGD finding that adequate funding and advocacy, and political will and commitment were perceived as vital structural factors to enable the facilitators identified to overcome the barriers identified. **Abbreviations** TB: Tuberculosis; NTP: National Tuberculosis Program; DS-TB: Drug-sensitive Tuberculosis; DOTS: Directly Observed Treatment Shortcourse; PPM: Public-Private Mix. **Reference:** [22] Weaver MS, Lönnroth K, Howard SC, et al. Interventions to improve adherence to treatment for paediatric tuberculosis in low- and middle-income countries: a

For peer review only - http://bmjopen.bmj.com/site/about/guidelines.xhtml

systematic review and meta-analysis. Bull World Health Organ [Internet]. 2015 [cited 2020 Jul 29];93(10):700-711B. Available from: https://www.ncbi.nlm.nih.gov/pmc/articles/PMC4645428/

For peer review only

For peer review only - http://bmjopen.bmj.com/site/about/guidelines.xhtml

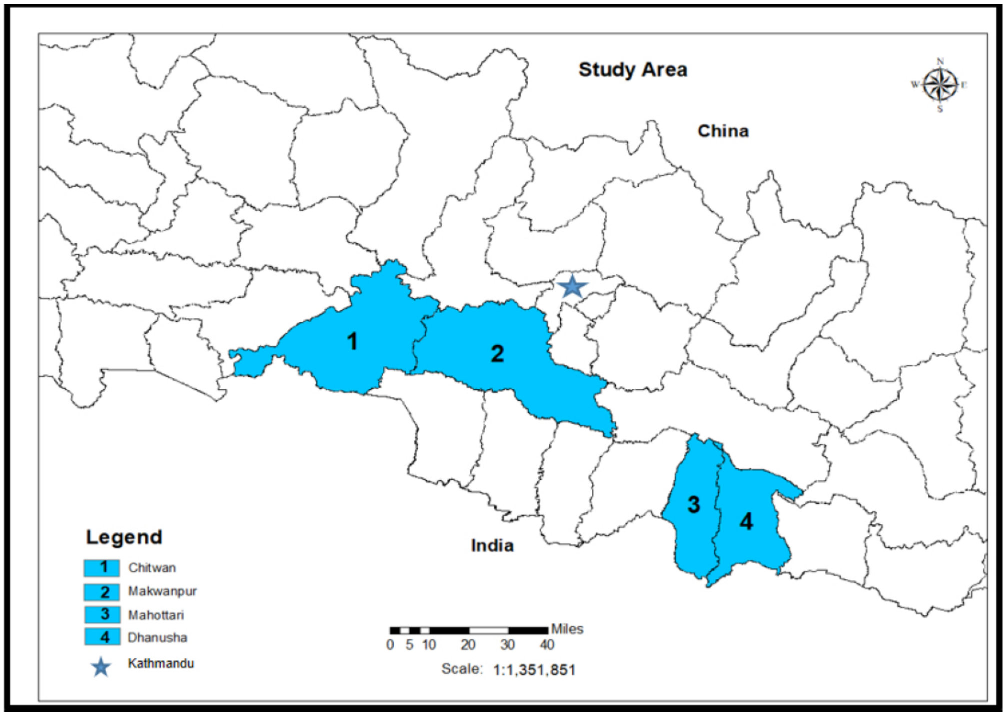

District	Population	Case Notification Rate (per 100,000 population)
Makwanpur	480,000	147
Chitwan	585,000	158
Dhanusha	823,000	100
Mahottari	681,000	131

Figure 1: The highlighted color represents the study districts in Nepal. Dhanusha, Mahottari and Chitwan are 'plains' or 'Terai' districts. Makwanpur is a hilly district. The district's data for population numbers and TB case notification rate highlights the burden of tuberculosis in each district (National TB Control Center Annual Report, 2018).

748x819mm (37 x 37 DPI)

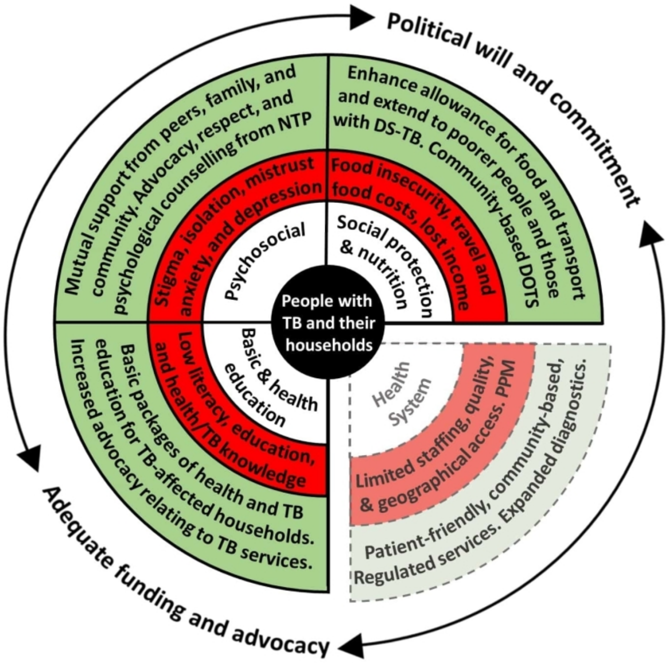

Figure 2: The inner white circle contains the key categories that influence TB service access and engagement, which are adapted from a World Health Organization Medication Adherence Framework (see Methods).[22] The middle red circle indicates the main barriers identified for each category, which may threaten access to TB services. The outer green circle indicates the main facilitators (current or potential) for each category, which may enhance access to TB services. Barriers relating to "TB, health, and basic education", "Social protection and nutrition", and "Psychosocial" were perceived by the project team to be modifiable by a household level socioeconomic intervention. Barriers relating to the "Health System" were perceived by the project team to be non-modifiable by a household level socioeconomic intervention and are, therefore, separated from the other categories and represented by dotted lines. "PPM" as a health system barrier refers to the protracted and convoluted patient journey through public and private healthcare providers, which was reported as being associated with increased economic impact, especially related to out-of-pocket costs. The surrounding bidirectional arrows indicate the cross-FGD finding that adequate funding and advocacy, and political will and commitment were perceived as vital structural factors to enable the facilitators identified to overcome the barriers identified. Abbreviations TB: Tuberculosis; NTP: National Tuberculosis Program; DS-TB: Drug-sensitive Tuberculosis; DOTS: Directly Observed Treatment Shortcourse; PPM: Public-Private Mix. Reference: 22 Weaver MS, Lönnroth K, Howard SC, et al. Interventions to improve adherence to treatment for paediatric tuberculosis in low- and middle-income countries: a systematic review and meta-analysis. Bull World Health Organ [Internet]. 2015 [cited 2020 Jul 29];93(10):700-711B. Available from: https://www.ncbi.nlm.nih.gov/pmc/articles/PMC4645428

537x506mm (96 x 96 DPI)

Supplementary File I: Focus group discussion coding framework

Category 1: TB, health, and basic education

**TB, health, and basic education barriers**				
**FGD**	**First order category**	**Second order themes**	**Third order themes**	**Example Quote**
People diagnosed with TB	TB, health, and basic education	Inadequate knowledge on TB services	Information barrier to access	**FGD with people diagnosed with TB, 20-25 years age group, female:** *"I didn't know that medicines were free in hospitals. I knew it only when I visited health post."*
People diagnosed with MDR-TB	TB, health, and basic education	Limited knowledge on TB disease	Information barrier to access	**FGD with people diagnosed with MDR-TB, 70-75 years age group, male:** *"No one told me anything [about TB] but I knew that it was a communicable disease."*
Community leaders	TB, health, and basic education	Inadequate advocacy on TB services	Information barrier to access	**FGD with community leaders, 45-50 years age group, male:** *"There is no person to advocate about [available health and TB] services."*
People diagnosed with MDR-TB	TB, health, and basic education	Limited knowledge on TB disease and TB medicines dosages	Information barrier to adhere to TB services	**FGD with people diagnosed with MDR-TB, 45-50 years age group,male:** *"I took medicine for 2 months and then left for 15 days because I thought I was feeling better."*
Civil society organization	TB, health, and basic education	Lack of awareness raising activities particularly in poorer and	Information barrier to access	**FGD with civil society organization, 45-50 years age group, male:** "*In addition, programmatic TB awareness-raising activities were seen as scarce, especially among poorer, "educationally and socially marginalized."*

For peer review only - http://bmjopen.bmj.com/site/about/guidelines.xhtml

		socially marginalized		
Civil society organization	TB, health, and basic education	Inadequate awareness raising educational materials	Information barrier to access	**FGD with civil society organization, 45-50 years age group, male: "***There has been few educational materials development for TB awareness at present in comparison to the past. Therefore, people do not go to the health facility."*
**TB, health, and basic education facilitators**				
**FGD**	TB, health, and basic education	**Second order theme**	**Third order theme**	**Quotes**
Civil society organization	TB, health, and basic education	Improvements in basic education on TB	Education on TB disease and advocacy about its services	**FGD with civil society organization, 45-50 years age group, male***: "TB education should be spread intensively to village households."*
People diagnosed with TB	TB, health, and basic education	Awareness about TB disease, treatment and prevention	Education on TB disease and advocacy about its services	**FGD with people diagnosed with TB, 30-35 years age group, male:** *The government should develop TB awareness raising programs….[including] in the media, to tell people about TB symptoms, check-ups….. and that it's a curable disease."*

For peer review

People diagnosed with TB	TB, health, and basic education	Community engagement<br><br>Mobilization of TB champions and peers	Education on TB disease and advocacy about its services	**FGD with people diagnosed with TB, 30-35 years age group, male:** *"People previously affected by TB could tell their story to community and mother's groups. I am 100% sure this will work."*
People diagnosed with TB	TB, health, and basic education	Awareness about TB<br><br>Information on TB medications	Education on TB disease and advocacy about its services	**FGD with people diagnosed with TB, 45-50 years age group, male:** *"People in the villages will take medicines regularly if they are educated….the government should strengthen education on TB medication."*
Civil society organization	TB, health, and basic education	Community engagement<br><br>Mobilization of community leaders for TB awareness	Education on TB disease and advocacy about its services	**FGD with civil society organization, 50-55 years age group, female:** *"We need to create [educational] groups attached to health centres and schools….Community and locally-elected leaders and teachers could give education to their communities and conduct TB awareness training and workshops."*
TB healthcare professional	TB, health, and basic education	Awareness about TB<br><br>Communication to influence health beliefs and change behaviour	Education on TB disease and advocacy about TB services	**FGD with TB healthcare professional, 55-60 years age group, male:** *"Changing [people's] attitude [about TB] is one of the things we can start doing to….. make a change in [people's] behaviour."*

Community Mobilizers	TB, health, and basic education	Awareness about TB<br><br>Improved communication to influence health beliefs and change behaviour	Education on TB disease and advocacy about TB services	**FGD with Community Mobilizers, 30-35 years age group, male:** *"[Some] patients don't believe us when we tell them that they have been diagnosed with TB. They say they'll go to India for check-up. In our region, people who believe in a certain religion think that TB will cure itself."*
People diagnosed with TB	TB, health, and basic education	Advocacy on TB services	Expansion of TB services in community	**FGD with people diagnosed with TB, 30-35 years age group, male:** ***"****Health facilities should conduct door to door campaign. [Health facility] should collect sputum in a timely manner. That's it, actually there is a lack of advocacy****."***
Community leaders	TB, health, and basic education	Prioritize underserved people	Education on TB disease and advocacy about TB services in remote communities	**FGD with community leaders, 65-70 years age group, male:** ***"****One of the reasons is that many people do not have adequate information because in this era radio, television, mic, seminars, workshops are organized where people/ community are literate. But those people who do not have access to any of these media are disadvantaged from these programs. So, we should focus more to those people as well."*

Category 2: Social protection and nutrition

**Social protection, nutrition, and economic barriers**

For peer review only

FGD	First order categories	Second order themes	Third order themes	Quote
People diagnosed with TB	Social protection, nutrition, and economic	Out-of-pocket expenses for transportation to engage with treatment	Economic barrier to treatment adherence	**FGD with people diagnosed with TB, 55-60 years age group, male**: *"When I don't have money, I cannot ride auto [local vehicle] because it costs Rs 300 [$2.5 USD] to reach home or clinic. I can't earn three pennies! How can I pay Rs 300 to go to home?"*
People diagnosed with TB	Social Protection, nutrition and economic	Out-of-pocket expenses for diagnostic tests	Economic barrier to access	**FGD with people diagnosed with TB, 45-50 years age group, male:** *"I visited all the pharmacies in my city, about 15-17 medicals [pharmacies] overall. I used to buy pneumonia medicine and take it every time. TB was not initially identified. After a month without taking any medicines, TB was identified [at the government clinic]….. The barrier is more financial. I spent approximately 1.7 lakhs [~1530 USD] for my treatment."*
Civil society organization	Social Protection, nutrition and economic	Out-of-pocket expenses for transportation and nutrition	Economic barrier to treatment adherence	**FGD with civil society organization, 50-55 years age group, female:** *"TB medicines are free but people also need costs for two-way transportation, food. TB illness [and even] TB treatment can make people weak and nutrition is needed. How can people afford [nutrition and transport costs]?"*
People diagnosed with MDR-TB	Social Protection, nutrition and economic	Lost income<br><br>Reduced productivity related to TB illness<br><br>Psychological stress	Economic barrier to treatment adherence	**FGD with people diagnosed with MDR-TB, 20-25 years age group, male:** *I used to be a conductor on a bus but I can't do any work now. I suffer headaches just going to the kitchen to cook rice. How can such a person work?"*

For peer review only

People diagnosed with MDR-TB	Social Protection, nutrition and economic	Lost income<br><br>Reduced productivity related to TB illness	Economic barrier to treatment adherence	**FGD with people diagnosed with MDR-TB, 40-45 years age group, male:** *"I used to work outside but after being affected by MDR-TB, I stopped going to work. I didn't earn money"*
People diagnosed with TB	Social Protection, nutrition and economic	Out-of-pocket expenses for accessing TB services<br><br>Borrowing money as coping strategies	Economic barrier to access and adhere to TB treatment	**FGD with people diagnosed with TB, 25-30 years age group, male:** *"I had difficulties [to pay money to access TB services] and wasn't able to go [to the clinic] for a month….I had to borrow money from my friends."*
People diagnosed with TB	Social Protection, nutrition and economic	Out-of-pocket expenses for accessing TB services<br><br>Selling assets as coping strategies	Economic barrier to treatment adherence	**FGD with people diagnosed with TB, 20-25 years age group, male:** *"I can't pay back the loan [I took out during treatment], which causes problems…..we might have to sell assets to pay back the loan."*
People diagnosed with MDR-TB	Social Protection, nutrition and economic	Inadequate financial support	Economic barrier to treatment adherence	**FGD with people diagnosed with MDR-TB, 20-25 years age group, male:** *"The TB hostel where I am staying assured us that they would give money but they still haven't [up to 20 months into treatment]."*
**Social protection and nutrition**				
**FGD**	**First order categories**	**Second order themes**	**Third order themes**	**Quote**
Civil society organization	Social Protection,	Provision of nutrition or cash incentives	Nutrition and or economic support	**FGD with civil society organization, 50-55 years age group, female:** "*When patients take medicine, they will feel weak but they will not have the facility of proper*

For peer review only

	nutrition and economic		to treatment adherence	*nutritious food. Thus, if certain amount of money is made available to them, then it will help them."*
Community leaders	Social Protection, nutrition and economic	Delivery mechanism and timing of incentives	Economic support to treatment adherence	**FGD with community leaders, 45-50 years age group, male:** *"The question is when to give the allowance. It would be better in the first phase [of treatment] because it is [most] valuable at the time one most needs it."*
People diagnosed with TB	Social Protection, nutrition and economic	Provision of economic and nutrition support<br><br>Prioritization of nutrition and economic support	Nutrition and or economic support to treatment adherence	**FGD with people diagnosed with TB, 30-35 years age group, male:** *"Government should provide nutritious food based on the economic status of patients……drug-sensitive patients should also be provided with an allowance based on their socio-economic condition."*

Category 3: Psychosocial barriers

**FGD**	**First order categories**	**Second order themes**	**Third order themes**	**Quote**
Female: diagnosed with TB	Psychosocial category	Stigma<br><br>Enacted stigma in the community<br><br>Perceived stigma<br><br>Lack of knowledge	Social barrier to adhere during treatment	**FGD with female: diagnosed with TB, 25-30 years age group, female:** *"I have not told anyone in my family about my disease because they will hate me. I fear that if my family or friends know about my disease, they'll start talking behind my back and not come near me."*

For peer review only

Civil society organization	Psychosocial category	Stigma<br><br>Enacted stigma in the community<br><br>Perceived stigma<br><br>Lack of knowledge	Social barrier to access and adhere during treatment	**FGD with civil society organization, 45-50 years age group, male:** *"People don't want to sit close to or make contact with someone with TB. They can act scared when they're near a patient with TB. Ironically, the same society promotes TB treatment and encourages people with TB to get themselves tested and treated."*
People diagnosed with TB	Psychosocial category	Stigma<br><br>Enacted stigma in the community<br><br>Perceived stigma<br><br>Lack of knowledge	Social barrier to access	**FGD with people diagnosed with TB, 30-35 years age group, male:** *"People may know they have symptoms of TB but are too ashamed to go to the healthposts. People can't say out loud that they have TB. TB is regarded as a big disease and people get criticized for having it. The community perceives a TB patient differently than a normal person due to lack of awareness. That's why it's difficult to end TB."*
Healthcare professional	Psychosocial category	Stigma<br><br>Enacted stigma in the community<br><br>Perceived stigma<br><br>Lack of knowledge<br><br>Limited social support	Social barrier to adhere to treatment	**FGD with TB healthcare professional, 30-35 years age group, male:** *"Some people have very low social support, including from their family. Those people are at risk of not taking a full course of TB treatment."*

People diagnosed with MDR-TB	Psychosocial category	Psychological impact<br><br>Stress, anxiety, and depression	Psychological barrier to treatment adherence	**FGD with people diagnosed with MDR-TB, 45-50 years age group, male:** *"After seeing the healthcare workers, I felt angry and depressed. I didn't want to stay with others, not even with my family. I just sat alone."*
People diagnosed with MDR-TB	Psychosocial category	Psychological impact<br><br>Stress, anxiety, and depression	Psychological barrier to treatment adherence	**FGD with people diagnosed with MDR-TB, 20-25 years age group, male:** *I felt depressed at first. No one in my clan had suffered from this disease. I wondered how I'd got affected."*
People diagnosed with MDR-TB	Psychosocial category	Psychological impact<br><br>Stress, anxiety, and depression	Psychological barrier to treatment adherence	**FGD with people diagnosed with MDR-TB, 45-50 years age group, male:** *"I wanted to die. One of my friends [with MDR-TB] committed suicide after 16 months [of treatment]."*
**Category 3b: Psychosocial facilitators**				
**FGD**	**First order categories**	**Second order themes**	**Third order themes**	**Quote**
emale: diagnosed with TB	Psychosocial category	Family care and support	Mutual support as facilitator to treatment	**FGD with female: diagnosed with TB, 40-45 years age group, female:** *"When I restarted taking medicines, it was very difficult for me. My husband helped me at that time and, slowly, I started getting better."*

For peer review only

People diagnosed with MDR-TB	Psychosocial category	Peer support and motivation	Mutual or social support as facilitator to treatment	**FGD with people diagnosed with MDR-TB, 40-45 years age group, male:** *"My friends and the people in my village told me TB is normal disease and encouraged me that if I took my medicine, I'd be alright."*

For peer review only

Category 4: Healthcare system, TB diagnosis and care delivery

Category 4a: Healthcare system, TB diagnosis and care delivery barriers				
**FGD**	**First order categories**	**Second order categories**	**Third order categories**	**Quote**
TB healthcare professional	Healthcare system, TB diagnosis and care delivery	Delay in diagnosis<br><br>Self-perception of disease and choice of treatment<br><br>Repeated visits for diagnosis<br><br>Inadequate referral mechanisms	Convoluted pathway to diagnosis as barrier to access	**FGD with TB healthcare professional, 45-50 years age group, male:** *"When people feel sick [with TB symptoms], they start by using herbal medication at home. When they do not get relief from that medication, they go to Dhami/Jhakri [traditional healers], then the pharmacy, then to a government clinic. So, from the start of their illness to getting the right treatment, is around 1-2 months."*
TB healthcare professional	Healthcare system, TB diagnosis and care delivery	Inadequate treatment regulation<br><br>Under-recognition of TB symptoms<br><br>Inadequate referral mechanisms	Unregulated policy for medicines distribution	**FGD with TB healthcare professional, 55-60 years age group, male:** *"If I have a cough, the local pharmacy will give me amoxicillin. [If I continue to cough] I might get a more expensive antibiotic like cefixime. If I tell the pharmacist that I am coughing up blood - a clear sign in a place like Nepal that this could be TB - I might get some bigger, more expensive antibiotics but the thought would not be about tuberculosis. The pharmacist should tell me to get checked for TB but instead I will get sold antibiotics. There are thousands of pharmacies [like this] in South Asia."*

Community Mobilizers	Healthcare system, TB diagnosis and care delivery	Geographical inaccessibility to reach TB centers<br><br>Natural disasters<br><br>Centralised location of health facilities	Geographical barriers to access TB diagnosis and care	**FGD with Community Mobilizers, 30-35 years age group, female:** *"In rainy season, flooding makes it difficult for patients to go and get medicine and [health posts] are inaccessible"*
Community Mobilizers	Healthcare system, TB diagnosis and care delivery	Geographical inaccessibility to reach TB centers<br><br>Poor quality of road and transportation network	Geographical barriers to access TB diagnosis and care	**FGD with Community Mobilizers, 20-25 years age group, male:** *"One of the main reasons for not taking medicine is because the health posts are far away….and difficult to reach….especially when the rivers get bigger and wider; they can sweep people away. If the health post was nearer, it would be easier."*
Civil society organization	Healthcare system, TB diagnosis and care delivery	Poor healthcare services<br><br>Limited or inconvenient opening times of health facilities	Inadequate services from health centers as barrier to adhere to treatment	**FGD with civil society organization, 50-55 years age group, female:** *"If we don't go on time, we don't meet the doctor. The patients must reach the hospital clinic before 11 and take a ticket.*
Community leaders	Healthcare system, TB diagnosis and care delivery	Geographical inaccessibility to reach TB centers<br><br>Poor quality of road and transportation network	Geographical barriers to access TB diagnosis and care	**FGD with community leaders, 35-40 years age group, male:** *"The health facilities are far and also may be the difficult road."*

For peer review only

Community leaders	Healthcare system, TB diagnosis and care delivery	Geographical inaccessibility to reach TB centers	Geographical barriers to access TB diagnosis and care	**FGD with community leaders, 45-50 years age group, male:** *"They are far from the access of Health facilities. Due to this reason they do not get medicine regularly."*
People diagnosed with TB	Healthcare system, TB diagnosis and care delivery	Poor healthcare services<br><br>Limited human resources	Inadequate services from health centers as barrier to adhere to treatment	**FGD with people diagnosed with TB, 25-30 years age group, male:** "*There are no health workers. It's such a small hospital [where I attend for TB medicine], there are five employees but four of them don't work. When I go to find and take my medicines, they say they don't know and ask me to look for it myself. The main doctor is in a meeting all the time, he doesn't know."*
TB healthcare professional	Healthcare system, TB diagnosis and care delivery	Poor healthcare services	Inadequate patient centric care as barrier to treatment adherence	**FGD with TB healthcare professional, 55-60 years age group, male:** *"If we try to put ourselves in the patient's shoes, be a TB patient, do you think it would be possible for us to visit the health centre for six months? Ruin our careers and everything to attend the health centre? We need to implement innovative ideas to support treatment adherence that are patient-centred rather than program-centred."*
People diagnosed with TB	Healthcare system, TB diagnosis and care delivery	Poor TB service delivery	Care not being patient-centred acting as barrier	**FGD with people diagnosed with TB, 20-25 years age group, male:** *"I stayed for one hour in queue and then waited a long time for*

			to treatment adherence	*treatment, spent money, and got a delayed report."*
Female: diagnosed with TB	Healthcare system, TB diagnosis and care delivery	Poor TB service delivery	Care not being patient-centred acting as barrier to treatment adherence	**FGD with female: diagnosed with TB, under 20 years age group, female:** *"Getting a delayed test report was a barrier for me, we had to come and go [to health facilities] for 4 days."*
People diagnosed with MDR-TB	Healthcare system, TB diagnosis and care delivery	Poor TB service delivery	Care not being patient-centred acting as barrier to treatment adherence	**FGD with people diagnosed with MDR-TB, 45-50 years age group, male:** *"Our community mobiliser told us to go health post and they will administer the injection [injectable medication for TB]. I went but they told me that they couldn't give me an injection at that time."*
Community leaders	Healthcare system, TB diagnosis and care delivery	Poor TB service delivery	Care not being patient-centred acting as barrier to treatment adherence	**FGD with community leaders, 45-50 years age group, male:** *"The behaviour of NTP healthcare workers needs to change. There's a difference between saying 'your father' and 'your respected father' isn't it? Imagine how bad an ill person will feel….when spoken to in an ill-mannered way; the speech will feel like an arrow."*
TB healthcare professional	Healthcare system, TB diagnosis and care delivery	Poor TB service delivery	Care not being patient-centred acting as barrier to treatment adherence	**FGD with TB healthcare professional, 55-60 years age group, male:** *"The perception of lack of patient friendly services was also acknowledged as a problem in the NTP and patient FGDs and that although there are 4200 DOTS centres and many microscopic and GeneXpert centres, many are not patient*

For peer review only

				friendly and this can contribute to delayed initiation of treatment."
People Diagnosed with TB	Healthcare system, TB diagnosis and care delivery	Poor TB service delivery	Care not being patient-centred acting as barrier to treatment adherence	**FGD with people diagnosed with TB, 25-30 years age group, male:** *"They don't ask anything. They just give medicines and give a tick mark in the card. It is just this at our place. They make a register. But if the main doctor [health worker] is not there, they don't even do it. They ask to take medicines by ourselves. In our village, we are asked to take medicines from the box."*
Category 4b: TB diagnosis and care delivery facilitators				
**FGD**	**First order categories**	**Second order themes**	**Third order themes**	**Quotes**
Civil society organization	Healthcare system, TB diagnosis and care delivery	Standardization of TB treatment	Proper regulation and management of TB services delivery	**FGD with civil society organization, 45-50 years age group, male:** *"If TB medicines are only available from registered governmental health facilities, people will take them from there."*

For peer review only

People diagnosed with TB	Healthcare system, TB diagnosis and care delivery	Standardization of TB treatment	Proper regulation and management of TB services delivery	**FGD with people diagnosed with TB, 30-35 years age group, male:** *"The main point is TB medicine is available in pharmacies and those who can pay will get it because pharmacies want money. Pharmacies don't care much about treatment and cure….or if patients take medicines at the right time or at all. That is why….TB medicines should only be provided under the supervision of government health facilities only. This will help to control TB."*
People diagnosed with MDR-TB	Healthcare system, TB diagnosis and care delivery	Improved access to TB diagnostic and treatment services	Provision of community based healthcare services and patient-centred care	**FGD with people diagnosed with MDR-TB, 40-45 years age group, male:** *"What I like the most is they come to our houses to collect sputum."*
TB healthcare professional	Healthcare system, TB diagnosis and care delivery	Availability of diagnostic services	Early diagnosis of TB	**FGD with TB healthcare professional, 30-35 years age group, male:** *"GeneXpert, X-ray and symptoms are three things that the program is focusing on."*

For peer review only

Community leaders	Healthcare system, TB diagnosis and care delivery	Improved access to TB diagnostic and treatment services	Provision of community based healthcare services and patient-centred care	**FGD with community leaders, 45-50 years age group, male:** "We conduct screening camps for other diseases so why can't we do screening camp for tuberculosis? In the past, NGOs collected sputum from households where there was a patient on the basis of exposure history in family members. If we could collect and screen by understanding the patient's location that easier for them or visiting their workplace, it might increase the number of cases identified."
Female: diagnosed with TB	Healthcare system, TB diagnosis and care delivery	Improved access to TB diagnostic and treatment services	Provision of community based healthcare services and patient-centred care	**FGD with female: diagnosed with TB, 45-50 years age group, female:** *"It's good to bring medicine at home. Sometimes, we have work at the time when we have to go to take medicines."*
Female: diagnosed with TB	Healthcare system, TB diagnosis and care delivery	Good behavior of healthcare providers	Patient-centred care	**FGD with female: diagnosed with TB, 60-65 years age group, female:** *"The doctor treated me like a normal patient, cared for me, asked how I was feeling and gave me medicine."*

For peer review only

Community Mobilizers	Healthcare system, TB diagnosis and care delivery	Good behavior and communication between patient and healthcare providers	Patient-centred care	**FGD with Community Mobilizers, 40-45 years age group, male:** *"We provide time to patients….meet them on their way to work or when coming back from work…..they are in constant contact with us…..we consult them properly…they trust us more [than doctors]."*
Female: diagnosed with TB	Healthcare system, TB diagnosis and care delivery	Proper communication between patient and healthcare providers	Patient-centred care	**FGD with female: diagnosed with TB, 45-50 years age group, female: "***Doctor told that medicines should be taken for six months completely."*
TB healthcare professional	Healthcare system, TB diagnosis and care delivery	Standardization of TB treatment and referral services	Proper regulation and management of TB services delivery	**FGD with TB healthcare professional, 30-35 years age group, male:** *"Government hospitals should mandate pharmacies and private providers to refer people with TB to government services but it was noted that legislation was insufficient."*
People diagnosed with TB	Healthcare system, TB diagnosis and care delivery	Standardization of TB treatment and referral services	Proper regulation and management of TB services delivery	**FGD with people diagnosed with TB, 30-35 years age group, male: "***TB medicines should only be provided under the supervision of government health facilities only. This will help to control TB."*

For peer review only

TB healthcare professional	Healthcare system, TB diagnosis and care delivery	Decentralization of advanced diagnostic services in	Expansion of advanced diagnostic services	**FGD with TB healthcare professional, 55-60 years age group, male:** *"Decentralization of GeneXpert had revolutionized the TB diagnosis in the hills of Nepal to do better diagnosis and find MDR TB roll out GeneXpert."*
TB healthcare professional	Healthcare system, TB diagnosis and care delivery	Prioritization of vulnerable groups for increased diagnosis	Strategies and policies for TB diagnosis	**FGD with TB healthcare professional, 55-60 years age group, male: "***I think we have to change our strategy of active case finding, based on the level of vulnerability and the level of risk that a person can be exposed to. If we are following the same strategy for prisoners that we are following in the community, we might not find many cases."*

For peer review only

Supplementary File II: Healthcare system, TB diagnosis and care delivery barriers and facilitators

During thematic analysis, it was noted that the FGD participants identified "higher level" barriers and facilitators to accessing and engaging with TB services including governmental policy, political commitment, public-private mix, and healthcare infrastructure that would not be directly modifiable through a socioeconomic support intervention for TB-affected households. While important and informative, the research team felt that such themes could not practically be addressed by – and hence would not directly inform the design of - a discrete socioeconomic support package for TB-affected households for future trial evaluation. Here, the perceived higher level barriers and facilitators are mapped to Category IV "Healthcare system, TB diagnosis and care delivery" of the adapted WHO treatment adherence framework.[1] These barriers and facilitators are summarized in Figure 2 of the main article and expanded on in more detail below.

Theme: Healthcare system, TB diagnosis and care delivery barriers

Across FGDs, participants reported limited staffing at health centers, poor geographical access, unsatisfactory quality of the TB services and multiple visits in public and private health centers for diagnosis and treatment as the major barriers to accessing and engaging with TB treatment. Participants mentioned that the incorrect diagnosis of TB caused long and convoluted journey during the diagnosis and treatment, including a public-private (PPM) mix of hospitals and clinics and pharmacies before reaching NTP diagnostic and treatment services:

> **FGD with female diagnosed with TB, 25-30 years age group, female:** *"For many times, I went to hospitals and bought medicines. My health was worsening. A doctor prescribed medicines for increased blood pressure, I had those medicines. Also, later another doctor prescribed me medicines for gastritis. I had those medicines as well. None of the medicines made me feel better. It took a long time until I was diagnosed [of TB] in a government hospital."*

The NTP stakeholders mentioned that the pharmacy services were perceived to be compromised because of the practice of prescribing antibiotics without suitable clinical or microbiological confirmation of infection. Moreover, the practice of changing to new and more expensive antibiotics was noted if the disease wasn't cured. However, such services were noted to be thriving and supported a culture of, as one NTP stakeholder noted, *"medicine dependence in South Asia"*.

> **FGD with NTP stakeholders, 55-60 years age group, male:** *"If I have a cough, the local pharmacy will give me amoxicillin. [If I continue to cough] I might get a more expensive antibiotic like cefixime. If I tell the pharmacist that I am coughing up blood - a clear sign in a place like Nepal that this could be TB - I might get some bigger, more expensive antibiotics but the thought would not be about tuberculosis. The pharmacist should tell me to get checked for TB but instead I will get sold antibiotics. There are thousands of pharmacies [like this] in South Asia."*

Participants from the FGD with NTP stakeholders raised concerns about the risk of such antibiotic practices driving increasing rates of drug-resistant TB (DR-TB) and multidrug-resistant TB (MDR-

For peer review only

TB) in Nepal. The practice of seeking private care initially was felt to be commonplace among those of higher socioeconomic position because of sufficient household resources coupled with a perception of private services being quicker and/or higher quality than public services and more likely to help them to become cured. Nevertheless, poorer households were reported to visit cheaper, independent private healthcare providers, some of whom were reported to have no or limited formal training, in their local village. Ultimately, it was reported across FGDs that the decision to seek private care and the level of quality of private care provided was dictated by money.

> **FGD with civil society organization, 45-50 years age group, male:** *"The majority of the patients go to medical [pharmacy]. If they have to go to district [larger referral hospital], then the expenses will be higher. If they attend health centres, the expense depends on the distance."*

Geographical barriers such as long distances to reach health facilities, weather conditions such as rain, natural disasters (landslides and flooding), difficult roads and limited transportation options were discussed in depth across FGDs. These barriers were perceived as hampering access to TB diagnosis, impeding engagement with TB treatment and DOTS, and had a knock-on impact on access to broader health services.

The participants in FGDs with community stakeholders and people with TB perceived restricted opening hours, delayed opening, limited time available for clinical investigation [in hospitals], and prolonged waiting times for consultation and receiving reports as barriers to both TB diagnosis and treatment. Community stakeholders reported that this was especially important for labourers given the competing demands to earn money during peak labour hours or seek healthcare during clinic opening hours. These barriers were compounded by issues relating to staff lateness and staff shortages or absence at healthcare centers that provided TB services:

> **FGD with people diagnosed with TB, 25-30 years age group, male:** "*There are no health workers. It's such a small hospital [where I attend for TB medicine], there are five employees but four of them don't work. When I go to find and take my medicines, they say they don't know and ask me to look for it myself. The main doctor is in a meeting all the time, he doesn't know."*

Moreover, participants perceived that there was negative behaviour of healthcare workers towards people seeking TB diagnosis or who were taking TB treatment as a notable barrier and disincentive to attend. There was a perception that some NTP staff at health centers took little interest in people with TB. A participant with TB disease mentioned that the healthcare workers at the DOTS center only provide medicines and put a tick mark in the treatment card or sometimes even proper recording is missed in the card. Another participant with TB also shared his experience during his treatment where he was often told to take medicines from the shelf at the DOTS center. There were also reports of discourteous consultations

During FGDs with people with MDR-TB, room conditions, atmosphere, and quality of TB services at MDR-TB centers were reported as not being patient-friendly and a significant barrier to engagement. Apart from waiting times, issues raised included crowded waiting rooms and

inadequacies in cleanliness and hygiene of clinics and hospitals. Although people with MDR-TB reported supportive care and staff at the DR-TB hostels in which some were managed during their treatment, others reported that the facilities were not as expected. One participant describing their room to have been *"colder than snow"* and another shared that the substandard conditions of the hostel rooms made him feel even worse and so he did not remain there. The perception of lack of patient friendly services was also acknowledged as a problem in the FGDs with NTP stakeholders and people with TB:

Theme: Healthcare system, TB diagnosis and care delivery facilitators

During the FGD with NTP stakeholders, PPM was discussed in detail and, specifically, integration of the public and private sector TB services as a potential facilitator. Both NTP stakeholders and people with TB cited that government hospitals should mandate pharmacies and private providers to refer people with TB to government services. However, it was noted that current legislative powers were insufficient to realise this:

> **FGD with NTP stakeholders, 30-35 years age group, male:** *"We can regulate the health professionals but we cannot regulate the pharmacies….where the majority of TB cases are going and taking pills."*

It was noted across FGDs that outreach and decentralized community-based services that incorporate use of advanced diagnostic tools would be beneficial to reach healthcare facilities. NTP stakeholders reported that decentralization of GeneXpert had revolutionized TB diagnosis in the hills of Nepal, increasing TB case detection while promptly identifying people with DR-TB and MDR-TB. The NTP stakeholders mandated roll out of GeneXpert across Nepal and suggested integration of GeneXpert with targeted active-case finding strategies such as TB camps and door-to-door campaigns. The participants also suggested community screening, which focused on reaching underserved, vulnerable people at high risk of TB, was appropriate. People with MDR-TB reported that such active case finding strategies had been well received:

> **FGD with people with MDR-TB, 40-45 years age group, male:** *"What I like the most is they come to our houses to collect sputum."*

In addition to reports of negative behaviour from NTP staff during treatment, participants with TB also reported experiences of positive behaviour towards them as a motivating factor, which had encouraged them to take their medicines regularly. Supportive behaviour coupled with education about medication – including side effects, importance of adherence, and duration - during initial consultations was seen as an enabler for treatment engagement and adherence. During the FGD with NTP stakeholders, it was mentioned that providing adequate time to patients, proper counseling, and consultation enable people with TB disease to complete their treatment and get cured. This was perceived to be enhanced by community outreach activities by community mobilisers and female community health volunteers to ensure patients had the best opportunity to achieve treatment success:

> **FGD with female with TB, 60-65 years age group, female:** *"The doctor treated me like a normal patient, cared for me, asked how I was feeling and gave me medicine."*

For peer review only

Furthermore, during the FGD with people with TB, participants mentioned the necessity of patient-centric treatment services such as taking TB-medications at home instead of daily visits to the DOTS centers, which is often time consuming.

FGD with female diagnosed with TB, 45-50 years age group, female: *"It's good to bring medicine at home. Sometimes, we have work at the time when we have to go to take medicines."*

References

1. Weaver MS, Lönnroth K, Howard SC, Roter DL, Lam CG. Interventions to improve adherence to treatment for paediatric tuberculosis in low- and middle-income countries: a systematic review and meta-analysis. Bull World Health Organ. 2015;93(10):700-711B.

For peer review only

Wellcome Open Research

Wellcome Open Research 2020, 5:19 Last updated: 11 SEP 2020

Check for updates

STUDY PROTOCOL

REVISED Research protocol for a mixed-methods study to characterise and address the socioeconomic impact of accessing TB diagnosis and care in Nepal [version 2; peer review: 2 approved]

Kritika Dixit[1,2], Bhola Rai[1], Tara Prasad Aryal[1], Gokul Mishra[1], Noemia Teixeira de Siqueira-Filha[3], Puskar Raj Paudel[4], Jens W. Levy[4], Job van Rest[4], Suman Chandra Gurung[1,3], Raghu Dhital[1], Olivia Biermann[2], Kerri Viney[2], Knut Lonnroth[2], S Bertel Squire[3,5], Maxine Caws[1,3], Tom Wingfield[2,3,5]

[1]Birat Nepal Medical Trust, Lazimpat Road, Lazimpat, Ward No 2, Box 20564, Kathmandu, Nepal
[2]Social medicine, Infectious diseases, and Migration (SIM) Group, Department of Public Health Sciences, Karolinska Institute, Solnavägen 1, 171 77 Solna, Stockholm, Sweden
[3]Departments of International Public Health and Clinical Sciences, Liverpool School of Tropical Medicine, Pembroke Place, Liverpool, L3 5QA, UK
[4]KNCV Tuberculosis Foundation, Postbus 146, 2501 CC Den Haag, The Netherlands
[5]Tropical and Infectious Disease Unit, Liverpool University Hospitals NHS Foundation Trust, Prescot Street, Liverpool, L7 8XP, UK

For peer review

v2 **First published:** 05 Feb 2020, **5**:19
https://doi.org/10.12688/wellcomeopenres.15677.1

Latest published: 16 Jun 2020, **5**:19
https://doi.org/10.12688/wellcomeopenres.15677.2

Abstract

Background: WHO's 2015 End TB Strategy advocates social and economic (socioeconomic) support for TB-affected households to improve TB control. However, evidence concerning socioeconomic support for TB-affected households remains limited, especially in low-income countries.

Protocol: This mixed-methods study in Nepal will: evaluate the socioeconomic impact of accessing TB diagnosis and care (Project 1); and create a shortlist of feasible, locally-appropriate interventions to mitigate this impact (Project 2). The study will be conducted in the Chitwan, Mahottari, Makawanpur, and Dhanusha districts of Nepal, which have frequent TB and poverty.

The study population will include: approximately 200 people with TB (Cases) starting TB treatment with Nepal's National TB Program and 100 randomly-selected people without TB (Controls) in the same sites (Project 1); and approximately 40 key in-country stakeholders from Nepal including people with TB, community leaders, and TB healthcare professionals (Project 2).

Open Peer Review

Reviewer Status ✔ ✔

	Invited Reviewers	
	**1**	**2**
**version 2** (revision) 16 Jun 2020	✔ report	✔ report
**version 1** 05 Feb 2020	? report	? report

1. **Delia Boccia**, London School of Hygiene and Tropical Medicine, London, UK

2. **Priya B. Shete**, University of California San Francisco, San Francisco, USA

For peer review only - http://bmjopen.bmj.com/site/about/guidelines.xhtml

During Project 1, visits will be made to people with TB's households during months 3 and 6 of TB treatment, and a single visit made to Control households. During visits, participants will be asked about: TB-related costs (if receiving treatment), food insecurity, stigma; TB-related knowledge; household poverty level; social capital; and quality of life.

During Project 2, stakeholders will be invited to participate in: a survey and focus group discussion (FGD) to characterise socioeconomic impact, barriers and facilitators to accessing and engaging with TB care in Nepal; and a one-day workshop to review FGD findings and suggest interventions to mitigate the barriers identified.

Ethics and dissemination: The study has received ethical approval. Results will be disseminated through scientific meetings, open access publications, and a national workshop in Nepal.

Conclusions: This research will strengthen understanding of the socioeconomic impact of TB in Nepal and generate a shortlist of feasible and locally-appropriate socioeconomic interventions for TB-affected households for trial evaluation.

Keywords
Tuberculosis, poverty, catastrophic costs, socioeconomic support, social protection, healthcare access, Nepal

Any reports and responses or comments on the article can be found at the end of the article.

Corresponding author: Tom Wingfield (tom.wingfield@lstmed.ac.uk)

Author roles: Dixit K: Data Curation, Formal Analysis, Investigation, Project Administration, Visualization, Writing – Original Draft Preparation, Writing – Review & Editing; **Rai B**: Data Curation, Investigation, Project Administration, Writing – Review & Editing; **Prasad Aryal T**: Investigation, Project Administration, Writing – Review & Editing; **Mishra G**: Investigation, Methodology, Supervision, Writing – Review & Editing; **Teixeira de Siqueira-Filha N**: Data Curation, Formal Analysis, Investigation, Methodology, Project Administration, Writing – Original Draft Preparation, Writing – Review & Editing; **Raj Paudel P**: Data Curation, Project Administration, Writing – Review & Editing; **Levy JW**: Data Curation, Resources, Validation; **van Rest J**: Data Curation, Resources, Writing – Review & Editing; **Chandra Gurung S**: Investigation, Resources, Supervision, Writing – Original Draft Preparation, Writing – Review & Editing; **Dhital R**: Investigation, Project Administration, Resources, Writing – Original Draft Preparation, Writing – Review & Editing; **Biermann O**: Methodology, Writing – Review & Editing; **Viney K**: Methodology, Supervision, Writing – Original Draft Preparation, Writing – Review & Editing; **Lonnroth K**: Conceptualization, Methodology, Supervision, Writing – Original Draft Preparation, Writing – Review & Editing; **Squire SB**: Conceptualization, Methodology, Supervision, Writing – Original Draft Preparation, Writing – Review & Editing; **Caws M**: Conceptualization, Formal Analysis, Funding Acquisition, Investigation, Methodology, Project Administration, Supervision, Writing – Original Draft Preparation, Writing – Review & Editing; **Wingfield T**: Conceptualization, Data Curation, Formal Analysis, Funding Acquisition, Investigation, Methodology, Project Administration, Supervision, Validation, Visualization, Writing – Original Draft Preparation, Writing – Review & Editing

Competing interests: No competing interests were disclosed.

Grant information: This work was supported by grants to TW from the Wellcome Trust [209075] and the Academy of Medical Sciences, UK.
The funders had no role in study design, data collection and analysis, decision to publish, or preparation of the manuscript.

Copyright: © 2020 Dixit K *et al*. This is an open access article distributed under the terms of the Creative Commons Attribution License, which permits unrestricted use, distribution, and reproduction in any medium, provided the original work is properly cited.

How to cite this article: Dixit K, Rai B, Prasad Aryal T *et al.* **Research protocol for a mixed-methods study to characterise and address the socioeconomic impact of accessing TB diagnosis and care in Nepal [version 2; peer review: 2 approved]** Wellcome Open Research 2020, **5**:19 https://doi.org/10.12688/wellcomeopenres.15677.2

First published: 05 Feb 2020, **5**:19 https://doi.org/10.12688/wellcomeopenres.15677.1

For peer review only - http://bmjopen.bmj.com/site/about/guidelines.xhtml

Wellcome Open Research 2020, 5:19 Last updated: 11 SEP 2020

REVISED **Amendments from Version 1**

In response to the reviewers' constructive comments, we have updated the following:

1. Provided more detail concerning coding methods for Project 2
2. Provided clarification concerning the selection and recruitment of controls, including limitations and weaknesses in this method
3. Included a conceptual framework that informed the study design and analysis (new Figure 3)
4. Included a logic model on the development of the socioeconomic intervention, in line with MRC methods (new Figure 4)
5. Clarified on data collection relating to social protection

These comments have been helpful in improving the protocol and we are grateful to the reviewers for their time and expertise.

Any further responses from the reviewers can be found at the end of the article

Introduction

Tuberculosis (TB) disease, which kills 1.5 million people annually, is driven by poverty[1]. Having TB disease can also worsen impoverishment through loss of income and costs of accessing care[2–5]. Such costs can become "catastrophic", leading patients to abandon treatment, develop drug-resistance, and die[6]. WHO's 2015 End TB Strategy advocates elimination of catastrophic costs and provision of socioeconomic support for TB-affected households[5]. Nevertheless, there is minimal evidence concerning the ideal interventions to realise this policy change[6–10]. This research will generate preliminary evidence to fill this knowledge gap in a low-income country: Nepal. The findings will inform a randomised controlled trial of socioeconomic support for TB-affected households in Nepal.

Research from Peru has demonstrated that the severe socioeconomic impact of TB can lead to catastrophic costs (defined by WHO as >20% of a household's annual income)[6]. The same team also demonstrated that socioeconomic interventions for TB-affected households can mitigate catastrophic costs (Figure 1a), improve TB preventive therapy uptake (Figure 1b), and increase TB treatment success (Figure 1c)[11–15].

However, despite these encouraging findings, Peru is a middle-income country with a strong TB program and existing national cash-transfer schemes. To make this research replicable and applicable in diverse settings, interventions similar to those in Peru need to be adapted to other country contexts, especially low-income countries (LICs) with less developed social protection schemes and high TB burden. One such country is Nepal.

The estimated incidence of TB in Nepal is 154/100,000 people with 45,000 cases of TB being notified. Amongst these cases, there were 6,800 TB-related deaths. Multi-drug resistance (MDR) rates were 2.2% in new cases and 15% among retreatment cases. Importantly, despite good treatment success rates overall of more than 90% reported in Nepal, there remain shortcomings in TB care: treatment success rates were 70% for patients with MDR-TB and only 9% for those with HIV-TB co-infection; and accessibility of TB care remains low with treatment coverage of 70%[16].

The poor treatment coverage in Nepal may, in part, be due to the financial impact on TB-affected households, which is estimated to be high[17]. Responding to this estimated burden, the Nepal National TB Programme (NTP) national strategic plan for 2016–2021 identifies provision of a support package to TB-affected households (specifically targeting MDR-TB-affected households) as a priority aim for the country with the goal of reducing catastrophic costs. There is currently limited evidence concerning the potential acceptability, impact, or cost-effectiveness of such a package, with which to inform and guide this policy decision. A cohort study has suggested that, during their illness, TB patients in Nepal experience decreased income and the total costs of accessing free TB treatment equate to nearly one quarter of annual household income[17]. Further studies in Nepal have shown that clinic fees make up the largest proportion of direct costs and that TB patients who are poorer, are migrants, or are from rural areas, experience a disproportionate burden of total costs[18–20]. It is vital that vulnerable TB patient households most at risk of incurring TB-related costs are identified in order to prevent and cure TB and mitigate further impoverishment and its consequences. In non-randomized studies, incurring higher TB-related costs and not receiving education about TB have both been found to be associated with worse TB treatment adherence and adverse TB treatment outcomes[18,21]. Therefore, the financial impact of having TB disease in Nepal constitutes a challenge to achieving TB control and elimination.

To date, no study of TB-affected households' costs in Nepal has been performed using the standardised global methodology for measuring costs related to accessing and engaging with TB care: the WHO TB Patient Costs Survey[22,23]. The studies above collected data at only one time point rather than repeated time points (e.g. cross-sectional rather than longitudinal) and did not robustly analyse the socioeconomic position, nutritional status, coping strategies, or linkage to social protection of TB-affected households. There have been no trials of socioeconomic support for TB-affected households in Nepal but formative qualitative analysis and a non-randomized pilot interventional study (offering education and financial support to patients with MDR-TB) suggested improved treatment outcomes[24].

However, studies reporting quantitative data alone will be insufficient to influence and change policy. Interaction with and among stakeholders has been described as the key facilitator for knowledge translation and evidence-informed health policymaking[25]. These interactions can include policy dialogues between stakeholders to deliberate on a priority topic. Therefore, complementary to quantitative data, the focus group

For peer review only - http://bmjopen.bmj.com/site/about/guidelines.xhtml

Wellcome Open Research 2020, 5:19 Last updated: 11 SEP 2020

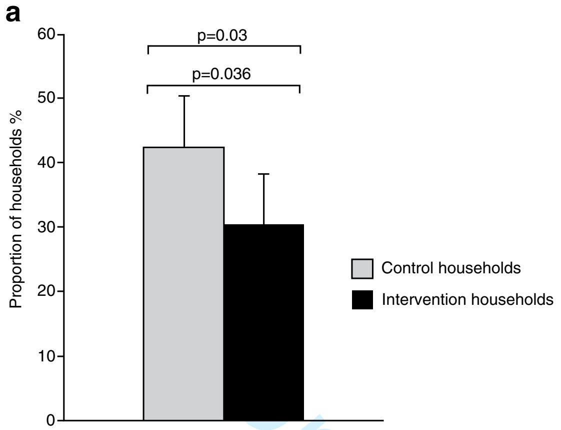

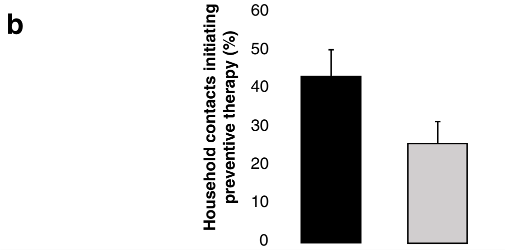

		■ Contacts from intervention household (n=206)	□ Contacts from control household (n=204)
Proportions	n<br>% (95% CI)	91<br>44 (37-51)	53<br>26 (45-61)
Crude analysis	OR (95% CI)<br>P value	2.2 (1.4-3.3)<br><0.001	
Adjusted analysis	aOR (95% CI)<br>P value	2.2 (1.1-4.1)<br>0.02	

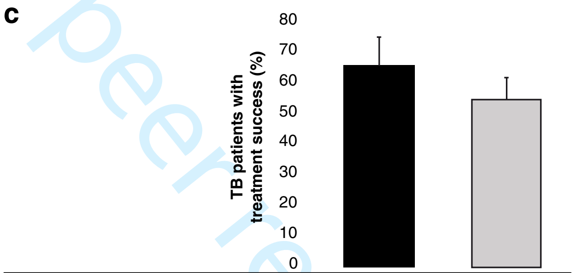

		■ Intervention households (n=135)	□ Control households (n=147)
Proportions	n<br>% (95% CI)	87<br>64 (56-72)	78<br>53 (45-61)
Likelihood of treatment success	OR (95% CI)<br>P value	1.6 (1.0-2.6)<br>0.05	

Figure 1. **a**) Catastrophic costs incurred by intervention (n=135) and control (n=147) TB-affected households of Callao, Peru. **b**) Preventive therapy initiation in household contacts of intervention (n=206) and control (n=204) TB-affected households of Callao, Peru. **c**) TB treatment success in patients from intervention (n=135) and control (n=147) TB-affected households of Callao, Peru. Part **a**) has been reproduced with permission from Wingfield *et al.*[13] Parts **b**) and **c**) have been reproduced with permission from Wingfield *et al.*[15].

discussions (FGDs) and workshop detailed within this protocol will aim to expand a policy dialogue on socioeconomic support for TB-affected households among key stakeholders in Nepal, from people with TB to community leaders to TB healthcare professionals. Policy dialogues represent knowledge translation to support the integration of research evidence with tacit knowledge of local health policy-makers to: inform future policy decisions in often complex and dynamic contexts; and foster proactive collaboration[26,27]. These elements will be critical for successful implementation of any future interventions[28].

In summary, this mixed-methods research will complement and extend the existing knowledge base on social determinants and consequences of TB. More specifically, the culmination of the research will be the creation of a shortlist of feasible and locally-appropriate socioeconomic interventions for TB-affected households for future randomized controlled trial evaluation in Nepal.

Protocol

Ethical statement

Ethical approval was granted from the University of Liverpool, UK, research ethics committee in April 2018 (approval number 2436) and then the National Health Research Council of Nepal (NHRC) research ethics committee in May 2018 (approval number 320/2018).

Participant information leaflets will be provided and written informed consents will be obtained from all study participants for Project 1 Interviews (separate consent forms and information leaflets for patients and healthy controls), Project 2 Surveys, FGDs, and the workshop. These documents are available in the Extended Data section attachments[29].

All medical records obtained from the Nepal NTP will be kept confidential. Practically, through liaison with NTP Project Staff (as already organised for IMPACT-TB), the PM, PI, and

For peer review only - http://bmjopen.bmj.com/site/about/guidelines.xhtml

RA will photocopy patient records from the Nepal NTP TB register obscuring the patient's identifiable details. Photocopies will be marked with that patient's unique study number identifier. No individual patients will be identifiable from publications resulting from this study.

Study design

This mixed-methods study was funded by a Wellcome Trust Seed Award in Science (awards provided to early-career researchers to develop a novel idea that will go on to form part of a larger grant application) and will be divided into two complementary projects. Project 1 consists of a cohort study characterising the socioeconomic impact of TB on TB-affected households and a nested case-control study examining the social determinants of TB. Project 2 consists of a mixed quantitative-qualitative cross-sectional study using surveys, FGDs and a workshop to identify the barriers and facilitators to accessing and engaging with TB diagnosis and care in Nepal and suggesting potential interventions to mitigate the socioeconomic impact and improve access and engagement. The study will take place within the infrastructure of the larger EU-Horizon 2020 funded "IMPACT-TB" project, which is a study evaluating proven TB active case-finding (ACF) interventions in Nepal and Vietnam (grant 733174, http://www.impacttbproject.org/).

Primary aims

The primary aim of Project 1 is to evaluate the socioeconomic impact on TB-affected households of accessing and engaging with TB diagnosis and care in Nepal and compare that impact in people with TB identified through standard passive case finding (PCF) versus ACF. The primary aim of Project 2 is to collaborate with key stakeholders in Nepal to create a shortlist of potentially feasible and locally-appropriate socioeconomic interventions to mitigate this impact.

Secondary aims

The secondary aim of Project 1 is to compare the social determinants of TB (including socioeconomic position, housing situation, knowledge about TB, comorbidities, quality of life, food security, and social capital) in people with TB versus people without TB from the same districts. The secondary aim of Project 2 is to collate the opinions of key stakeholders from diverse sectors about barriers and facilitators to accessing and engaging with TB care in Nepal.

An additional aim across the study is to generate a policy dialogue and form a collaborative research network to support development and implementation of a future randomised control trial of socioeconomic support for TB-affected households in Nepal.

Study setting

The study will take place within the infrastructure of the larger IMPACT-TB study, which works with a well-established international non-governmental organisation, Birat Nepal Medical Trust (BNMT), to implement ACF activities including sputum-microscopy camps and roll-out of GeneXpert OMNI in four intervention and two control districts (with PCF only). The four districts are located in the central development region of Nepal and were selected for the IMPACT-TB project based on comparable populations and TB case detection rates (Figure 2).

The primary outcome of IMPACT-TB is the effect of ACF on early TB case detection and the study does not involve support packages for TB-affected households during treatment. This presents a unique opportunity for the complementary research described in this protocol to add value to IMPACT-TB, explore the socioeconomic impact of accessing TB care, and shortlist locally-driven strategies to reduce this impact.

Study population

Participant identification, recruitment and follow-up. During Project 1, approximately 200 people with TB (cases) consecutively recruited to Work Package 3 (WP3) of IMPACT-TB (Health Economic Analysis) in the four study sites will be further recruited to this study. 100 of these cases will have been diagnosed through ACF and 100 diagnosed through PCF. Whilst attending the NTP TB clinic, these people with TB will be invited to participate in this mixed-methods research with a separate written, informed consent (see Project 1 Interview Patient Consent Form, *Extended data*)[29]. During months three and six of their TB treatment, household visits will be conducted by BNMT project staff including community mobilisers.

Concurrently, 100 people without TB from the study sites (controls) will be invited to participate with written, informed consent (see Project 1 Interview Healthy Control Consent Form, *Extended data*)[29]. Due to constraints in study budget, duration, and field logistics, it will not be possible to age and sex match cases and controls nor to randomly select controls using geospatial or other household randomisation techniques. Therefore, a convenience sampling strategy was used. In order to be as widely representative of and generalisable to the background population as possible, following project team and community mobiliser discussions in the study sites, we opted to recruit control participants from diverse locations within the districts, which were attended by a broad demographic cross-section of the local population in terms of age, gender, and socioeconomic position. These diverse study site locations include: tea houses, primary healthcare centres, antenatal and immunization clinics, door-to-door visits following sputum camps (e.g. people who tested negative for TB), and public gathering places. Interviewers will visit the control recruitment locations at similar times of the morning and aim to consecutively recruit all individuals in attendance at that location. Similar numbers of participants from each recruitment location will be recruited until the sample size of 100 people without TB is reached. We acknowledge that convenience sampling may be associated with a higher likelihood of a non-generalisable control cohort than other techniques. To try to address this, the survey used to collect data from the controls has incorporated multiple questions concerning sociodemographic variables, including education level, occupation, amenities, and assets, from the most recent version of the Nepal Household Survey, which is publicly available. Descriptive

For peer review only - http://bmjopen.bmj.com/site/about/guidelines.xhtml

Wellcome Open Research 2020, 5:19 Last updated: 11 SEP 2020

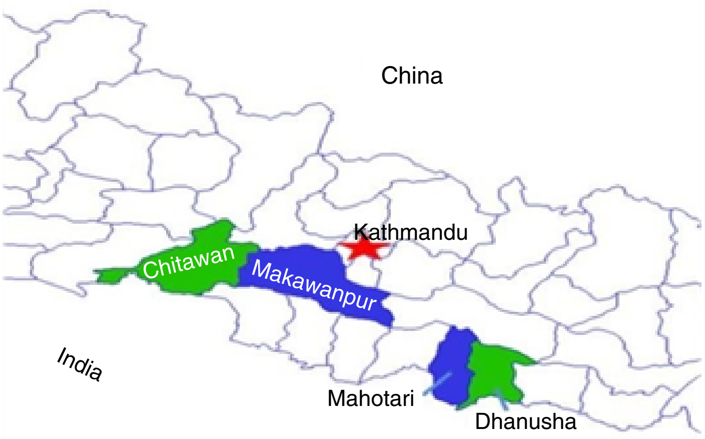

District	Population	Case Notification Rate (per 100,000 population)
Makwanpur	480,000	147
Chitwan	585,000	158
Dhanusha	823,000	100
Mahottari	681,000	131

Figure 2. Map, population, and TB case notification rate of the four study site districts. Data for population and TB cases notification rate shown is taken from the Nepal TB Centre 2018 report. The four study site districts are labelled and highlighted in blue and green.

analysis will evaluate whether the controls recruited are representative of the wider population in those districts through comparison of their sociodemographic data with respondents to the national survey in the same districts. Any differences between the control population and background population will then be highlighted transparently in corresponding research outputs and publications.

Project 1 inclusion criteria for Cases included: being a person with TB notified to the NTP and recruited to WP3 of the IMPACT-TB study; being aged 18 years or above; and giving verbal and written informed consent to participate. Project 1 exclusion criteria for Cases included: being under 18 years of age; being a person with TB not notified to the NTP and/or not recruited to WP3 of the IMPACT-TB study; being a person with TB notified to the NTP but with a recorded domiciliary address outside of the study site districts; and being unable or unwilling to give written and/or verbal informed consent to participate.

Project 1 inclusion criteria for Controls included: being 18 years or above with primary residence in the study site communities; not currently known to be a person with TB or have a member of the household currently known to be a person with TB (e.g. not diagnosed or notified or receiving TB treatment); and giving verbal and written informed consent to participate. Project 1 exclusion criteria for Controls included: being under 18 years of age; not having primary residence in the study site communities; known to be a person with TB or have a household member with TB currently (e.g. diagnosed and/or notified and/or receiving TB treatment); and being unable or unwilling to give verbal and written informed consent to participate.

To generate the population for Project 2, a literature review and desk-based scoping review will identify a list of key in-country stakeholders from Nepal from diverse groups including: civil-society representatives; community leaders; and TB healthcare professionals including NTP managers and multi-disciplinary staff. Approximately 50 stakeholders will be selected through purposive sampling and invited to participate in: a pre-FGD survey, FGD, and a one-day workshop. A subset of purposively sampled TB patients recruited to Project 1 (including those with multi-drug resistant TB) will be among the stakeholders invited to participate.

Project 2 inclusion criteria include: being aged 18 years or above; belonging to a stakeholder group as defined above and/or identified during scoping exercise; and being able and willing to provide verbal and written, informed consent. Exclusion criteria for Project 2 are not meeting the inclusion criteria and/or being a person with TB who has not yet taken two weeks of TB treatment or is otherwise considered to still be infectious (e.g. MDR-TB with positive sputum smear or culture).

For peer review only - http://bmjopen.bmj.com/site/about/guidelines.xhtml

Wellcome Open Research 2020, 5:19 Last updated: 11 SEP 2020

Sample size and statistical power. The sample size for Project 1 is approximately 200 Cases recruited to WP3 of the IMPACT-TB project and 100 Controls from the four study site districts. This sample size is opportunistic and pragmatic: related research suggests that data from 100 people with TB gives a representative spread of costs for a given context[30,31].

For Project 2, each FGD will consist of approximately eight stakeholders. The estimated number of FGDs at which information power (or saturation level) will be reached is six[12]. Thus, the sample size for Project 2 is approximately 40 stakeholder participants. We will invite 55 stakeholders as a contingency because we anticipate an attrition rate of 20–30% during the course of Project 2.

Given that this Seed Award research will be exploratory, preparatory (e.g. for the future trial), and does not include an intervention, no calculations of statistical power are required.

Study interventions

The study will not include any interventions or require any patient samples (e.g. blood / sputum / tissue).

Study activities

Broadly, the activities involved in the research will include interviews with Cases and Controls during household visits, and a pre-FGD survey, FGD, and workshop with key stakeholders.

During Project 1, the project team will support BNMT district coordinators, community mobilisers, and community volunteers to do household visits to approximately 200 Cases with TB (100 diagnosed through ACF and 100 through PCF) recruited to WP3 of the IMPACT-TB project. People with TB will receive two household visits, the first during month three of TB treatment and the second visit during month six of TB treatment (to correspond with treatment completion). For controls, a single household visit and interview will be done. The interviews will be structured (see Project 1 Interview, *Extended data*)[29] and gather data on: i) socioeconomic position, evaluated by a multi-dimensional poverty score[6,9,13,15] assessing dwelling characteristics, assets, and access to amenities; ii) household structure, including distribution of age, sex, and employment of household members; iii) food expenditure and security; iv) costs of engaging with TB care including direct costs (e.g. medicines, clinic visits, food, and travel) and indirect costs (e.g. lost income), which will be evaluated using an adapted version of WHO's TB Patient Costs Survey integrated into the interview[22,23]; v) coping strategies including dis-saving (e.g. selling assets), schooldays lost, and temporary income-generating activities; vi) TB-related knowledge including understanding of transmission, prevention, and treatment of TB; vii) psychosocial situation evaluated through questions relating to social capital, quality of life, and stigma (Controls without TB will not be asked any questions about the impact that having TB disease has on their psychosocial situation); and viii) access and uptake of existing social protection schemes (whether TB-specific or TB-inclusive) and support for TB-affected households, evaluated through use of both closed ranking and open free-text questions to establish what socioeconomic and other support people with TB and their households receive or would like to receive.

Prior to implementation of household visits, the questionnaire will be translated from English into Nepali and then back-translated into English. It will be assessed by members of the study team and BNMT implementation staff before being piloted in approximately 10 patients. The questionnaire may subsequently be refined and questions deleted or added, depending on the pilot outcomes.

In addition to the above, Cases' TB treatment outcomes will be collated from NTP and IMPACT-TB data and their association with interview responses analysed. This will provide an exploratory analysis of the association of socioeconomic position, socioeconomic impact of having TB, and TB treatment outcomes. A comparison of the socioeconomic position of households of Cases and Controls will also be made.

Project 2 will use and develop mixed methods research techniques[10–15] to conduct a pre-FGD survey, FGDs, and workshop with approximately 40 key stakeholders in Nepal to identify the socioeconomic impact, barriers and facilitators to accessing and engaging with TB diagnosis and care. To inform the design of the FGDs and workshop, we created a conceptual framework for the barriers and facilitators to TB diagnosis and care, which was adapted from a World Health Organisation framework for medication adherence (Figure 3)[32].

A short individual pre-FGD survey (see Project 2 Survey, *Extended data*)[29] will be provided to participants prior to the initiation of an FGD. The survey will detail: participants' demographics; the stakeholder group to which the participant belongs; and their opinions on community, patient, health system, existing social protection schemes, and wider obstacles to achieving successful TB treatment outcomes.

The FGDs will be semi-structured and incorporate open-ended questions concerning barriers and facilitators to accessing and engaging with TB care in Nepal, and existing platforms and potential opportunities to mitigate these barriers including social protection schemes (see Project 2 Focus Groups, *Extended data*)[29]. The FGDs will be conducted with separate groups of approximately eight key stakeholders. Stakeholders will be invited to participate according to their background (e.g. people with TB will be asked to participate in one FGD, and TB healthcare professionals will be asked to participate in another separate FGD). Towards the end of each FGD, participants will be asked to privately rank the top three most important barriers or facilitators to accessing TB diagnosis and care identified by the group during the FGD, and these responses will be collated for each FGD and across FGDs. The FGDs will be moderated by members of the project team trained in qualitative methods including conducting FGDs. The discussions will be audio recorded in Nepali language, translated into English, and back-translated by a translator who is not part of the project team. Each FGD group will be asked to elect a representative to feed their group's outputs back at the subsequent workshop.

For peer review only - http://bmjopen.bmj.com/site/about/guidelines.xhtml

Wellcome Open Research 2020, 5:19 Last updated: 11 SEP 2020

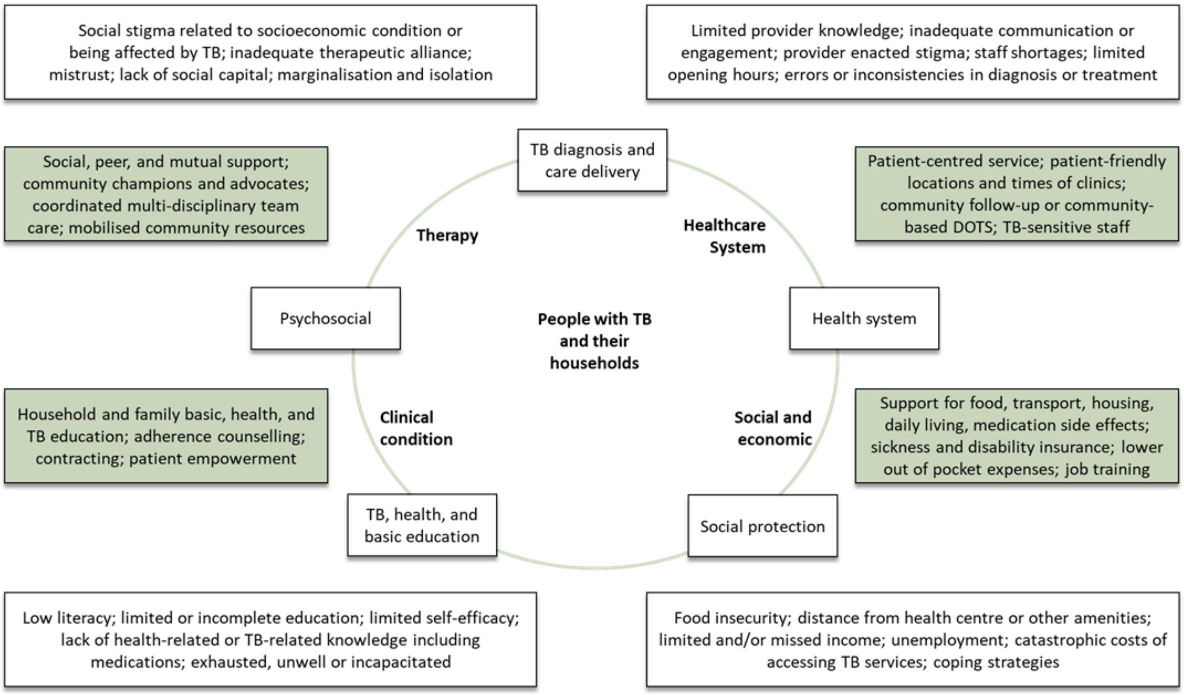

Figure 3. Conceptual framework: the barriers and facilitators to TB diagnosis and care. The central circle, which contains dimensions of medication adherence adapted from the World Health Organization[33], is surrounded by the five main categories of relevant potential interventions. The factors that may promote access to and engagement with TB services (facilitators) are shown in green boxes and factors that may threaten access and engagement (barriers) are shown in white boxes. Therapeutic alliance refers to strong provider-patient relationships. Although Project 2 would gather data on all five dimensions, the psychosocial and social protection dimensions were perceived by the project team to be most pertinent to development of the socioeconomic intervention and were selected for further focus and discussion during Project 2's workshop.

The final activity in Project 2 will be a one-day workshop bringing together the 40 key stakeholders (see Project 2 Workshop, *Extended data*)[29]. The morning section of the workshop will consist of interactive presentations from the project team and stakeholder group representatives (including leaders of national social protection schemes in Nepal), and discussions exploring and validating the barriers and opportunities identified during the pre-FGD survey and interviews during FGDs. The afternoon section of the workshop will consist of multi-sectoral working groups (≤10 diverse stakeholders) developing a shortlist of potential socioeconomic interventions for TB-affected households in Nepal. The shortlist is not intended to consist of defined, unalterable packages, which are immediately ready for trial implementation. Rather, the potential interventions selected are intended to consist of what stakeholders perceive to be key elements or ingredients of psychosocial and economic support for TB-affected households, which are feasible and locally-appropriate to the Nepalese context. The interventions will be presented to the group including strengths, weaknesses, and potential sources of funding for implementation.

The activities involved in this research form part of a process to develop and evaluate a complex, socioeconomic support intervention. In line with the Medical Research Council's guidance on process evaluation of complex interventions, we developed a logic model to illustrate the developmental stages of the intervention (Figure 4)[32].

This Wellcome research will aim to achieve the short-term outcomes described in the logic model. If follow-on funding is successfully obtained, the process to develop and implement the intervention and achieve the long-term outcomes will continue beyond this Wellcome research. This will include adaptation, piloting, and then large-scale randomised trial evaluation of the refined intervention.

Outcomes to be measured

This exploratory Wellcome Trust funded Seed Award in Science research in four districts of Nepal will:

i. characterise social determinants of TB by comparing poverty level, education level, food security, and other socioeconomic factors of people with TB (Cases) versus people without TB (Controls);

ii. provide new insight into the barriers, facilitators, and socioeconomic impact of being ill with TB and accessing TB diagnosis and care, and compare this in Cases diagnosed by ACF versus PCF; and

For peer review only - http://bmjopen.bmj.com/site/about/guidelines.xhtml

Wellcome Open Research 2020, 5:19 Last updated: 11 SEP 2020

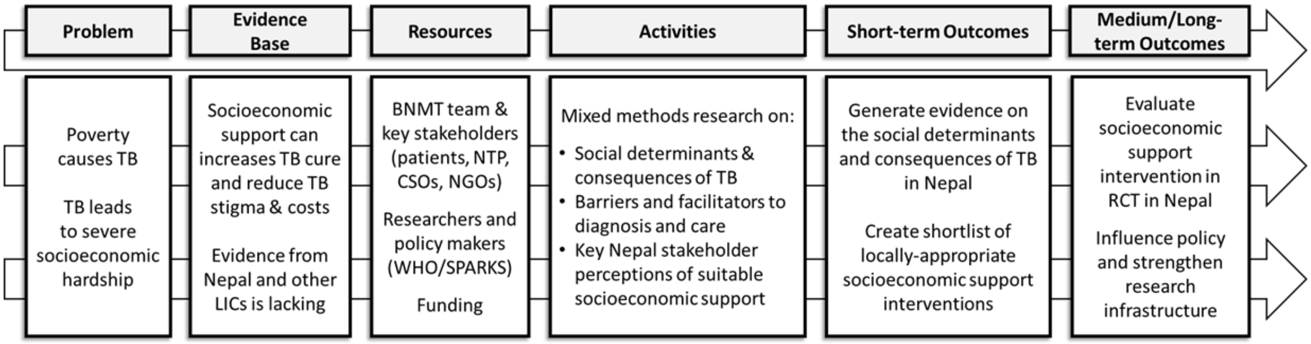

Figure 4. Logic model for research in Nepal to develop a locally-appropriate socioeconomic support intervention for TB-affected households. Abbreviations: CSOs = Civil Society Organisations; NGOs = Non-governmental organisations; NTP = National Tuberculosis Programme; WHO = World Health Organisation; SPARKS = Social Protection Action Research and Knowledge Sharing network (www.sparks.ki.se)

iii. generate a community-led shortlist of the most feasible, equitable, and locally-appropriate socioeconomic interventions for TB-affected households to mitigate the socioeconomic impact of TB.

Data collection and management

During the implementation of Project 1, information will be collected by BNMT district coordinators, community volunteers, and community mobilisers with support from the project team during visits to recruited patient households. This information includes but is not limited to socioeconomic, health, psychosocial, and behavioural data. This data will be collected on paper due to digital collection (e.g. on tablets or mobile phones) having issues with security and feasibility. The data of consenting TB patients will subsequently be linked with data from NTP's TB patient register as part of its routine surveillance data collection at the intervention and control areas, with pre-existing permission from the NTP.

During Project 2, key stakeholders identified by the scoping exercise will complete a short pre-FGD survey in person, participate in an FGD, and participate in a one-day workshop.

All paper-based copies, including medical records, informed consent forms and participant information leaflets, will contain only a unique study identifier for each participant. These documents will be stored in a locked room in the BNMT office. Data will be checked for consistency and completeness by the project manager and double-checked by the PI prior to entering into an encrypted access database. The database will be managed by the data management team at KNCV TB Foundation in The Netherlands in line with data collected during the IMPACT-TB project. The data will be protected by KNCV on a password-secured server with availability limited to only key members of the study team when required for analysis.

Data analysis and statistical plan

The quantitative data collected during the household visits of Project 1 will be analysed using simple descriptive statistics. Continuous costs data will be summarised by their arithmetic means and their 95% confidence intervals whether the data is Gaussian or non-Gaussian, because this approach is considered to be robust for health economics data analysis[6,34–36]. Furthermore, because of the skewed nature of some expenditure data, median values may be zero or close to zero limiting the descriptive usefulness of presenting median values. As described in the PI's previous research[6], any direct expenses, lost income, or annual income recorded as "zero" or missing will be replaced with the mean cost of each costs category, i.e. mean direct costs or lost income. The local currency, Nepalese rupee, will be converted into United States Dollars (conversion rate and date estimated through Oanda at time of data collection). Categorical data will be summarised as proportions with 95% confidence intervals.

With regards to analysis of household income and expenditure, the WHO TB Patients Costs Survey methods will be followed. In order to evaluate the optimal analytical strategy for costs data in Nepal, the analysis will compare and contrast the different approaches used in the WHO Survey to estimate household income (self-reported household consumption; self-reported household expenditure; self-reported household income; and estimated income based on household asset ownership and dwelling characteristics) and lost income (output approach of reported income pre- and during TB versus human capital approach of multiplying hours of work lost by hourly rate or daily in rate in cases of hospitalised patients).

Social determinants of TB including sociodemographic characteristics, socioeconomic position, stigma and social capital levels, and TB-related knowledge will be compared between ACF patients, PCF patients, and controls using Chi-squared test, Pearson's test, one-way ANOVA, and multiple logistic regression models where appropriate. No comparison will be made between patients and controls concerning healthcare expenditure as this data was not collected from controls. Statistical analysis will be performed using the statistical software package STATA v13.1 (Statacorp, TX, USA).

For peer review only - http://bmjopen.bmj.com/site/about/guidelines.xhtml

Wellcome Open Research 2020, 5:19 Last updated: 11 SEP 2020

The Framework method of thematic analysis will be used to manage and analyse data from Project 2 via the NVivo qualitative software package (Version 12) as per published social policy and tuberculosis research[21–24]. Specifically, two researchers (KD and TW) will familiarize themselves with the data through successive reading of transcripts; use both open and closed first order data coding to label data within NVivo; group codes together into a second order codebook of themes and sub-themes. Themes and subthemes will then be further stratified by third order coding to the level at which they predominantly occurred (e.g. individual or household level, health systems level, community level, or governmental level). The primary approach to codes, themes, and sub-themes will be deductive (top down), being informed by our conceptual framework and preliminary analysis of data from Project 1 relating to the social determinants and consequences identified. As coding continues, a secondary approach will integrate inductive (bottom up) coding in order to be iterative, responsive, and flexible as further data becomes available and is collated following each successive FGD.

Plans for dissemination of study findings

The intended research outputs of this work are to: i) present the interim and final findings at the International Union Against TB and Lung Disease in October 2019 and October 2020, respectively; ii) publish, by June 2020, at least two papers in high-impact, peer-reviewed journals concerning the socioeconomic impact of accessing TB care in Nepal and the collaborative development of a shortlist of locally-appropriate socioeconomic interventions in Nepal; iii) feedback findings to the IMPACT-TB team, key stakeholders (including NTP and TB civil-society), the SPARKS (Social Protection Action Research and Knowledge Sharing) network, and WHO; iv) consolidate a close collaboration, good working relationship, and strong research infrastructure between BNMT and the NTP; and iv) to develop the protocol for a robust, large-scale randomised controlled trial to evaluate socioeconomic support for TB-affected households using the evidence generated by this mixed-methods study.

Study status

Data collection for Project 1 during household visits began in May 2018 and is nearly complete at the time of writing with only TB treatment outcome data still being collected. Data collection for Project 2 during pre-FGD surveys and FGDs began in August 2018 and was completed in July 2019. The national workshop with key stakeholders was conducted on 11 and 12 September 2019 in Kathmandu, Nepal.

Data analysis, write-up, and dissemination of findings will begin in February 2020.

Discussion

The overarching pledge of the Sustainable Development Goals (SDGs) is to “leave no one behind”. In 2018, over 3 million people with tuberculosis (TB) were not diagnosed, not notified, or their quality of care was unknown. In the same year, 1.5 million people with TB died and nearly a fifth of people diagnosed with TB did not have a successful treatment outcome[16]. While millions of people with TB continue to be left behind, the SDG pledge is far from being realised.

Despite renewed interest in addressing social determinants of tuberculosis, there remain stark global inequalities in disease burden and access to TB care[8]. At a population level, LICs bear the highest TB prevalence[16]. At an individual level, people with TB are often vulnerable, impoverished, and their households suffer disproportionate financial shock due to their illness[6,13]. To eliminate such gross disparity, SDG slogans must be turned into actions.

WHO’s 2015 End TB Strategy acknowledges the need to reduce inequalities in TB prevention and care. A key component of Pillar 2 (Bold policies and supportive systems) of the strategy is social protection and poverty alleviation to reduce catastrophic costs of TB-affected households and improve TB outcomes[2]. However, there is minimal evidence to guide this policy change.

This mixed-methods will generate evidence concerning the socioeconomic position of TB-affected households, the impact that having TB disease has on that position and explore the coping strategies that households use to mitigate the impact of the disease. Moreover, this research will: use methods to measure costs of TB-affected households to which members of the study team contributed as part of the WHO Task Force on Catastrophic Costs of Tuberculosis; and provide the first known comparison of the socioeconomic impact of TB on people with TB diagnosed through PCF versus ACF. The case-control element of Project 1 will allow comparison of the sociodemographic characteristics, socioeconomic position, stigma and social capital levels, and TB-related knowledge between people with TB (Cases) and people without TB (Controls).

The further significance of the study lies in its development of a shortlist of a locally-generated intervention to provide socioeconomic support to TB-affected households. Through collaboration with diverse stakeholders in Nepal from patients to NTP managers to civil-society representatives, it is hoped that the shortlist created will feature interventions that are both locally-appropriate and feasible. Furthermore, this tight collaboration should aid design and implementation of a larger, randomised-controlled trial, and also future translation of research findings into national policy in Nepal. Beyond the national impact, these findings and those of the future trial will also offer evidence for scale-up of socioeconomic support in other resource-limited countries with a high TB burden. Complementary to pills and tests, this socioeconomic support will be an essential part of eliminating TB by 2050.

Conclusions

This mixed-methods study will fill this existing evidence gap by examining the costs of accessing TB care at a household level through longitudinal application of an adapted WHO TB Patient Costs Survey throughout TB treatment. This data will be enriched by collecting complementary data on household socioeconomic position, coping strategies, food insecurity,

For peer review only - http://bmjopen.bmj.com/site/about/guidelines.xhtml

Wellcome Open Research 2020, 5:19 Last updated: 11 SEP 2020

TB knowledge, social capital, and quality of life. The findings will then feed into a short survey and semi-structured FGDs with key stakeholders in Nepal to consider, at a local, regional, and national level, what are the leading barriers and facilitators to accessing and engaging in TB care, and what might be the most locally-appropriate interventions to address the socioeconomic impact of having TB disease.

Data availability

Underlying data

No data are associated with this article

Extended data

Open Science Framework: Research protocol for a mixed-methods study to characterise and address the socioeconomic impact of accessing TB diagnosis and care in Nepal. https://doi.org/10.17605/OSF.IO/6TC4F[29]

This project contains the following extended data:

- Final_Wellcome Seed Award Project 1 Patient Interview v8.pdf
- Final Patient _Wellcome Seed Award Project 1 Consent Form NEPALI.pdf
- Final Patient _Wellcome Seed Award Project 1 Interview v8 NEPALI.pdf
- Final Patient _Wellcome Seed Award Project 1 Patient Info Leaflet NEPALI.pdf
- Wellcome Seed Award Project 1 Patient Consent Form.pdf
- Wellcome Seed Award Project 1 Patient Information Leaflet ENGLISH.pdf
- Wellcome Seed Award Project 2 Survey and Focus Group Consent Form.pdf
- Wellcome Seed Award Project 2 Survey and Focus Group Participant Information Leaflet.pdf
- Wellcome Seed Award Project 2 Survey v4 20180525.pdf
- Wellcome Seed Award Project 2 Workshop Consent Form.pdf
- Wellcome Seed Award Project 2 Workshop Participant Information Leaflet.pdf

Data are available under the terms of the Creative Commons Zero "No rights reserved" data waiver (CC0 1.0 Public domain dedication).

References

1. Raviglione M, Krech R: **Tuberculosis: still a social disease.** *Int J Tuberc Lung Dis.* 2011; **15**(Suppl 2): 6–8.
PubMed Abstract | Publisher Full Text

2. Bhargava A, Pai M, Bhargava M, *et al.*: **Can social interventions prevent tuberculosis?: the Papworth experiment (1918–1943) revisited.** *Am J Respir Crit Care Med.* 2012; **186**(5): 442–449.
PubMed Abstract | Publisher Full Text

3. Janssens JP, Rieder HL: **An ecological analysis of incidence of tuberculosis and *per capita* gross domestic product.** *Eur Respir J.* 2006; **32**(5): 1415–1416.
PubMed Abstract | Publisher Full Text

4. Dye C, Lönnroth K, Jaramillo E, *et al.*: **Trends in tuberculosis incidence and their determinants in 134 countries.** *Bull World Health Organ.* 2009; **87**(9): 683–691.
PubMed Abstract | Publisher Full Text | Free Full Text

5. World Health Organisation's: **End TB Strategy.** 2020.
Reference Source

6. Wingfield T, Boccia D, Tovar M, *et al.*: **Defining catastrophic costs and comparing their importance for adverse tuberculosis outcome with multi-drug resistance: a prospective cohort study, Peru.** *PLoS Med.* 2014; **11**(7): e1001675.
PubMed Abstract | Publisher Full Text | Free Full Text

7. Wingfield T, Tovar MA, Huff D, *et al.*: **Socioeconomic support to improve initiation of tuberculosis preventive therapy and increase tuberculosis treatment success in Peru: a household-randomised, controlled evaluation.** *Lancet.* Academy of Medical Sciences Young Investigator Award. 2017; **389**(6).
Publisher Full Text

8. Wingfield T, Tovar MA, Datta S, *et al.*: **Addressing social determinants to end tuberculosis.** *Lancet.* 2018; **391**(10126): 1129–1132.
PubMed Abstract | Publisher Full Text

9. Wingfield T, Schumacher SG, Sandhu G, *et al.*: **The seasonality of tuberculosis, sunlight, vitamin D, and household crowding.** *J Infect Dis.* 2014; **210**(5): 774–83.
PubMed Abstract | Publisher Full Text | Free Full Text

10. Williamson J, Ramirez R, Wingfield T: **Health, healthcare access, and use of traditional versus modern medicine in remote Peruvian Amazon communities: a descriptive study of knowledge, attitudes, and practices.** *Am J Trop Med Hyg.* 2015; **92**(4): 857–864.
PubMed Abstract | Publisher Full Text | Free Full Text

11. Boccia D, Pedrazzoli D, Wingfield T, *et al.*: **Towards cash transfer interventions for tuberculosis prevention, care and control: key operational challenges and research priorities.** *BMC Infect Dis.* 2016; **16**: 307.
PubMed Abstract | Publisher Full Text | Free Full Text

12. Wingfield T, Boccia D, Tovar MA, *et al.*: **Designing and implementing a socioeconomic intervention to enhance TB control: operational evidence from the CRESIPT project in Peru.** *BMC Public Health.* 2015; **15**(1): 810.
PubMed Abstract | Publisher Full Text | Free Full Text

13. Wingfield T, Tovar MA, Huff D, *et al.*: **The economic effects of supporting tuberculosis-affected households in Peru.** *Eur Respir J.* 2016; **48**(5): 1396–1410.
PubMed Abstract | Publisher Full Text | Free Full Text

14. Wingfield T, Tovar MA, Huff D, *et al.*: **Beyond pills and tests: addressing the social determinants of tuberculosis.** *Clin Med (Lond).* 2016; **16**(Suppl 6): s79–s91.
PubMed Abstract | Publisher Full Text | Free Full Text

15. Wingfield T, Tovar MA, Huff D, *et al.*: **A randomized controlled study of socioeconomic support to enhance tuberculosis prevention and treatment, Peru.** *Bull World Health Organ.* 2017; **95**(4): 270–280.
PubMed Abstract | Publisher Full Text | Free Full Text

16. World Health Organisation: **Global Tuberculosis Report 2019.** WHO. 2019.
Reference Source

17. Gurung GN, Chhetri PS, Jha N: **Economic impact of pulmonary tuberculosis on patients and their families of Dharan municipality, Nepal.** *Nepal Med Coll J.* 2012; **14**(3): 196–198.
PubMed Abstract

18. Bam TS, Gunneberg C, Chamroonsawasdi K, *et al.*: **Factors affecting patient adherence to DOTS in urban Kathmandu, Nepal.** *Int J Tuberc Lung Dis.* 2006; **10**(3): 270–276.
PubMed Abstract

19. Kirwan DE, Nicholson BD, Baral SC, *et al.*: **The social reality of migrant men with tuberculosis in Kathmandu: implications for DOT in practice.** *Trop Med Int Health.* 2009; **14**(12): 1442–1447.
PubMed Abstract | Publisher Full Text

20. Ramsay A, Al-Agbhari N, Scherchand J, *et al.*: **Direct patient costs associated with tuberculosis diagnosis in Yemen and Nepal.** *Int J Tuberc Lung Dis.* 2010; **14**(2): 165–170.
PubMed Abstract

21. Mishra P, Hansen EH, Sabroe S, *et al.*: **Socio-economic status and adherence to tuberculosis treatment: a case-control study in a district of Nepal.** *Int J Tuberc Lung Dis.* 2005; **9**(10): 1134–1139.
PubMed Abstract

For peer review only - http://bmjopen.bmj.com/site/about/guidelines.xhtml

22. **Measuring patient costs to monitor progress towards the target to eliminate catastrophic costs and help design social protection and UHC.** Meeting of the WHO Global Task Force on TB Impact Measurement, 19–21 April 2016. GTB/WHO. Reference Source

23. WHO: **Protocol for questionnaire to determine direct and indirect costs due to TB and to estimate proportion of TB-affected households experiencing catastrophic costs: field testing version**. Geneva: World Health Organization, 2015. Reference Source

24. Baral SC, Aryal Y, Bhattrai R, *et al.*: **The importance of providing counselling and financial support to patients receiving treatment for multi-drug resistant TB: mixed method qualitative and pilot intervention studies.** *BMC Public Health.* 2014; **14**: 46.
PubMed Abstract | Publisher Full Text | Free Full Text

25. Oliver K, Innvar S, Lorenc T, *et al.*: **A systematic review of barriers to and facilitators of the use of evidence by policymakers.** *BMC Health Serv Res.* 2014; **14**: 2.
PubMed Abstract | Publisher Full Text | Free Full Text

26. Lavis JN, Boyko JA, Oxman AD, *et al.*: **SUPPORT Tools for evidence-informed health Policymaking (STP) 14: Organising and using policy dialogues to support evidence-informed policymaking.** *Health Res Policy Syst.* 2009; **7**(Suppl 1): S14.
PubMed Abstract | Publisher Full Text | Free Full Text

27. Lomas J: **Using 'linkage and exchange' to move research into policy at a Canadian foundation.** *Health Aff (Millwood).* 2000; **19**(3): 236–40.
PubMed Abstract | Publisher Full Text

28. Lau R, Stevenson F, Ong BN, *et al.*: **Achieving change in primary care--causes of the evidence to practice gap: systematic reviews of reviews.** *Implement Sci.* 2016; **11**: 40.
PubMed Abstract | Publisher Full Text | Free Full Text

29. Wingfield T: **Research protocol for a mixed-methods study to characterise and address the socioeconomic impact of accessing TB diagnosis and care in Nepal.** 2020.
http://www.doi.org/10.17605/OSF.IO/6TC4F

30. Gospodarevskaya E, Tulloch O, Bunga C, *et al.*: **Patient costs during tuberculosis treatment in Bangladesh and Tanzania: the potential of shorter regimens.** *Int J Tuberc Lung Dis.* 2014; **18**(7): 810–17.
PubMed Abstract | Publisher Full Text

31. Gama E, Madan J, Langley I, *et al.*: **Economic evaluation of a shortened standardised treatment regimen of antituberculosis drugs for patients with multidrug-resistant tuberculosis (STREAM): study protocol.** *BMJ Open.* 2016; **6**(10): e014386.
PubMed Abstract | Publisher Full Text | Free Full Text

32. Weaver MS, Lonnroth K, Howard SC, *et al.*: **Interventions to improve adherence to treatment for paediatric tuberculosis in low- and middle-income countries: a systematic review and meta-analysis.** *Bull World Health Organ.* 2015; 93(10): 700–711B.
PubMed Abstract | Publisher Full Text | Free Full Text

33. Moore G: **Process evaluation of complex interventions.** Decipher.Uk.Net. 2014; 19–75.

34. Barter DM, Agboola SO, Murray MB, *et al.*: **Tuberculosis and poverty: the contribution of patient costs in sub-Saharan Africa--a systematic review.** *BMC Public Health.* 2012; **12**: 980.
PubMed Abstract | Publisher Full Text | Free Full Text

35. Thompson SG, Barber JA: **How should cost data in pragmatic randomised trials be analysed?** *BMJ.* 2000; **320**(7243): 1197–1200.
PubMed Abstract | Publisher Full Text | Free Full Text

36. Barber JA, Thompson SG: **Analysis and interpretation of cost data in randomised controlled trials: review of published studies.** *BMJ.* 1998; **317**(7167): 1195–1200.
PubMed Abstract | Publisher Full Text | Free Full Text

For peer review only

For peer review only - http://bmjopen.bmj.com/site/about/guidelines.xhtml

Open Peer Review

Current Peer Review Status: ✔ ✔

Version 2

Reviewer Report 11 September 2020

https://doi.org/10.21956/wellcomeopenres.17629.r39129

© **2020 Boccia D.** This is an open access peer review report distributed under the terms of the Creative Commons Attribution License, which permits unrestricted use, distribution, and reproduction in any medium, provided the original work is properly cited.

✔ **Delia Boccia**
Faculty of Public Health and Policy, London School of Hygiene and Tropical Medicine, London, UK

I confirm that the authors have appropriately and convincingly addressed all my comments.

Competing Interests: No competing interests were disclosed.

Reviewer Expertise: Social determinants of TB, social protection.

I confirm that I have read this submission and believe that I have an appropriate level of expertise to confirm that it is of an acceptable scientific standard.

Reviewer Report 07 August 2020

https://doi.org/10.21956/wellcomeopenres.17629.r39128

© **2020 Shete P.** This is an open access peer review report distributed under the terms of the Creative Commons Attribution License, which permits unrestricted use, distribution, and reproduction in any medium, provided the original work is properly cited.

✔ **Priya B. Shete**
University of California San Francisco, San Francisco, CA, USA

No further comments. I appreciate the authors attention to my previous suggestions and feel that they have taken care to improve the paper with these details.

Competing Interests: No competing interests were disclosed.

Reviewer Expertise: Implementations science, tuberculosis, social protection.

For peer review only - http://bmjopen.bmj.com/site/about/guidelines.xhtml

I confirm that I have read this submission and believe that I have an appropriate level of expertise to confirm that it is of an acceptable scientific standard.

Version 1

Reviewer Report 26 February 2020

https://doi.org/10.21956/wellcomeopenres.17181.r37807

© 2020 Shete P. This is an open access peer review report distributed under the terms of the Creative Commons Attribution License, which permits unrestricted use, distribution, and reproduction in any medium, provided the original work is properly cited.

Priya B. Shete
University of California San Francisco, San Francisco, CA, USA

The major area for improvement with this protocol would be more discussion in the methods about how the qualitative data will be coded and subsequently used to then generate a menu of feasible options for pilot implementation and evaluation to overcome targeted barriers. I would appreciate further discussion of the conceptual framework that informs the data collection tools/interview guides in Project 2. As Project 2 is particularly important for identifying targeted barriers and facilitators of care for future interventions, I would also like to see what deterministic framework or model is being used to develop those interventions. Any reference to frameworks from implementation science or social epidemiology would perhaps be helpful.

Is the rationale for, and objectives of, the study clearly described?
Yes

Is the study design appropriate for the research question?
Yes

Are sufficient details of the methods provided to allow replication by others?
Partly

Are the datasets clearly presented in a useable and accessible format?
Not applicable

Competing Interests: No competing interests were disclosed.

Reviewer Expertise: Implementations science, tuberculosis, social protection.

I confirm that I have read this submission and believe that I have an appropriate level of expertise to confirm that it is of an acceptable scientific standard, however I have significant reservations, as outlined above.

For peer review only - http://bmjopen.bmj.com/site/about/guidelines.xhtml

Author Response 11 Jun 2020

Tom Wingfield, Liverpool School of Tropical Medicine, UK

We are indebted to Reviewer 2 for these excellent comments, which we have addressed and which we now believe serve to strengthen the protocol. It is important to note that this Wellcome Trust funded study began in 2018 and data collection had already been completed prior to submission of the protocol to Wellcome Open Research. When the research team became aware of Wellcome Open Research, we approached the editorial team who advised that it was still appropriate to submit a study protocol despite data collection being complete. While this is important for critical peer review of the protocol it does, unfortunately, mean that we are unable to adjust parts of the protocol that have been completed and written-up including, for example, the coding strategy.

However, we were able to address Reviewer 2's comments by providing further detail about the coding techniques used and necessary clarification about how the quantitative and qualitative data fed informed outline of the design of the socioeconomic support interventions. In addition, we omitted to include the conceptual framework and logic model that we employed for this research, which we have now rectified and, again, this has helped to improve the protocol and will also be included in subsequent, related publications. Thank you to the Reviewer for their constructive review.

Study activities section: "Project 2 will use and develop mixed methods research techniques [10–15] to conduct a pre-FGD survey, FGDs, and workshop with approximately 40 key stakeholders in Nepal to identify the socioeconomic impact, barriers and facilitators to accessing and engaging with TB diagnosis and care. To inform the design of the FGDs and workshop, we created a conceptual framework for the barriers and facilitators to TB diagnosis and care, which was adapted from a World Health Organisation framework for medication adherence (Figure 3).[30]

(PLEASE SEE FIGURE IN UPDATED PROTOCOL)

Figure 3. Conceptual framework: the barriers and facilitators to TB diagnosis and care.

The central circle, which contains dimensions of medication adherence adapted from the World Health Organization[30], is surrounded by the five main categories of relevant potential interventions. The factors that may promote access to and engagement with TB services (facilitators) are shown in green boxes and factors that may threaten access and engagement (barriers) are shown in white boxes. Therapeutic alliance refers to strong provider-patient relationships. Although Project 2 would gather data on all five dimensions, the psychosocial and social protection dimensions were perceived by the project team to be most pertinent to development of the socioeconomic intervention and were selected for further focus and discussion during Project 2's workshop."

Study activities section: "The final activity in Project 2 will be a one-day workshop bringing together the 40 key stakeholders (see Project 2 Workshop, Extended data) [29] . The morning section of the workshop will consist of interactive presentations from the project team and stakeholder group representatives (including leaders of national social protection schemes

in Nepal), and discussions exploring and validating the barriers and opportunities identified during the pre-FGD survey and interviews during FGDs. The afternoon section of the workshop will consist of multi-sectoral working groups ($\leq$**10 diverse stakeholders) developing a shortlist of potential socioeconomic interventions for TB-affected households in Nepal. The shortlist is not intended to consist of defined, unalterable packages, which are immediately ready for trial implementation. Rather, the potential interventions selected are intended to consist of what stakeholders perceive to be key elements or ingredients of psychosocial and economic support for TB-affected households, which are feasible and locally-appropriate to the Nepalese context. The interventions will be presented to the group including strengths, weaknesses, and potential sources of funding for implementation.**

The activities involved in this research form part of a process to develop and evaluate a complex, socioeconomic support intervention. In line with the Medical Research Council's guidance on process evaluation of complex interventions, we developed a logic model to illustrate the developmental stages of the intervention (Figure 4).[31]

(PLEASE SEE FIGURE IN UPDATED PROTOCOL)

Figure 4. Logic model for research in Nepal to develop a locally-appropriate socioeconomic support intervention for TB-affected households.

This Wellcome research will aim to achieve the short-term outcomes described in the logic model. If follow-on funding is successfully obtained, the process to develop and implement the intervention and achieve the long-term outcomes will continue beyond this Wellcome research. This will include adaptation, piloting, and then large-scale randomised trial evaluation of the refined intervention."

Data analysis and statistical plan section: "The Framework method of thematic analysis will be used to manage and analyse data from Project 2 via the NVivo qualitative software package (Version 12) as per published social policy and tuberculosis research [21–24] . Specifically, two researchers (KD and TW) will familiarize themselves with the data through successive reading of transcripts; use both open and closed first order data coding to label data within NVivo; group codes together into a second order codebook of themes and sub-themes. Themes and subthemes will then be further stratified by third order coding to the level at which they predominantly occurred (e.g. individual or household level, health systems level, community level, or governmental level). The primary approach to codes, themes, and sub-themes will be deductive (top down), being informed by our conceptual framework and preliminary analysis of data from Project 1 relating to the social determinants and consequences identified. As coding continues, a secondary approach will integrate inductive (bottom up) coding in order to be iterative, responsive, and flexible as further data becomes available and is collated following each successive FGD."

Competing Interests: None.

Reviewer Report 21 February 2020

https://doi.org/10.21956/wellcomeopenres.17181.r37826

© 2020 Boccia D. This is an open access peer review report distributed under the terms of the Creative Commons Attribution License, which permits unrestricted use, distribution, and reproduction in any medium, provided the original work is properly cited.

Delia Boccia
Faculty of Public Health and Policy, London School of Hygiene and Tropical Medicine, London, UK

This paper describes in detail the protocol of a study aiming at understanding better the social determinants of TB as well as the financial consequences of TB. The study is very relevant: it is epidemiologically sound and the rational for it is clear and strongly supported by the existing literature.

There are two gaps that – in my opinion – should be better filled to make the protocol stronger:

1. The selection of controls for the case-control study. This requires a further dissertation about how practically controls will be selected and how the authors are planning to minimise selection biases. Authors explain why an age-sex matching approach could not be adopted; however, the description of the selection of controls remain elusive which triggers questions like: what sampling strategy will be adopted? How randomness as well as representativeness will be respected? General population controls may be a good option for answering questions around the social determinants of TB; however, I am not convinced they are also the best choice when looking at catastrophic health expenditures. Obviously healthy controls will be better off and wonder whether this second research question could be not better addressed by selecting hospital controls or among people who are ill-affected (albeit not from TB). I suggest the authors to better justify their choices and discuss why alternative options have been dismissed.

2. The qualitative work should include a stronger effort to address not just barriers to access to TB care, but also barriers to existing social protection schemes experienced by TB-patients. It feels like a gigantic missed opportunity if not embedded into the existing plans. If indeed the authors are planning to ascertain existing social protection options for TB patients and access can be maximised, then this should be explicitly stated in the paper.

Is the rationale for, and objectives of, the study clearly described?
Yes

Is the study design appropriate for the research question?
Partly

Are sufficient details of the methods provided to allow replication by others?
Partly

Are the datasets clearly presented in a useable and accessible format?

For peer review only - http://bmjopen.bmj.com/site/about/guidelines.xhtml

Not applicable

Competing Interests: No competing interests were disclosed.

Reviewer Expertise: Social determinants of TB, social protection.

I confirm that I have read this submission and believe that I have an appropriate level of expertise to confirm that it is of an acceptable scientific standard, however I have significant reservations, as outlined above.

Author Response 11 Jun 2020

Tom Wingfield, Liverpool School of Tropical Medicine, UK

1. The selection of controls for the case-control study. This requires a further dissertation about how practically controls will be selected and how the authors are planning to minimise selection biases. Authors explain why an age-sex matching approach could not be adopted; however, the description of the selection of controls remain elusive which triggers questions like: what sampling strategy will be adopted? How randomness as well as representativeness will be respected? General population controls may be a good option for answering questions around the social determinants of TB; however, I am not convinced they are also the best choice when looking at catastrophic health expenditures. Obviously healthy controls will be better off and wonder whether this second research question could be not better addressed by selecting hospital controls or among people who are ill-affected (albeit not from TB). I suggest the authors to better justify their choices and discuss why alternative options have been dismissed.

Author response: Thank you for raising this important issue with which we wholeheartedly agree. It is important to note that this Wellcome Trust funded study began in 2018 and data collection had already been completed prior to submission of the protocol to Wellcome Open Research. When the research team became aware of Wellcome Open Research, we approached the editorial team who advised that it was still appropriate to submit a study protocol despite data collection being complete. While this is important for critical peer review of the protocol it does, unfortunately, mean that we are unable to adjust the protocol including, for example, sampling strategies at this stage. However, we have attempted to respond to the reviewer's comments about sampling strategy and control population randomness and representativeness. We have also made it clearer that the data from controls will only be used to characterise social determinants of TB and there will be no analysis comparing health expenditure in controls vs people with TB, which we agree would be inappropriate with general population controls.

Study population section: "Concurrently, 100 people without TB from the study sites (controls) will be invited to participate with written, informed consent (see Project 1 Interview Healthy Control Consent Form, Extended data)[29] . Due to constraints in study budget, duration, and field logistics, it will not be possible to age and sex match cases and controls nor to randomly select controls using geospatial or other household randomisation techniques. Therefore, a convenience sampling strategy was used. In order to be as widely representative of and generalisable to the background population as

possible, following project team and community mobiliser discussions in the study sites, we opted to recruit control participants from diverse locations within the districts, which were attended by a broad demographic cross-section of the local population in terms of age, gender, and socioeconomic position. These diverse study site locations include: tea houses, primary healthcare centres, antenatal and immunization clinics, door-to-door visits following sputum camps (e.g. people who tested negative for TB), and public gathering places. Interviewers will visit the control recruitment locations at similar times of the morning and aim to consecutively recruit all individuals in attendance at that location. Similar numbers of participants from each recruitment location will be recruited until the sample size of 100 people without TB is reached. We acknowledge that convenience sampling may be associated with a higher likelihood of a non-generalisable control cohort than other techniques. To try to address this, the survey used to collect data from the controls has incorporated multiple questions concerning sociodemographic variables, including education level, occupation, amenities, and assets, from the most recent version of the Nepal Household Survey, which is publicly available. Descriptive analysis will evaluate whether the controls recruited are representative of the wider population in those districts through comparison of their sociodemographic data with respondents to the national survey in the same districts. Any differences between the control population and background population will then be highlighted transparently in corresponding research outputs and publications."

"Data analysis and statistical plan section: "Social determinants of TB including sociodemographic characteristics, socioeconomic position, stigma and social capital levels, and TB-related knowledge will be compared between ACF patients, PCF patients, and controls using Chi-squared test, Pearson's test, one-way ANOVA, and multiple logistic regression models where appropriate. No comparison will be made between patients and controls concerning healthcare expenditure as this data was not collected from controls."

2. The qualitative work should include a stronger effort to address not just barriers to access to TB care, but also barriers to existing social protection schemes experienced by TB-patients. It feels like a gigantic missed opportunity if not embedded into the existing plans. If indeed the authors are planning to ascertain existing social protection options for TB patients and access can be maximised, then this should be explicitly stated in the paper.

Author Response: We agree entirely with the reviewer and are grateful for this apposite point. Within the confines of not being able to alter the protocol given that data collection is now completed, we have clarified this important issue in the protocol to explain that, across Projects 1 and 2, quantitative and qualitative data concerning social protection in Nepal was gathered, including the coverage, target recipients, barriers to access, and limitations of existing packages.

Study activities section: "***The interviews will be structured (see Project 1 Interview, Extended data)[29] and gather data on: i) socioeconomic position, evaluated by a multi-dimensional poverty score[6, 9, 13, 15] assessing dwelling characteristics, assets, and access to amenities; ii) household structure, including distribution of age, sex, and employment of household members; iii) food expenditure and security; iv) costs of engaging with TB care including direct costs (e.g. medicines, clinic visits, food, and travel) and indirect costs (e.g.***

lost income), which will be evaluated using an adapted version of WHO's TB Patient Costs Survey integrated into the interview [22, 23] ; v) coping strategies including dissaving (e.g. selling assets), schooldays lost, and temporary income-generating activities; vi) TB-related knowledge including understanding of transmission, prevention, and treatment of TB; vii) psychosocial situation evaluated through questions relating to social capital, quality of life, and stigma (Controls without TB will not be asked any questions about the impact that having TB disease has on their psychosocial situation); and viii) access and uptake of existing social protection schemes (whether TB-specific or TB-inclusive) and support for TB-affected households, evaluated through use of both closed ranking and open free-text questions to establish what socioeconomic and other support people with TB and their households receive or would like to receive."

Study activities section: "A short individual pre-FGD survey (see Project 2 Survey, Extended data) [29] will be provided to participants prior to the initiation of an FGD. The survey will detail: participants' demographics; the stakeholder group to which the participant belongs; and their opinions on community, patient, health system, existing social protection schemes, and wider obstacles to achieving successful TB treatment outcomes.

The FGDs will be semi-structured and incorporate open-ended questions concerning barriers and facilitators to accessing and engaging with TB care in Nepal, and existing platforms and potential opportunities to mitigate these barriers including social protection schemes (see Project 2 Focus Groups, Extended data) [29] .

Study activities section: "The final activity in Project 2 will be a one-day workshop bringing together the 40 key stakeholders (see Project 2 Workshop, Extended data) [29] . The morning section of the workshop will consist of interactive presentations from the project team and stakeholder group representatives (including leaders of national social protection schemes in Nepal), and discussions exploring and validating the barriers and opportunities identified during the pre-FGD survey and interviews during FGDs."

Competing Interests: None.

For peer review only - http://bmjopen.bmj.com/site/about/guidelines.xhtml

COREQ (COnsolidated criteria for REporting Qualitative research) Checklist

A checklist of items that should be included in reports of qualitative research. You must report the page number in your manuscript where you consider each of the items listed in this checklist. If you have not included this information, either revise your manuscript accordingly before submitting or note N/A.

Topic	Item No.	Guide Questions/Description	Reported on Page No.
**Domain 1: Research team and reflexivity**			
Personal characteristics			
Interviewer/facilitator	1	Which author/s conducted the interview or focus group?	
Credentials	2	What were the researcher's credentials? E.g. PhD, MD	
Occupation	3	What was their occupation at the time of the study?	
Gender	4	Was the researcher male or female?	
Experience and training	5	What experience or training did the researcher have?	
Relationship with participants			
Relationship established	6	Was a relationship established prior to study commencement?	
Participant knowledge of the interviewer	7	What did the participants know about the researcher? e.g. personal goals, reasons for doing the research	
Interviewer characteristics	8	What characteristics were reported about the inter viewer/facilitator? e.g. Bias, assumptions, reasons and interests in the research topic	
**Domain 2: Study design**			
Theoretical framework			
Methodological orientation and Theory	9	What methodological orientation was stated to underpin the study? e.g. grounded theory, discourse analysis, ethnography, phenomenology, content analysis	
Participant selection			
Sampling	10	How were participants selected? e.g. purposive, convenience, consecutive, snowball	
Method of approach	11	How were participants approached? e.g. face-to-face, telephone, mail, email	
Sample size	12	How many participants were in the study?	
Non-participation	13	How many people refused to participate or dropped out? Reasons?	
Setting			
Setting of data collection	14	Where was the data collected? e.g. home, clinic, workplace	
Presence of non-participants	15	Was anyone else present besides the participants and researchers?	
Description of sample	16	What are the important characteristics of the sample? e.g. demographic data, date	
Data collection			
Interview guide	17	Were questions, prompts, guides provided by the authors? Was it pilot tested?	
Repeat interviews	18	Were repeat inter views carried out? If yes, how many?	
Audio/visual recording	19	Did the research use audio or visual recording to collect the data?	
Field notes	20	Were field notes made during and/or after the inter view or focus group?	
Duration	21	What was the duration of the inter views or focus group?	
Data saturation	22	Was data saturation discussed?	
Transcripts returned	23	Were transcripts returned to participants for comment and/or	

For peer review only

Topic	Item No.	Guide Questions/Description	Reported on Page No.
		correction?	
**Domain 3: analysis and findings**			
Data analysis			
Number of data coders	24	How many data coders coded the data?	
Description of the coding tree	25	Did authors provide a description of the coding tree?	
Derivation of themes	26	Were themes identified in advance or derived from the data?	
Software	27	What software, if applicable, was used to manage the data?	
Participant checking	28	Did participants provide feedback on the findings?	
Reporting			
Quotations presented	29	Were participant quotations presented to illustrate the themes/findings? Was each quotation identified? e.g. participant number	
Data and findings consistent	30	Was there consistency between the data presented and the findings?	
Clarity of major themes	31	Were major themes clearly presented in the findings?	
Clarity of minor themes	32	Is there a description of diverse cases or discussion of minor themes?	

Developed from: Tong A, Sainsbury P, Craig J. Consolidated criteria for reporting qualitative research (COREQ): a 32-item checklist for interviews and focus groups. *International Journal for Quality in Health Care*. 2007. Volume 19, Number 6: pp. 349 – 357

Once you have completed this checklist, please save a copy and upload it as part of your submission. DO NOT include this checklist as part of the main manuscript document. It must be uploaded as a separate file.

For peer review only
